# Structural and non-coding variants increase the diagnostic yield of clinical whole genome sequencing for rare diseases

**DOI:** 10.1186/s13073-023-01240-0

**Published:** 2023-11-09

**Authors:** Alistair T. Pagnamenta, Carme Camps, Edoardo Giacopuzzi, John M. Taylor, Mona Hashim, Eduardo Calpena, Pamela J. Kaisaki, Akiko Hashimoto, Jing Yu, Edward Sanders, Ron Schwessinger, Jim R. Hughes, Gerton Lunter, Helene Dreau, Matteo Ferla, Lukas Lange, Yesim Kesim, Vassilis Ragoussis, Dimitrios V. Vavoulis, Holger Allroggen, Olaf Ansorge, Christian Babbs, Siddharth Banka, Benito Baños-Piñero, David Beeson, Tal Ben-Ami, David L. Bennett, Celeste Bento, Edward Blair, Charlotte Brasch-Andersen, Katherine R. Bull, Holger Cario, Deirdre Cilliers, Valerio Conti, E. Graham Davies, Fatima Dhalla, Beatriz Diez Dacal, Yin Dong, James E. Dunford, Renzo Guerrini, Adrian L. Harris, Jane Hartley, Georg Hollander, Kassim Javaid, Maureen Kane, Deirdre Kelly, Dominic Kelly, Samantha J. L. Knight, Alexandra Y. Kreins, Erika M. Kvikstad, Craig B. Langman, Tracy Lester, Kate E. Lines, Simon R. Lord, Xin Lu, Sahar Mansour, Adnan Manzur, Reza Maroofian, Brian Marsden, Joanne Mason, Simon J. McGowan, Davide Mei, Hana Mlcochova, Yoshiko Murakami, Andrea H. Németh, Steven Okoli, Elizabeth Ormondroyd, Lilian Bomme Ousager, Jacqueline Palace, Smita Y. Patel, Melissa M. Pentony, Chris Pugh, Aboulfazl Rad, Archana Ramesh, Simone G. Riva, Irene Roberts, Noémi Roy, Outi Salminen, Kyleen D. Schilling, Caroline Scott, Arjune Sen, Conrad Smith, Mark Stevenson, Rajesh V. Thakker, Stephen R. F. Twigg, Holm H. Uhlig, Richard van Wijk, Barbara Vona, Steven Wall, Jing Wang, Hugh Watkins, Jaroslav Zak, Anna H. Schuh, Usha Kini, Andrew O. M. Wilkie, Niko Popitsch, Jenny C. Taylor

**Affiliations:** 1 Wellcome Centre for Human Genetics, University of Oxford, Old Road Campus, Roosevelt Drive, Oxford, OX3 7BN UK; 2 NIHR Oxford Biomedical Research Centre, John Radcliffe Hospital, Oxford University Hospitals NHS Foundation Trust, Oxford, OX3 9DU UK; 3 Human Technopole, Viale Rita Levi Montalcini 1, 20157 Milan, Italy; 4 Oxford Genetics Laboratories, Oxford University Hospitals NHS Foundation Trust, Churchill Hospital, Old Road, Oxford, OX3 7LE UK; 5 MRC Weatherall Institute of Molecular Medicine, University of Oxford, John Radcliffe Hospital, Oxford, OX3 9DS UK; 6 University Medical Center Groningen, Groningen University, PO Box 72, 9700 AB Groningen, The Netherlands; 7 Department of Oncology, Oxford Molecular Diagnostics Centre, University of Oxford, Level 4, John Radcliffe Hospital, Headley Way, Oxford, OX3 9DU UK; 8 Neurosciences Department, UHCW NHS Trust, Clifford Bridge Road, Coventry, CV2 2DX UK; 9 Nuffield Department of Clinical Neurosciences, University of Oxford, Oxford, OX3 9DU UK; 10 Division of Evolution, Infection and Genomics, School of Biological Sciences, Faculty of Biology, Medicine and Health, University of Manchester, Manchester, UK; 11 Manchester Centre for Genomic Medicine, Saint Mary’s Hospital, Oxford Road, Manchester, M13 9WL UK; 12 Pediatric Hematology-Oncology Unit, Kaplan Medical Center, Rehovot, Israel; 13 Hematology Department, Hospitais da Universidade de Coimbra, Coimbra, Portugal; 14 Oxford Centre for Genomic Medicine, Oxford University Hospitals NHS Foundation Trust, Oxford, OX3 7LE UK; 15 Department of Clinical Genetics, Odense University Hospital and Department of Clinical Research, University of Southern Denmark, Odense, Denmark; 16 Nuffield Department of Medicine, University of Oxford, Oxford, OX3 7BN UK; 17 Department of Pediatrics and Adolescent Medicine, University Medical Center, Eythstrasse 24, 89075 Ulm, Germany; 18 Neuroscience Department, Meyer Children’s Hospital IRCCS, Viale Pieraccini 24, 50139 Florence, Italy; 19 Department of Immunology, Great Ormond Street Hospital for Children NHS Trust and UCL Great Ormond Street Institute of Child Health, Zayed Centre for Research, 2Nd Floor, 20C Guilford Street, London, WC1N 1DZ UK; 20 Department of Paediatrics, Institute of Developmental and Regenerative Medicine, IMS-Tetsuya Nakamura Building, Old Road Campus, Roosevelt Drive, Oxford, OX3 7TY UK; 21 Oxford NIHR Musculoskeletal BRC and Nuffield Department of Orthopaedics, Rheumatology and Musculoskeletal Sciences, Nuffield Orthopaedic Centre, Old Road, Oxford, OX3 7HE UK; 22 Department of Oncology, University of Oxford, Old Road Campus Research Building, Oxford, OX3 7DQ UK; 23 Liver Unit, Birmingham Women’s & Children’s Hospital and University of Birmingham, Steelhouse Lane, Birmingham, B4 6NH UK; 24 Department of Paediatrics, University of Oxford, Level 2, Children’s Hospital, John Radcliffe Hospital, Oxford, OX3 9DU UK; 25 Department of Pharmaceutical Sciences, School of Pharmacy, University of Maryland, Pharmacy Hall North, Room 731, 20 N. Pine Street, Baltimore, MD 21201 USA; 26 Children’s Hospital, OUH NHS Foundation Trust, NIHR Oxford BRC, Headley Way, Oxford, OX3 9DU UK; 27 Feinberg School of Medicine, Northwestern University, 211 E Chicago Avenue, Chicago, IL MS37 USA; 28 University of Oxford, Academic Endocrine Unit, OCDEM, Churchill Hospital, Oxford, OX3 7LJ UK; 29 Early Phase Clinical Trials Unit, Department of Oncology, University of Oxford, Cancer and Haematology Centre, Level 2 Administration Area, Churchill Hospital, Oxford, OX3 7LJ UK; 30 Nuffield Department of Clinical Medicine, Ludwig Institute for Cancer Research, University of Oxford, Old Road Campus Research Building, Oxford, OX3 7DQ UK; 31 St George’s University Hospitals NHS Foundation Trust, Blackshore Road, Tooting, London, SW17 0QT UK; 32 MRC Centre for Neuromuscular Diseases, National Hospital for Neurology and Neurosurgery, Queen Square, London, WC1N 3BG UK; 33 Department of Neuromuscular Diseases, UCL Queen Square Institute of Neurology and The National Hospital for Neurology and Neurosurgery, London, WC1N 3BG UK; 34 Nuffield Department of Medicine, Kennedy Institute, University of Oxford, Oxford, OX3 7BN UK; 35 Yourgene Health Headquarters, Skelton House, Lloyd Street North, Manchester Science Park, Manchester, M15 6SH UK; 36 Research Institute for Microbial Diseases, Osaka University, 3-1 Yamadaoka, Suita, Osaka 565-0871 Japan; 37 Imperial College NHS Trust, Department of Haematology, Hammersmith Hospital, Du Cane Road, London, W12 0HS UK; 38 University of Oxford, Level 6 West Wing, Oxford, OX3 9DU JR UK; 39 Clinical Immunology, John Radcliffe Hospital, Level 4A, Oxford, OX3 9DU UK; 40 Department of Otolaryngology–Head & Neck Surgery, Tübingen Hearing Research Centre, Eberhard Karls University, Elfriede-Aulhorn-Str. 5, 72076 Tübingen, Germany; 41 Department of Haematology, Oxford University Hospitals NHS Foundation Trust, Level 4, Haematology, John Radcliffe Hospital, Oxford, OX3 9DU UK; 42 Ann & Robert H. Lurie Children’s Hospital of Chicago, 225 E Chicago Avenue, Chicago, IL 60611 USA; 43 Translational Gastroenterology Unit, John Radcliffe Hospital, Oxford, OX3 9DU UK; 44 UMC Utrecht, Heidelberglaan 100, 3584 CX Utrecht, The Netherlands; 45 Institute of Human Genetics, University Medical Center Göttingen, Heinrich-Düker-Weg 12, 37073 Göttingen, Germany; 46 Institute for Auditory Neuroscience and InnerEarLab, University Medical Center Göttingen, Robert-Koch-Str. 40, 37075 Göttingen, Germany; 47 Oxford Craniofacial Unit, John Radcliffe Hospital, Level LG1, West Wing, Oxford, OX3 9DU UK; 48 Department of Immunology and Microbiology, The Scripps Research Institute, 10550 North Torrey Pines Road, La Jolla, CA 92037 USA; 49 Department of Biochemistry and Cell Biology, Max Perutz Labs, University of Vienna, Vienna BioCenter(VBC), Dr.-Bohr-Gasse 9, 1030 Vienna, Austria

**Keywords:** Genome sequencing, Rare diseases, Structural variant, Splice site variant, Non-coding, Diagnostic yield, Clinical impact, Bioinformatics pipeline development, Pipeline optimisation

## Abstract

**Background:**

Whole genome sequencing is increasingly being used for the diagnosis of patients with rare diseases. However, the diagnostic yields of many studies, particularly those conducted in a healthcare setting, are often disappointingly low, at 25–30%. This is in part because although entire genomes are sequenced, analysis is often confined to in silico gene panels or coding regions of the genome.

**Methods:**

We undertook WGS on a cohort of 122 unrelated rare disease patients and their relatives (300 genomes) who had been pre-screened by gene panels or arrays. Patients were recruited from a broad spectrum of clinical specialties. We applied a bioinformatics pipeline that would allow comprehensive analysis of all variant types. We combined established bioinformatics tools for phenotypic and genomic analysis with our novel algorithms (SVRare, ALTSPLICE and GREEN-DB) to detect and annotate structural, splice site and non-coding variants.

**Results:**

Our diagnostic yield was 43/122 cases (35%), although 47/122 cases (39%) were considered solved when considering novel candidate genes with supporting functional data into account. Structural, splice site and deep intronic variants contributed to 20/47 (43%) of our solved cases. Five genes that are novel, or were novel at the time of discovery, were identified, whilst a further three genes are putative novel disease genes with evidence of causality. We identified variants of uncertain significance in a further fourteen candidate genes. The phenotypic spectrum associated with *RMND1* was expanded to include polymicrogyria. Two patients with secondary findings in *FBN1* and *KCNQ1* were confirmed to have previously unidentified Marfan and long QT syndromes, respectively, and were referred for further clinical interventions. Clinical diagnoses were changed in six patients and treatment adjustments made for eight individuals, which for five patients was considered life-saving.

**Conclusions:**

Genome sequencing is increasingly being considered as a first-line genetic test in routine clinical settings and can make a substantial contribution to rapidly identifying a causal aetiology for many patients, shortening their diagnostic odyssey. We have demonstrated that structural, splice site and intronic variants make a significant contribution to diagnostic yield and that comprehensive analysis of the entire genome is essential to maximise the value of clinical genome sequencing.

**Supplementary Information:**

The online version contains supplementary material available at 10.1186/s13073-023-01240-0.

## Background

Rare genetic diseases are defined as conditions affecting < 1 in 2000 people. Collectively, they are a common cause of morbidity affecting 6–8% of the population and already encompass over 7000 conditions, with more than 200 new conditions being described annually [1]. Our increased understanding of the genetic basis of rare diseases (RD) has had a profound impact on medicine and basic research; diagnostic pathways have been streamlined [2] and disease mechanisms informed by genetics are now common-place when previously they were rare. Knowledge of novel genetic variants and genes can inform new approaches to therapeutic interventions [3].

Central to these advances in genomic medicine has been the development of next-generation sequencing technologies. Initially used for targeted sequencing of known disease gene panels and exomes, the progressive reduction in costs has meant that sequencing patients’ entire genomes is now affordable as a first-line genetic test in a healthcare setting. Indeed, clinical whole genome sequencing (WGS) for RD patients is now being undertaken in several countries, including the UK (initially by Genomics England’s (GEL) 100,000 Genomes Project (100KGP) [4], and more recently as part of the NHS Genomic Medicine Service), Canada (through the Care4Rare programme [5]), the USA (through the Medical Genome Initiative [6]), Japan (as part of the Initiative on Rare and Undiagnosed Disease [7]), France (as highlighted in its Genomic Medicine 2025 plan [8]), Hong Kong [9], India (GUaRDIAN Consortium [10]) and Brazil [11], whilst the iHOPE programme (a philanthropic alliance funded by Illumina [12]) is providing under-served RD families across the world with WGS.

The key question now is how to further improve diagnostic yield as most individuals sequenced still remain without a genetic diagnosis. For example, the diagnostic yield of GEL’s pilot study of its first 2183 families (4660 genomes) is currently 25% [13], which is similar to that reported in other broad-spectrum clinical RD studies [14–16]. A major area for improvement is the interrogation of variant types and regions of the genome that would not be captured by the standard of care panel testing and arrays, or by whole exome sequencing (WES). Despite sequencing the entire genome, the clinical diagnostic analysis of WGS data has largely been restricted to identification of single nucleotide variants (SNVs) and small insertions/deletions (INDELs) in genes from pre-defined in silico panels or, at most, in the coding regions of the genome [17]. A systematic analysis of structural, non-coding and splice site variants has rarely been undertaken, yet it is precisely in these previously uncharted genome regions and variant types that the opportunity to improve the diagnostic yield of WGS lies.

Indeed, there is considerable evidence that these variant types contribute to the pathogenesis of RD. Structural variants (SVs), such as inversions, have been found to underpin a range of RDs [18, 19] several of which have only been identified by long read sequencing. Deep intronic variants, including splice site variants and those contributing to mRNA processing, have been reported over many years for a range of RD (reviewed by Vaz-Drago [20]) but have not been systematically investigated by clinical genome sequencing and the contribution of non-canonical splice site variants to RD is thought to be under-estimated [21]. The main reasons for the omission of these variant types from clinical WGS are the lack of appropriate tools and datasets for identifying and interpreting them, thereby differentiating the large numbers of true (but not pathogenic) and false (artefactual) variants from the pathogenic variants [22].

Building on a previous study, WGS500, in which we sequenced 500 genomes and identified factors critical to success in applying WGS for analysis of patient genomes [23], we sought to extend WGS to the clinical setting by establishing a clinical process for assessing referrals and by conducting sequencing within an accredited diagnostics laboratory, returning results within clinically relevant timeframes. Our OxClinWGS study, which we commenced before the 100KGP, included cancer and RD patients. Results from the cancer cohort, including the challenges of reporting results to inform timely use of targeted molecular therapies, have been described previously [24–28], as have the economic [29–31], legal [32] and ethical considerations [33, 34] of clinical WGS. In particular, we reported that costs of WGS are likely to be under-estimated if only sequencing consumables costs are considered and if analysis costs are not taken into account, and that aspirational costs per genome are only likely to be achieved in large-scale programmes [30].

This report focuses on the RD cohort. Patients who had not received a genetic diagnosis from standard of care clinical panel and/or array-based testing were recruited from a broad range of medical specialties, including neurological, musculoskeletal, immunological, haematological, cardiovascular and clinical genetics. Our aim was to undertake a comprehensive analysis of all variant types, including splice site, structural and non-coding variants, as we anticipated that these could have been missed by prior testing. We combined well-established bioinformatics tools with our own novel algorithms to aid with the identification and interpretation of these more challenging variant types. These include our SVRare tool to interrogate SVs [35], including copy number variants (CNVs), inversions and translocations; our novel algorithm for splice site variant detection and annotation, ALTSPLICE, and a custom GREEN-DB dataset [36] for annotation of non-coding variants. We report here the results and diagnostic yield from the RD cases in this OxClinWGS cohort.

## Methods

### Description of OxClinWGS cohort

The OxClinWGS cohort was prospectively collected as part of a Health Innovation Challenge Fund grant to undertake clinical-grade WGS for patients with a broad spectrum of RD and cancer. The RD cohort reported here comprised 300 genomes from 122 unrelated RD patients and their relatives (since proband/parent/parent trios were recruited where possible).

### Recruitment of patients

The majority of RD patients were recruited through an Oxford Genomic Medicine Multi-Disciplinary Team (GM-MDT), comprising a network of local clinicians/researchers and their collaborators, the process and experiences of which have been described previously [37, 38]. In brief, clinicians who proposed cases for WGS were asked to complete a short application form describing the patient’s condition, the likelihood of this being genetic, prior genetic testing carried out and availability of relevant samples from patient and family members. This was reviewed by the GM-MDT, and if approved, patients were consented and recruited. Only patients who had undergone prior standard of care genetic testing with high-resolution, microarray-based comparative genomic hybridisation (array CGH) and gene panel-based testing of known genes for their condition were recruited for WGS. Information given on the submission form was used to guide downstream analysis and extract Human Phenotype Ontology (HPO) terms. Additional HPO terms were obtained directly from clinicians if the initial form was not sufficiently detailed.

Patients were also referred from other UK centres, including Great Ormond Street Hospital (congenital thymic stromal defect cases), Birmingham Women’s and Children’s NHS Trust (cholestasis), University Hospital Coventry (encephalitis), Northampton General Hospital (Fine-Lubinsky syndrome) and overseas centres including University Hospital of Ulm, Germany (erythrocytosis), University Medical Centre, Utrecht, Netherlands (erythrocytosis), University Hospital of Coimbra, Portugal (erythrocytosis), Odense University Hospital Denmark (intellectual learning disability) and Meyer Children’s Hospital IRCCS, Florence, Italy (Aicardi syndrome). Clinicians at these centres were aware of the study through their collaborations with Oxford clinicians.

### Patient consent and clinical information

Fully informed consent was obtained from all participants by a genetic counsellor or medical specialist. The majority of participants were consented under the Molecular Genetic Analysis and Clinical Studies of Individuals and Families at Risk of Genetic Disease study (MGAC). Further details of the approved ethics protocols used are provided in the “Declarations” section.

The OxClinWGS study offered participants the option to receive feedback on pertinent and secondary findings. Secondary findings included those identified incidentally, as well as additional findings identified through screening of a pre-defined list of genes recommended by the American College of Medical Genetics (ACMG) [39]. A framework for careful return of these secondary findings was developed involving a re-consent process and, for cardiac conditions, the opportunity to follow up the findings with clinical investigations. The pathway we established for review and return of secondary findings has been described previously [34].

**Fig. 1:**
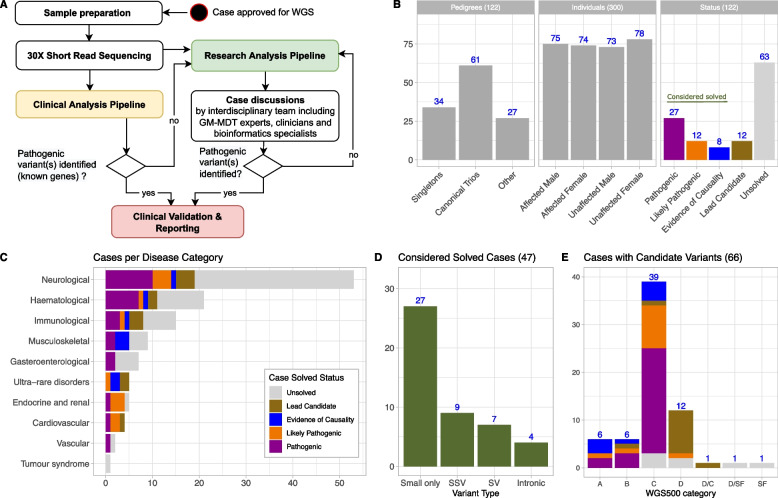
Overview of OxClinWGS study: workflow, clinical cases and results. **A** Case selection and referral was mainly done by the Oxford Genomic Medicine Multidisciplinary Team (GM-MDT); a detailed description of this process is provided elsewhere [37]. Selected samples were whole-genome sequenced and analysed by a clinical (yellow) as well as a research pipeline (green). Identified pathogenic candidate variants were validated and reported back to referring clinicians. Unsolved cases were iteratively investigated by a research pipeline incorporating the latest methods for in silico analysis of WGS data. Resulting novel disease candidate variants were regularly discussed by an interdisciplinary expert team and either rejected or forwarded to (functional) validation. **B** Core statistics of considered pedigrees (*n* = 122) and individuals (*n* = 300). Note that some families had more than one affected individual. Criteria for the shown classification of pathogenicity are discussed in the main text. **C** Considered disease categories, coloured by case status. **D** Variant types for considered solved cases (including pathogenic, likely pathogenic and cases with evidence of causality, see main text). Small only: all causative variants are SNVs or small INDELs, SSV: at least one causative variant is a splice site variant, SV: at least one causative variant is a large structural variant, Intronic: at least one causative variant is (deep) intronic. **E** Classification of cases using a previously introduced schema published in [23]: A: variant in novel gene for phenotype with additional (genetic) evidence; B: novel (mechanism) for phenotype; C: known gene for phenotype; D: variant in novel gene for phenotype, further genetic and functional validation studies in progress; SF: secondary finding. Note that two cases have candidate variants in two categories. A detailed description of the categories is provided in Additional file 3: Table S3. Abbreviations: SV, structural variant; SSV, splice site variant

A summary of the recruitment process and routes for feeding back results to clinicians is shown in Fig. 1A and Additional file 1: Fig. S1. Clinical case histories for selected patients are provided in Additional file 2.

### WGS and bioinformatic analysis

#### Sample collection

Blood samples (30 ml) were collected from all participants. DNA was extracted using the Gentra Puregene kit (Qiagen) or similar methodologies. DNA was also extracted from a skin biopsy of an affected haemangioma lesion (005Kli001) and from healthy or diseased bone tissue biopsies (065DSA001) for somatic variant analysis (see below).

#### Whole genome sequencing

WGS was conducted in the Oxford Molecular Diagnostics Centre, a laboratory accredited to ISO15189 standards. We initially validated the WGS workflow and detection of single nucleotide variants (SNVs) and SVs on a set of 10 cancer and 10 RD samples sequenced as part of the WGS500 project [23]. Ongoing validation of a range of variant types was undertaken as part of the regular NEQAS and ISO certification processes, to ensure concordance with other centres. This is important since the analytical and clinical sensitivity of NGS workflows can vary considerably [40].

Sequencing was carried out to a mean of 30 × coverage using a paired end sequencing protocol on either a HiSeq2500 or HiSeq4000 instrument (Illumina) following the manufacturer’s instructions.

Initially, all data were processed on Illumina BaseSpace using a case-by-case approach and analysed in the clinical genetics diagnostic laboratory using Variant Studio (Illumina) to investigate locally curated gene panels relevant for the specific disease. Analysis of variants outside known disease genes was beyond the remit of the clinical laboratory and was undertaken in a research setting. Any variants identified in the research setting were referred back to an accredited clinical laboratory for validation and reporting.

Unsolved cases referred for research analysis were initially explored using Ingenuity Variant Analysis (Qiagen) but at the end of the project, a single pipeline was designed and was used to re-analyse all samples in a consistent way and build a cohort representation of genetic variants. This automated pipeline was built on Nextflow [41] and performed read alignment to GRCh38, alignments QC, variant calling for small variants and SVs, runs of homozygosity (ROH) and repeat expansion detection [42]. Our pipeline combines algorithms and tools to interrogate a wide range of variant types, some of which have been developed by other groups, and some by us, but all of which are publicly available and could be applied in clinical or research settings and are not specific to a UK healthcare context.

Any sample with less than 75% bases covered at least 10 × across the genome, as well as samples with genetically inferred information discordant with data from the clinical report, and samples with an unexpectedly high rate of heterozygosity, indicating possible sample contamination, were removed from the subsequent cohort analysis. Coverage of samples for the retained cohort is shown in Additional file 1: Fig. S2. QC checks were also carried out to ensure that ancestry, relatedness and gender were as expected for known demographics of study participants (Additional file 1: Figs. S3-S5).

#### Variant identification and annotation

Our pipeline combined several different methods to detect all kinds of genetic variants across our WGS cohort as follows: (i) small variants were detected using DeepVariant v1.0.0 [43], merged across individuals using GLNexus v1.2.6 [44] and then normalised using bcftools norm; (ii) SVs and CNVs were detected using a combination of Lumpy v.0.2.13 and CNVnator v0.4.1 and then integrated in a cohort-wide representation using svtools v0.5.1 as described [45], and as a complementary approach, we also applied our novel algorithm, SVRare (see below) [35], for large structural variant discovery; (iii) repeat expansion variants were detected for a set of known expansions in 29 genes using ExpansionHunter v3.2.2 [46] and the corresponding variant catalogue; (iv) ROH were detected for each sample directly from the cohort VCF using bcftools roh based on genotype likelihoods.

The resulting cohort VCFs containing small variants and SVs were filtered based on quality metrics to obtain a clean representation of variants across the cohort and then annotated for gene level consequences using SnpEFF v4.3t [47], population allele frequency (AF) (gnomad v2 and 1000G phase3), impact prediction scores (CADD [48], DANN [49], REVEL [50], FATHMM [51], ncER [52], ReMM [53], spliceAI [54], MaxEntScan [55]) and known disease-causing variants (from ClinVar [56], dbVAR [57] and DECIPHER [58]). Small variants were annotated using vcfanno v0.3.1 [59] while a custom python script was used for SVs. Finally, possible consequences for non-coding variants were annotated using GREEN-VARAN v1.0 and GREEN-DB v.2.5 information (see below) [36, 60].

#### Gene-level annotation

For each case, a ranked list of genes potentially relevant for the family phenotype was calculated based on the respective HPO profile using GADO v1.0.1 [61] and genes above the 90th percentile in the GADO ranking were selected as best candidates. Pedigrees were further analysed with Exomiser v12.1.0 [62] using data release 2102 and a portable version of this HPO prioritisation pipeline is publicly available [63]. Candidate genes were further annotated using pLI values from gnomAD v2.1.1, the GDI score (human damage index) [64] and the RVIS (Residual Variation Intolerance Score) value [65] based on ExAC v2.

#### Analysis of cohort dataset

Annotated cohort-wide VCF files containing small and large (SV) variants were filtered and pre-processed by a custom python script (cohort_varan) that generates an integrated view of segregating variants with their annotations. We developed Variant Explorer (VE), a graphic user interface to allow disease experts to interactively explore the results generated for each pedigree and apply custom filters based on variant segregation and the rich set of variant annotations added by our pipeline. VE was developed in R using Shiny and shinydashboard to implement the graphical user interface. The flexible filtering system can apply different filtering strategies to specific variant groups defined by variant consequences and perform complex segregation filtering, such as selecting compound heterozygotes involving variants with a specific effect. Further filtering can be applied based on ROH regions, additional regions of interest provided in a BED file or gene-based annotations like GADO score. Cohort_varan and VE codes are available on GitHub [66, 67].

To conduct cohort-wide analyses, we integrated the various output files from the bioinformatics pipeline (e.g. annotated VCFs, GADO and Exomiser result tables, etc.) as well as several additional annotation resources (e.g. PED files and gene annotations) into a partitioned parquet database using custom python scripts based on Apache arrow, pysam, numpy and pandas libraries. The resulting parquet database was then loaded into an Apache spark cluster and queried via RStudio/sparklyr.

A detailed description of bioinformatic methods is reported in Additional file 1, including variant annotation software tools and versions used and the categories applied to systematically describe the impact of variants (cited in Additional file 3: Tables S1 and S2, respectively). We also used a classification system to describe whether genes resulting from the analysis were novel, novel for phenotype, known or lead candidates (Additional file 3: Table S3), as previously reported for the WGS500 study [23].

#### Discovery and filtering of structural variants with SVRare

We applied SVRare [35] for large variant discovery. Briefly, Manta (v1.6.0) [68] and Canvas [69] were used to call SVs. The resulting data were imported, together with the svtools results, into a sqlite3 database. Events were merged using a similarity threshold of 0.5 and were annotated using gnomAD SV (v2.0) [70], dbVAR [57] and DECIPHER [58]. SVs were prioritised and visualised using SVRare-js [71]. SVRare had previously been validated using diagnostic grade SVs in 4,313 families from the 100KGP pilot study [35] and had identified inversions in 7/5,222 families from the musculoskeletal (MSK) domain of 100KGP, four of which have been clinically reported [17, 72].

#### Prediction of splice-site variants

In addition to the well-established splicing prediction algorithms, SpliceAI [54] and MaxEntScan [55], we used a novel algorithm developed internally, ALTSPLICE [73], to investigate potential splice sites. ALTSPLICE uses the underlying DNA sequence to predict the impact of mutations on exon inclusion rates in expressed gene transcripts in two stages. First, the location and usage frequency of splice donor and acceptor sites are predicted, using a multilayer convolutional neural network with 16,384 bases as input, with the predicted frequencies and their uncertainty represented as a Beta distribution. The true usage frequencies are estimated from GTEx read junction data and also represented as a Beta distribution. A loss function compares these distributions and accounts for model mis-specification at particular *loci* and the existence of mis-mapped reads. Second, predicted splice junctions are considered within known transcripts and exon reading frames. The resulting transcripts (“primary transcripts”) and their implied frameshifts are used to predict whether Nonsense-Mediated Decay is triggered, resulting in transcript expression levels relative to the full set of primary transcripts. To assess whether a mutation affects transcript expression, the same procedure is run for the computationally mutated sequence and predicted relative expression levels compared on the transcript level. ALTSPLICE classifies sites into non-splicing, alternatively spliced and constitutively spliced sites, to quantify the degree of alternative splicing. This sets it apart from existing approaches that aim for a binary classification such as SpliceAI. In addition, the ALTSPLICE model is able to handle larger sequences (> 16 kb) than previous approaches such as SpliceAI which are limited to 10 kb. It can be used to investigate the effect of SNVs on alternative splicing [73]. Since ALTSPLICE was developed after our initial analysis of the cohort, it was not integrated into the main bioinformatics pipeline, but applied to trios and cases which had a suggestive SpliceAI score, for additional annotation. Further details of ALTSPLICE are available on GitHub [73].

#### Non-coding variant annotation

We used GREEN-DB v2.5 and GREEN-VARAN v.1.0 workflows [36] to annotate and prioritise non-coding variants. The GREEN-DB is an extensive collection containing approximately 2.5 M regulatory regions and information on the region function (i.e. enhancer, promoter, silencer), their tissue of activity and controlled gene(s). A constraint metric representing tolerance to mutation for each regulatory region is also provided. Taking this information into account, GREEN-VARAN annotates non-coding variants with potentially impacted genes. The tool also evaluates values from three impact prediction scores, namely ReMM v0.3.1 [53], FATHMM-MKL [51] and ncER [52], together with the variant allele frequency, to compute an impact level from 1 to 4 that summarises the likelihood of the variant affecting the gene expression.

For additional evaluation of non-coding variants, in particular predicting chromatin features from DNA sequence, we used deepHaem, a convolutional neural network whose basic architecture is built on the principles described in DeepSEA [74] and Basset [75]. It comprises multiple layers of convolutional filters followed by ReLU and max pooling and a fully connected layer in the end.

For human chromatin feature compendiums, the ENCODE [76] and Roadmap [77] chromatin data used in DeepSEA were used, supplemented with additional erythroid lineage data. Details of the datasets and their processing can be found in [78]. DeepHaem was trained to predict chromatin features from 1 kb of DNA sequence, using the compendium of 936 datasets described above. Five convolutional layers (hidden units 300, 600, 600, 900, 900; filter widths 8, 8, 8, 4, 4, 4) with ReLU activation, maximum pooling (widths 4, 5, 5, 5, 2) and dropout (rate 0.2) were used followed by a fully connected layer with sigmoid activation to output individual probabilities for each chromatin feature class (multi-label classification). The network parameters were trained by minimising the sum of the binary cross entropies using the ADAM optimiser (epsilon 0.1) in batches of 100. Batch size, dropout rate, learning rate and filter size were optimised by grid search. The potential consequences of a subset of non-coding variants were calculated by subtracting the deepHaem predicted chromatin class scores of the variant sequence from the reference sequence (hg38) and ranking all classifiers. Further details are available on GitHub [79].

For 010Kap001, the HOXB13 binding motif (MA0901.1) was retrieved from the JASPAR database [80]. HOXB13 was used as an example but the motif is virtually indistinguishable from other HOX transcription family (TF) members, e.g. HOXA13 (MA0650.1) or HOXD13 (MA0909.1).

#### Identification of somatic mosaic variants

For cases where germline variants were not identified, and the phenotype suggested that somatic mosaic variants should be considered (e.g. overgrowth syndromes), we undertook additional targeted sequencing or WGS. For 005Kli001, DNA was extracted from a skin biopsy of an affected haemangioma lesion and targeted next generation sequencing at high coverage was carried out using the AmpliSeq Cancer Panel on the Ion Torrent sequencing platform. For 065DSA001, DNA was extracted from cell cultures derived from healthy or diseased bone tissue biopsies and WGS was undertaken as described above, except that coverage of healthy or diseased bone was at 30 × and 120 × mean coverage, respectively. Sequencing, alignment and QC were performed as described above and Mutect2 v4.2.0.0 was used to identify somatic variants in each dataset.

#### Protein informatics

The potential impact of coding variants identified from WGS on protein structure was assessed using our custom protein informatics algorithms: MichelaNGLo [81] for visualisation of the location of the variant and Venus for predicting its effects [82].

### Validation of structural variants

SVs were validated using Nanopore sequencing (as described [83]), optical genome mapping (Saphyr, Bionano; see Additional file 1, Supplementary methods), microarrays, targeted resequencing or with a commercial multiplex ligation-dependent probe amplification assay (MLPA) kit (P189-C2, MRC Holland).

### Validation of splice site variants

Splice site variants were confirmed using RT-PCR analysis, RNA Seq or minigene approaches customised to the variant and region in question (Additional file 1, Supplementary methods) using a range of different vectors such as pBI-CMV2 (Clontech # 631631), RHCglo (Addgene plasmid #80169) [84] and pSPL3 (Invitrogen).

#### RNA sequencing and analysis

Whole blood (2.5 ml) was collected into PAXgene tubes (Qiagen). Total RNA was extracted using the PAXgene Blood RNA kit according to the manufacturer’s instructions. RNA purity was assessed using the 260/280 ratio detected on Thermo Scientific™ NanoDrop 2000. All samples had a ratio of 2.0–2.12 confirming the purity of the RNA. RNA quantity was assessed using Qubit 2.0. RNA quality was then assessed on an Agilent Bioanalyser 2100 using RNA 6000 Pico Total RNA Kit (Agilent Technologies). The RNA integrity number (RIN) score ranged between 7.5 and 9, indicating good integrity of the RNA.

The library preparation was performed using TruSeq Stranded Total RNA Library Prep Kit with Ribo-Zero (Illumina) according to the manufacturer’s instructions and sequencing was performed on the HiSeq4000 system (75 bp paired-end reads) to a minimum depth of 50 M reads.

Raw reads were subjected to quality control using fastQC then aligned to the GRCh38 genome using STAR2.5.2a [85] with Ensembl transcriptome annotations version 87. The BAM file was processed using samtools v1.3 and analysed using spladder v2.2.1 [86].

### Functional validation

Additional functional assays were carried out in order to confirm or refute the pathogenicity of variants and are referenced below or described in Additional file 1, Supplementary methods.

### Classifications of pathogenicity

We used the ACMG guidelines to classify variants as pathogenic, likely pathogenic, likely benign, benign or variants of uncertain significance [87]. We also used the UK Association for Clinical Genomic Science Practice Guidelines [88] to inform further classification of variants of unknown significance (VUS) as hot, warm, tepid and cool.

In addition, we identified a group of VUS in both known and novel disease genes which had compelling evidence of causality because (i) clinicians considered the VUS as being causative for their patient, and changed the diagnosis accordingly; (ii) a patient had been treated based on the molecular defect identified by WGS; (iii) the case had been published in peer-reviewed journals; (iv) additional patients with overlapping phenotypes from GeneMatcher, 100KGP or other sources had been identified; and/or (v) there was additional functional support for the VUS.

We then defined our diagnostic yield as the cases with ACMG pathogenic or likely pathogenic classifications and VUS in *known* disease genes with evidence of causality. We have additionally defined a group of cases which are ‘considered solved’ which includes the cases counted in the diagnostic yield and the VUS in *novel* genes which have evidence of causality.

The pathogenicity of all cases was assessed by qualified clinical scientists of the Regional Genetics Laboratories at the Oxford University Hospitals Foundation Trust and reviewed by the referring clinicians for the relevant patients.

## Results

### Overview of cohort results

The OxClinWGS RD cohort comprised a total of 300 genomes from 122 families. One hundred forty-eight male and 152 female participants were recruited, the majority of whom were of European White ancestry, although African, Asian and American families were also included (Additional file 1: Fig. S3), reflecting the population from which the patients were primarily recruited. Overall cohort statistics, including details of family size, gender, disease categories and solved status of individual cases recruited, are shown in Fig. 1B, C, Additional file 3: Tables S4-S6 and Additional file 1: Figs. S6 and S7. The results of WGS for all patients in the cohort, including causative genes and variants (where solved) and associated phenotypes are provided in Additional file 3: Tables S6 and S7, which also include references for some individual cases which have been published previously. More detailed clinical case histories for selected patients are provided in the Additional file 2. Variants identified in this study have been uploaded to ClinVar [89].

Our diagnostic yield in this RD cohort was 43/122 cases (35%). These were cases which had variants with ACMG classifications of pathogenic/likely pathogenic (39/43) or were variants with evidence of causality in *known* disease genes (4/43) which were clinically accepted and returned, informing diagnosis or treatment of these patients. Across the cohort, we considered 39% of our cases to be solved (47/122) since an additional four cases had variants in *novel* disease genes that had compelling evidence of causality from additional families with matching phenotypes, or functional data (Fig. 1B, Additional file 3: Tables S7 and S8). A further 12/122 (10%) cases had a variant of uncertain significance in a lead candidate identified from genetic analysis. Two cases with clinically actionable secondary findings were also identified. An overview of cases considered solved by variant type is shown in Fig. 1D and WGS500 classification of genes (see the Methods) is shown in Fig. 1E. Further details of inheritance pattern, de novo status and result class are summarised in Additional file 1: Fig. S8.

Across the cohort, we identified eight novel disease genes. Three of these have been confirmed and previously published as part of collaborative studies; a de novo p.(Gln735*) mutation in *POLR2A* in a patient with a novel neurodevelopmental syndrome with profound infantile-onset hypotonia [90]; a de novo p.(Tyr1224fs) mutation in *KMT2E* in a patient with a neurodevelopmental syndrome and epilepsy [91] and biallelic variants (p.(Gly79fs) and c.764 + 5G > A) in *MCM10* causing telomere shortening and giving rise to immune dysfunction and cardiomyopathy [92]. Two genes, *DOCK7* and *SAMD9L*, were novel at the time of discovery and we have evidence of causality for a further three novel disease genes (*DHRS3*, *FOXD3*, *HDLBP).* Variants in other lead candidates are also being investigated in functional studies. Additionally, one gene, *RMND1*, is novel for the phenotype of polymicrogyria whilst *BMP4* is a putative novel gene for Kapur-Toriello syndrome which, if confirmed, would extend the phenotypic range of this gene from its current association with microphthalmia and clefting syndrome.

**Fig. 2:**
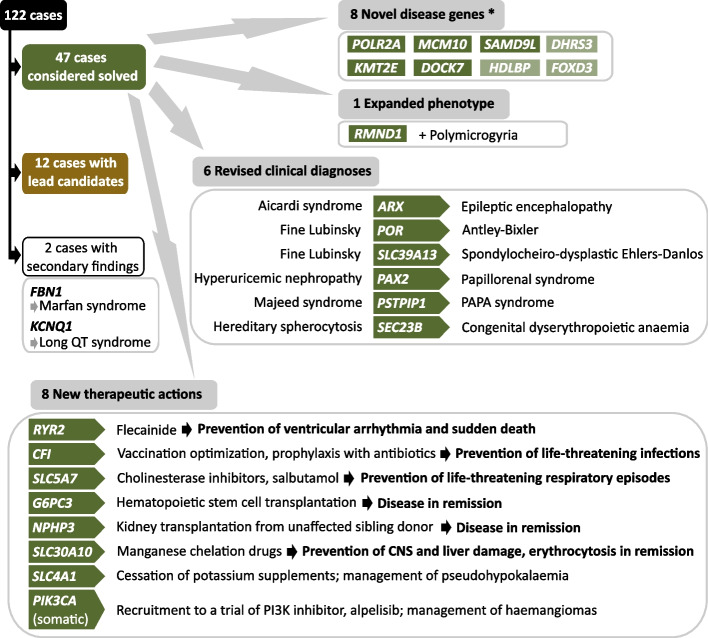
Overview of the OxClinWGS Study: genetic and clinical results. The OxClinWGS RD cohort included 122 cases, of which 47 were considered solved and a further 12 cases had variants of uncertain significance in lead candidates identified. Two cases had secondary findings. Eight novel disease genes have been identified to date, five of which are confirmed disease genes and three of which have evidence of causality. The asterisk denotes that this group includes novel and putative novel genes. The phenotype for one gene was expanded. Revised clinical diagnoses were provided for six patients, whilst for eight patients, the findings led to changes in their clinical management. Colours denote cases with genes that are considered solved (green), have evidence of causality (light green) or are variants of uncertain significance in lead candidates (brown). Abbreviations: PAPA syndrome (pyogenic sterile arthritis, pyoderma gangrenosum, and acne); CNS (central nervous system)

A summary of the outcomes of the project in terms of cases solved and novel candidate disease genes is shown in Fig. 2.

### Overview of variant types and HPO information

Our pipeline investigated all variant types, including SNVs, INDELs and SVs. The numbers of all variants by category, their minor allele frequency (MAF), size distribution and predicted impact are shown in Additional file 1: Figs. S9-S12 inclusive. For each variant class, we investigated variants arising de novo per pedigree (Additional file 1: Fig. S13)*.* Fourteen pathogenic/likely pathogenic (ACMG classification) de novo variants were identified, including one secondary finding in *FBN1* (see below). HPO terms were integrated into the analysis and helped to prioritise potential disease genes linked to the annotated patient phenotypes. On average, 4.7 HPO terms were recorded per pedigree (range 1–24) with ‘seizures’ being most common (Additional file 1: Fig. S14). We generally observed that solved cases clustered with higher numbers of HPO terms. Heatmap analysis of the HPO profiles (Additional file 1: Fig. S15) demonstrated overlap between different disease groups. For example, our ultra-rare disorder cases aligned with neurological and MSK groups, which can be explained by the fact that this category contains Fine-Lubinsky and Kapur-Toriello syndrome patients, which have some shared features with the craniosynostosis patients in the MSK group. In addition, clinical characteristics shared between vascular, haematological and immunological patients are also reflected in the heatmap.

We used several established and recently published deleteriousness scores to prioritise/filter causative variants and the respective value distributions, including our candidate pathogenic variants, as shown in Additional file 1: Fig. S16. No single score was able to perfectly separate true from false positives and hard filtering, for example, for a CADD PhredScore > 20 as suggested in some studies, would have caused us to miss 14/55 (25%) of our candidate SNVs.

Whilst the majority of our cases were explained by protein-coding SNVs, it is noteworthy that SVs, splice site and deep intronic variants, which have been hitherto under-explored in WGS studies, collectively contributed to 20/47 (43%) of our solved cases. These are described in more detail below.

### Structural variants

Structural candidate variants accounted for 4/43 (9%) of our diagnostic yield and 7/47 (15%) of the cases we consider solved (Table 1). Three SVs have led to the identification of two putative novel disease genes. The first, a homozygous 3.9 kb deletion encompassing the promoter and 5′-UTR of *DHRS3*, was identified in two siblings from a consanguineous Pakistani family with craniosynostosis. A deletion in this gene, which encodes dehydrogenase/reductase-3, would be expected to lead to an increase in the plasma level of the morphogen all-trans retinoic acid, which was confirmed by liquid chromatography multi-stage tandem mass spectrometry.

**Table 1:**
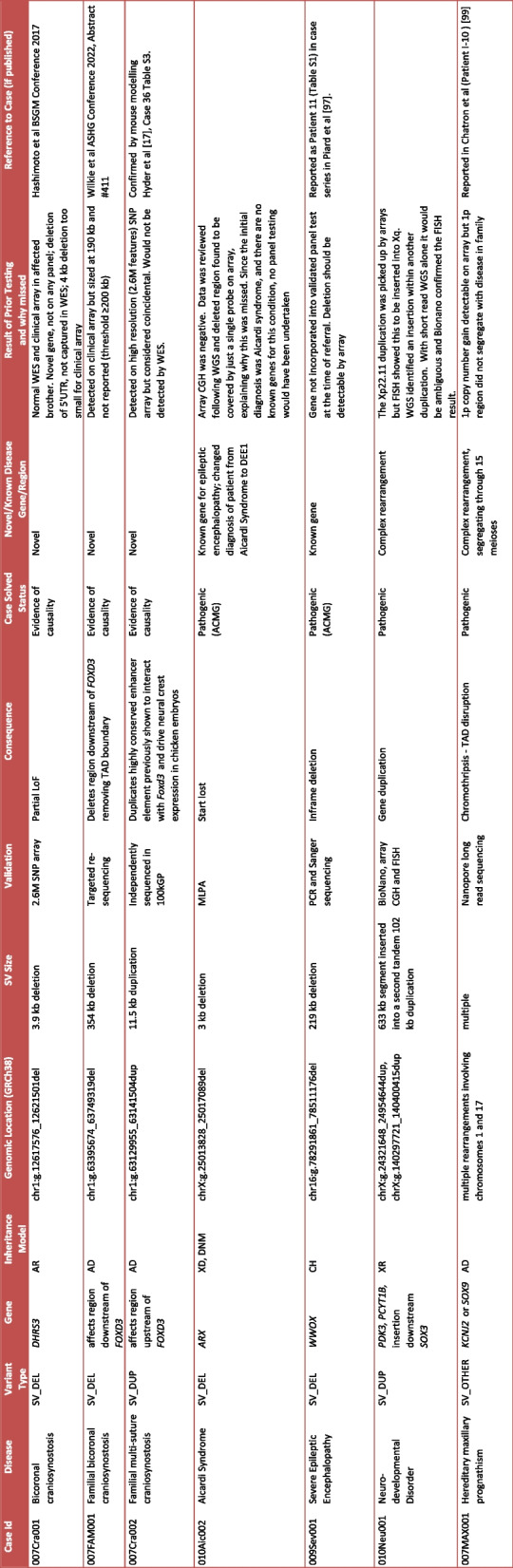
Structural variants found in the OxClinWGS cohort

Seven SVs were identified, three of which implicate two novel disease genes (*DHRS3, FOXD3*). One SV led to a change in diagnosis from Aicardi to early onset encephalopathy (DEE1) and one is in a known gene (*WWOX*). Two SVs represent complex rearrangements. All seven of the SVs are pathogenic or have evidence of causality and the cases are therefore considered solved. Details of prior testing and whether variants could have been detected by standard of care testing (arrays, panels or exomes) are included

*Abbreviations*: *SV* structural variant, *AR* autosomal recessive, *AD* autosomal dominant, *CH* compound heterozygous, *XD* X-linked dominant, *XR* X-linked recessive, *DNM* *de novo* mutation, *UTR* untranslated region, *WES* whole exome sequence, *FISH* fluorescent *in situ *hybridisation, *CGH* comparative genomic hybridisation, *TAD* topologically associated domain, *LoF* loss of function, *DEE1* developmental and epileptic encephalopathy type 1

Two further craniosynostosis families were found to have heterozygous SVs flanking a second, novel RD gene, *FOXD3*, both segregating with disease in their respective families. *FOXD3* encodes a pioneer winged-helix transcription factor (TF) critical for early embryonic development [93] and is therefore a good candidate for craniosynostosis. One of these families, with bicoronal craniosynostosis, harboured a 354 kb deletion downstream of *FOXD3*, removing a topologically associating domain (TAD) boundary*.* Another family, with multi-sutural craniosynostosis, had an 11.5 kb duplication upstream of *FOXD3* which duplicates a highly conserved enhancer element previously shown to interact with *Foxd3* and drive neural crest expression in chick embryos [94, 95]. This SV has been confirmed by modelling in mice, which also develop craniosynostosis [17].

**Fig. 3:**
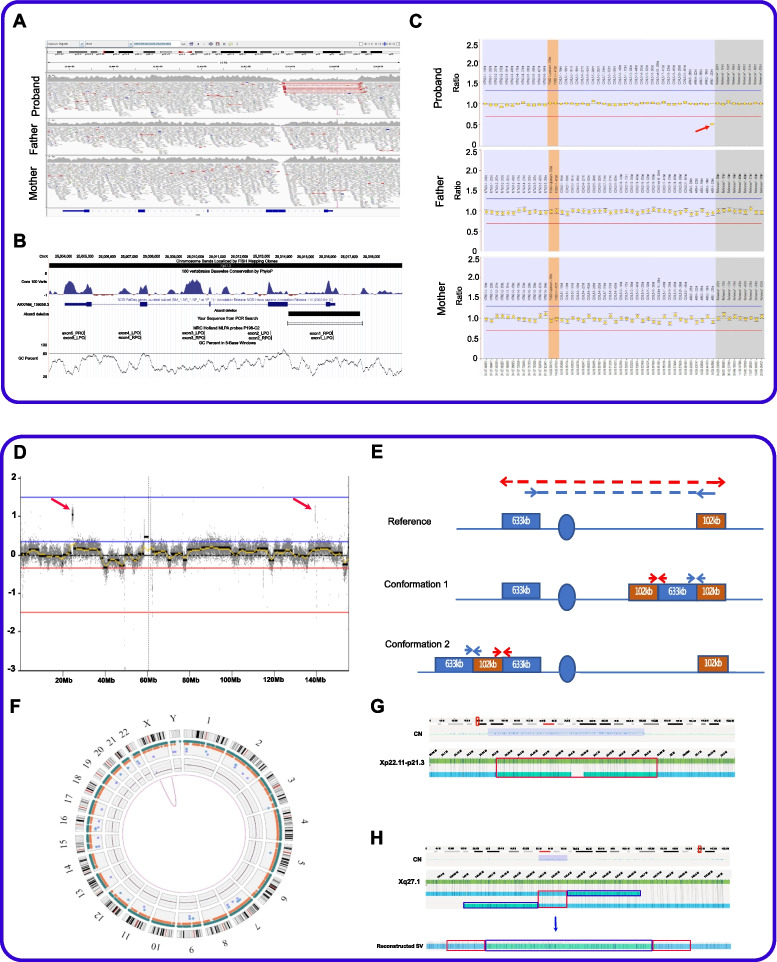
Validation data for two patients with X-linked structural variants in OxClinWGS study. **A–C** Patient with *ARX* deletion: **A** screenshot showing 125 bp read alignments supporting a de novo deletion of *ARX* exon 1. Region shown is chrX:25,003,000–25,019,000 (GRCh38). Visualisation is using IGV v2.11.2, with squished and “view as pairs” options. Alignments are coloured by insert size and transcript shown is NM_139058.3. **B** UCSC genome browser session showing the position of the deletion in relation to the PCR primers and MLPA probes that were used for validation. Also shown is the GC content which rises to > 80% near the distal breakpoint where the coverage drops also in parental genomes. An interactive version is available at https://genome.ucsc.edu/s/AlistairP/ARX_deletion_v6. **C** The 3 kb deletion was confirmed by the MLPA validation data visualised using coffalyser and shows a drop in signal only for the proband for the exon 1 probe (red arrow). The grey boxes are reference probes and the orange boxes highlight the 95% confidence range of the reference samples used. **D–H** Complex rearrangement in patient with X-linked neurodevelopmental disorder. **D** Read count information from short-read sequencing normalised by ngCGH software (https://github.com/seandavi/ngCGH) showing two X chromosome duplications (red arrows). **E** Split Illumina read-pairs suggest the two duplications are inter-linked. However, two possible configurations can explain the split read pattern. **F** Circos plot highlights the only SV identified by the Bionano pipeline above the threshold for SV detection. **G** Genome browser view of the optical maps robustly detects the ~ 600 kb duplication of Xp22.11p21.3 being inserted into Xq27.1, which is present in the carrier mother and both affected male siblings using the Bionano pipeline excluding Complex Multi-Path Regions (CMPR). The red box highlights the duplication inserted into Xq27.1. **H** The Bionano pipeline without masking CMPR detects ~ 102 kb tandem duplication (red boxes) flanking either side of the 600 kb insertion from Xp22.11-Xp21.3 (blue box), therefore, supporting conformation 1 as suggested in** E**

A fourth SV led to a change in clinical diagnosis for a female patient referred with Aicardi syndrome, a rare congenital malformation syndrome found almost exclusively in females and characterised by agenesis of the corpus callosum, seizures and chorioretinal lacunae. No genes have been identified to cause this syndrome. A ~ 3 kb de novo deletion on the X chromosome identified in our patient removes the first exon of *ARX*. Validation of this deletion by PCR and Sanger sequencing was confounded by the nearby repeats and high GC content of the region but it was instead confirmed by MLPA (Fig. 3A–C). Variants in *ARX* have been associated with several X-linked intellectual disability (XLID) syndromes, including XL lissencephaly, developmental and epileptic encephalopathy type 1 (DEE1) and Partington syndrome [96], reflecting the central role of this member of the homeobox gene family of TFs in controlling the formation of many brain structures during early embryonic development. As a result of our WGS finding, the clinical characteristics of this patient were reviewed, and since she had developmental and epileptic encephalopathy and corpus callosum agenesis, but not the ophthalmic features typical of Aicardi syndrome, her clinical diagnosis was changed to DEE1 (OMIM #308350).

A fifth SV led to an in-frame 219 kb deletion of exons 6–8 of *WWOX*, leading to loss of 180 amino acids including the mitochondrial targeting sequence. This was in *trans* with a c.705dup p.(His236fs) variant in this known epilepsy gene and provided a diagnosis for a patient with severe epilepsy. These compound heterozygous variants were previously reported as part of a case series expanding the phenotypic spectrum associated with this gene [97].

Two further SVs represent more complex rearrangements. A large 633 kb duplication of Xp22.11-Xp21.3 had been identified by prior clinical array testing in two brothers with a severe neurodevelopmental syndrome and hypotonia. Short-read WGS data allowed us to confirm the precise breakpoints of this rearrangement, in addition to identifying a second 102 kb duplication of Xq27.1 (Fig. 3D). The larger duplication encompasses *PDK3*, *PCYT1B* and *POLR1A*, while the smaller one did not contain any annotated genes. Although split read-pairs indicated that the two duplications were interlinked, short-read data alone could not resolve which of the two possible configurations was correct (Fig. 3E). However, FISH data combined with optical-mapping, an orthogonal technique (Fig. 3F–H), suggest that the 633 kb segment is inserted within the 102 kb tandem duplication, ~ 200 kb downstream of *SOX3*. Genomic insertions downstream of *SOX3* have been reported to cause a number of variable conditions that include hypoparathyroidism and laryngeal abductor paralysis [23, 98]. Therefore, we postulate a similar positional effect here, involving long-distance regulatory mechanisms.

The second complex rearrangement was found in a patient with hereditary maxillary prognathism. This patient had five segments of chromosome 1 inserted into chromosome 17q24.3, which is hypothesised to disrupt a TAD close to *KCNJ2/SOX9*. The rearrangement was confirmed by nanopore long-read genome sequencing and has been classified as an example of chromoanasynthesis [99] revealing a new mechanism for this rare craniofacial phenotype.

Although in principle, four of these SVs (*WWOX*, two *FOXD3* and *DHRS3* variants) could have been detected by arrays, they were not picked up by standard of care testing prior to WGS referral, because they were inadequately covered by probes, did not meet the thresholds for clinical laboratory reporting or were in novel genes; therefore, their significance was not appreciated (Table 1). We note that for two complex SVs, detection by array only would leave their full complexity under-appreciated and indeed for one of these, WGS analysis to characterise the precise insertion site was the reason for recruitment, as the larger of the duplicated segments had already been identified.

All SVs were validated by independent methods, including PCR and Sanger sequencing, MLPA, SNP arrays, nanopore long range sequencing and BioNano (Table 1) and the range of the methods required highlights the challenges of doing this at scale in a routine clinical setting.

### Splice site and deep intronic variants

We used three different splicing algorithms to inform our analysis of splice site variants; SpliceAI, MaxEntScan and our novel algorithm, ALTSPLICE. We first validated ALTSPLICE by comparing its performance with that of SpliceAI, using a previously published, manually curated set of clinical splice-altering and control SNVs [100]. The scores from ALTSPLICE and SpliceAI are shown in Additional file 3: Table S9. The area under the precision recall curve was found to be 96.8% for ALTSPLICE and 96.4% for SpliceAI (Additional file 1: Fig. S17), validating the ALTSPLICE algorithm and demonstrating that the performance of the two is similar overall, even though they are independently constructed and trained.

We identified sixteen splice site or deep intronic variants (of which fourteen were unique), which are listed in Table 2. Splice site variants contributed to 12/43 (28%) of our confirmed diagnoses, and to 13/47 (28%) of our solved cases. A further three splice site or deep intronic variants in two cases are variants of uncertain significance. A comparison of the scores from the different splicing algorithms for these fourteen unique variants is shown in Table 2 and Additional file 1: Fig. S18.

**Table 2:**
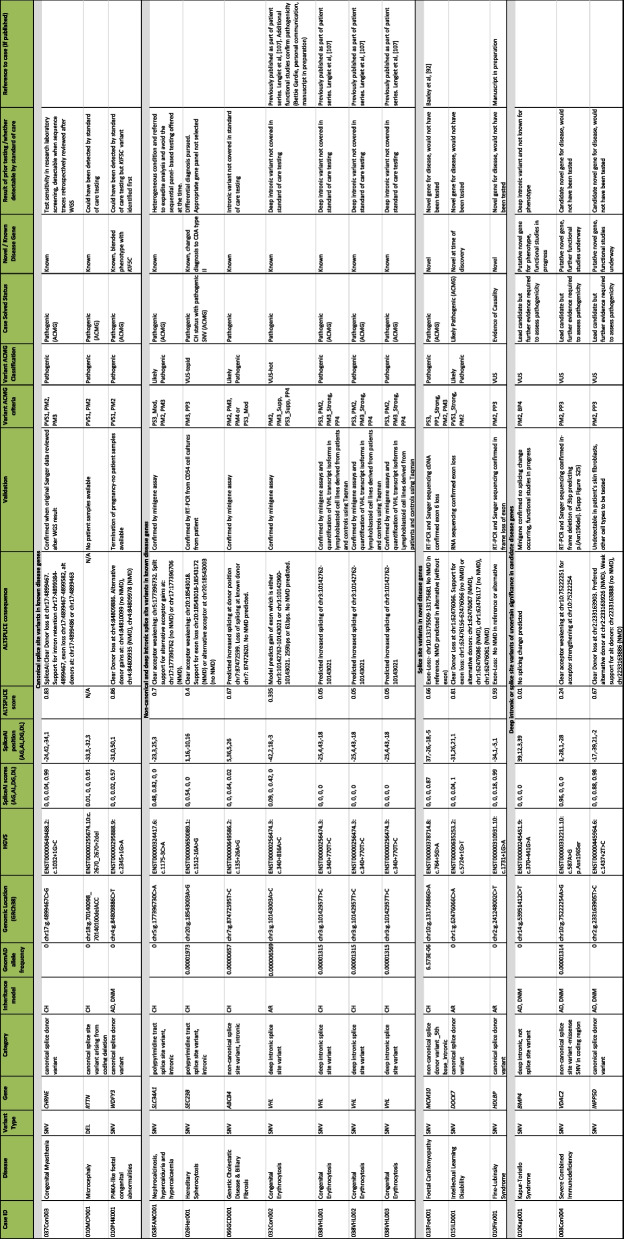
Splice site and deep intronic variants identified in the OxClinWGS cohort

The table is divided into four sections, according to whether variants are (i) canonical splice site variants in known genes (*n* = 3); (ii) non-canonical splice site variants in known genes (*n* = 7, of which 5 are unique); (iii) splice site variants in novel (*n* = 2), or putative novel (*n* = 1) disease genes; and (iv) variants of uncertain significance in candidate genes (*n* = 3). Sixteen splice site variants (fourteen unique variants) were identified in fifteen cases. Thirteen variants contributed to cases which we considered to be solved, as the variants were classified as pathogenic/likely pathogenic (according to ACMG criteria) or were clinically actioned. Ten variants were in known genes for the phenotype, although seven of these were either non-canonical splice sites or deeper into introns and would not have been detected by conventional testing. Two splice site variants were in novel genes (or genes that were novel at the time of discovery) whilst one is in a novel gene with evidence of causality. Three were variants of uncertain significance, two of which were in the same patient which could account for different elements of the patient’s phenotype. We defined a canonical splice site variant as ± 2 bp into intron

Details of prior testing and whether variants could have been detected by the standard of care testing (arrays, panels or exomes) are included

*Abbreviations*: *AR* autosomal recessive, *AD* autosomal dominant, *CH* compound heterozygous, *DNM* *de novo* mutation, *CDA* congenital dyserythropoietic anaemia type II

Three variants involved canonical splice sites in known disease genes. These canonical sites are defined as being the two nucleotide consensus sequences for the 5′ splice donor (GT) and 3′ acceptor sites (AG) and would be expected to be included in flanking regions of exons captured by targeted and exome sequencing so could, in principle, be detected by standard of care testing.

The first canonical splice site variant, a c.1032 + 1G > C variant in *CHRNE*, was identified in a patient with congenital myasthenia having been missed on clinical testing. This could be observed when the original Sanger sequencing traces were reviewed retrospectively after WGS, demonstrating that review of previous testing results prior to ordering WGS may be useful.

A second canonical splice site variant in a patient with microcephaly resulted from a 3 bp deletion at the end of exon 20 of *RTTN*, a gene known to be associated with this condition. The SpliceAI score was high (0.91) emphasising the utility of these algorithms for identifying splice site variants created by small INDELs.

A third canonical splice site variant was a de novo splice donor variant (c.2345 + 1G > A), predicted to be pathogenic, in the known microcephaly-associated gene, *WDFY3* (OMIM **#**617520) [101, 102] in a foetus with congenital brain anomalies including small cerebellum seen on a pre-natal scan. This was in addition to a de novo missense p.(Glu237Gly) variant in *KIF5C* (Additional file 1: Fig. S19), a gene in which pathogenic heterozygous variants cause cortical dysplasia with other brain malformations (CDCBM2, OMIM **#**615282) [103]. Both SpliceAI and ALTSPLICE predicted the loss of a donor site in *WDFY3* on the reverse strand and weakly predicted a donor gain, which would give rise to exon skipping resulting in nonsense-mediated decay (NMD) or an alternative isoform, respectively. The presence of two de novo pathogenic mutations in known disease genes suggests that this patient may have a blended phenotype which may explain the severity of the patient’s microcephaly (see Additional file 2 for further discussion). An early clinical exome study suggested that up to 5% of RD patients may have a phenotype due to two or more single gene defects [104], a value replicated in a more recent study of WES data from 7374 patients [105]. We have not been able to confirm these *WDFY3* and *KIF5C* variants in patient-derived cells, as the original sample was from a termination of pregnancy and no further samples were available, but reporting the additional variant in *WDFY3* should be considered given the possibility of gonadal mosaicism [106] and, consequently, the implications for reproductive risk assessment.

Seven non-canonical splice site variants in known genes were identified. The first was just outside the canonical splice site (c.1175-3C > A) of *SLC34A1* and validated by minigene assay (Additional file 1: Fig. S20) which, together with a second variant c.241dup p.(Glu81fs), confirmed the diagnosis of nephrocalcinosis in this patient.

A c.1512-16A > G variant in the polypyrimidine tract of *SEC23B* decreases splicing efficiency leading to skipping of exon 14. The variant was identified in a patient originally thought to have hereditary spherocytosis (HS) but no pathogenic variants had been found in genes associated with HS in this patient. A second pathogenic variant in *SEC23B*, c.40C > T p.Arg14Trp, was also identified in this patient. *SEC23B* is not a gene associated with HS but is known to be associated with the recessive disorder, congenital dyserythropoietic anaemia (CDAII), which is often mistaken for HS. The WGS finding required extensive investigations of the patient’s blood cells using electron microscopy to confirm the change in clinical diagnosis to CDAII. This highlights the need for the resources, in terms of clinical expertise and costs, which may be required to validate findings arising from WGS, and can be challenging in a clinical setting and requires research support. The intronic c.1512-16A > G variant would not have been included on the targeted panel used for conventional clinical testing.

A third, non-canonical splice site variant, c.135 + 26A > G, was identified in the recessive gene, *ABCB4*, in a patient with genetic cholestasis disease and confirmed by a minigene assay (Additional file 1: Fig. S21). The first hit, a c.2200G > T p.(Glu734*) stop gain variant, had been identified by conventional clinical testing, but this second hit from WGS finally provided a genetic diagnosis for this young patient.

Four non-canonical splice site variants were identified in *VHL.* Whilst biallelic *VHL* variants are known to cause congenital erythrocytosis, the condition with which these patients were referred, these variants were too deep into the introns (> 100 bp from exon/intron boundary) to have been picked up by conventional testing or exome sequencing. In three of the patients, a known pathogenic variant, p.(Arg200Trp), had already been identified as a first hit prior to WGS. WGS identified the same second hit, c.340 + 770 T > C *VHL* variant in the three patients, which resulted in dysregulation of splicing and retention of a cryptic exon, and was confirmed by functional studies to be pathogenic [107]. An additional deep intronic homozygous variant in *VHL*, c.340 + 816A > C, was identified in another patient with congenital erythrocytosis, which was also confirmed to be pathogenic (Betty Gardie, personal communication).

Three splice site variants were identified in genes that are novel or putative novel disease genes, or were novel at the time we identified them, and therefore would not have been investigated by conventional testing, but are canonical splice site variants or are close to intron/exon boundaries. A c.764 + 5G > A variant was identified in *MCM10*, in *trans* with a c.236del p.(Gly79fs) in a patient with restrictive cardiomyopathy. The variants were found to have constrained telomerase activity leading to stalled replication forks and telomere shortening, confirming their pathogenicity [92]. A splice site variant identified in *DOCK7* (c.5724 + 1G > T), in a patient with seizures and severe ILD associated with microcephaly, was a novel disease gene at time of sequencing our patient, but was subsequently reported in patients with developmental and epileptic encephalopathy [108]. This patient also had a duplication in *TP53BP2.* A third splice site variant, in *HDLBP*, leads to loss of an exon in this RNA-binding protein. Whilst a canonical splice site, this gene has not previously been reported as a human disease gene but its likely pathogenicity is supported by segregation in five affected individuals in our consanguineous family, by functional data (loss of RNA binding) and by GeneMatcher hits, including families cited in a previously published report [109].

Three VUS were identified in potential candidate disease genes. A deep intronic, de novo variant, c.370 + 441G > A, was found in a highly conserved region of *BMP4* in a patient with Kapur-Toriello syndrome. This extremely rare condition is characterised by facial dysmorphism, severe intellectual deficiency, cleft lip and palate, skeletal anomalies, ophthalmic features, intestinal and cardiac anomalies and growth retardation. Pathogenic variants in this bone morphogenetic protein have previously been associated with anophthalmia-microphthalmia and digital anomalies [110] (OMIM #607932) and cleft-lip palate [111] (OMIM #600625) and indeed the patient presented with several features of this syndrome including anophthalmia and clefting, and hence, *BMP4* is considered a good candidate by the referring clinician. Current splice algorithms did not suggest that this variant introduced a novel cryptic splice site and indeed minigene experiments did not support an effect on splicing (Additional file 1: Figs. S22 and S23). The variant is predicted by deepHaem to be located within a weak open chromatin site in embryonic lung fibroblasts, creating a sequence which quite closely matches the consensus for a HOX(A/B/D)13 TF binding site (Additional file 1: Fig. S24), a prediction that is currently being experimentally tested. This case demonstrates the challenges associated with validating deep intronic variants which are not cryptic splice site variants and do not have any clear regulatory annotation.

Two further variants of uncertain significance were identified in a patient presenting with T-cell negative, B-cell positive and NK-cell positive (T-B + NK +) severe combined immunodeficiency (SCID) and limb malformations. A de novo, missense variant, c.587A > G p.(Asn196Ser) in *VDAC2*, was predicted by both SpliceAI and ALTSPLICE to lead to creation of an alternative, in-frame splice acceptor site resulting in a new isoform with one less codon, and a reduction in the use of the canonical splice acceptor site, results which have been confirmed by RT-PCR (Additional file 1: Fig. S25). *VDAC2* has not yet been recognised as a disease-causing gene, although it has been described to have a central role in determining thymocyte survival through its regulation of the pro-apoptotic protein, BAK2 [112] and is therefore a possible candidate underlying the patient’s immunodeficiency. We also note the presence of a second de novo variant in this patient, a c.1437 + 2 T > C in intron 11 of *INPP5D*, which is predicted to weaken the canonical splice donor site efficiency and result in usage of an alternative acceptor site leading to a premature stop codon. Again, this gene has not previously been described to be associated with human disease, although in vitro studies have shown that INPP5D (also called SHIP1) is a protein phosphatase which regulates the PI3K signalling pathway and plays a key role in both T cell biology [113] and mammalian skeletal development [114] and it could contribute to one or both elements of the patient’s phenotype. Since the patient had been treated with haematopoietic stem cell transplantation for genetically undefined T-B + NK + SCID during infancy, we could not confirm the splice site variant in blood and, consistent with GTEx predictions, we could not detect it in patient-derived skin fibroblasts. Expression in additional cell types is now being investigated.

Overall, we found the SpliceAI and ALTSPLICE scores to show good correlation (Additional file 1: Figs. S17 and S18) but ALTSPLICE provided additional annotation and experimental hypotheses to test.

### Somatic mosaic variants

Although all cases were referred for germline testing, we highlight the importance of considering somatic variants for RD patients with features of overgrowth syndromes. One patient was referred with Klippel-Trenaunay syndrome, a rare disorder, presenting at birth, characterised by vascular and lymphatic anomalies and abnormal veins in association with overgrowth of soft tissue and bone. Some cases of familial inheritance or de novo germline variants have been described [115]. Germline analysis of our patient revealed a de novo c.535 T > G, p.(Lys179Val) variant in *RBPJ.* Although the variant involves the last base in exon 6, RNA analysis indicated no effect on splicing, consistent with in silico predictions (Additional file 1: Fig. S26), so the variant was not considered pathogenic. Somatic mutations occurring shortly after birth in the primitive cells destined to become the blood and lymphatic vessels have been described to be causative for this condition [115], but often occur at very low frequency making them difficult to detect by standard coverage WGS. Indeed, we identified a somatic, mosaic c.3140A > G p.(His1047Arg) *PIK3CA* mutation in our patient at low (8%) frequency using targeted high coverage NGS.

### Clinical impact

Our results informed the diagnosis of RD patients in this cohort and, additionally, influenced treatments (see Fig. 2).

#### Impact on clinical diagnosis

For six patients, the genetic diagnosis led to a change in the clinical diagnosis with the identification of pathogenic variants from WGS. The diagnosis of a patient referred with Aicardi syndrome was changed to DEE1 on discovery of a structural variant in *ARX* (see SV section above)*.* Two patients referred with Fine Lubinsky syndrome and found to have pathogenic variants in *POR* and *SLC39A13*, respectively, had their clinical diagnoses changed to Antley-Bixler and spondylocheiro-dysplastic Ehlers-Danlos syndromes, respectively. The clinical diagnosis of two brothers referred with familial juvenile hyperuricemic nephropathy was revised to papillorenal syndrome following identification of a *PAX2* pathogenic variant, a diagnosis which was confirmed by ophthalmological investigations [116]. A family originally diagnosed with Majeed syndrome had their diagnosis changed to PAPA syndrome on identifying a *PSTPIP1* variant whilst another family received a revised clinical diagnosis of CDAII further to identification of a *SEC23B* variant, when originally diagnosed with HS (see the “Results” section).

In other cases, the genes identified were novel at time of discovery in our WGS programme, including *DOCK7* for ILD and *SAMD9L* for autosomal dominant ataxia-pancytopenia syndrome; therefore, they would not have been picked up by prior testing and genetic diagnoses could be provided for the first time for these patients. Phasing of the de novo variants in *SAMD9L* was undertaken using nanopore long read sequencing [83, 117].

#### Expansion of phenotypic spectrum

For some cases, variant identification expanded the phenotypic spectrum associated with a given gene. Pathogenic variants in *RMND1* were originally described for a patient with combined oxidative phosphorylation deficiency 11 (COXPD11), characterised by neonatal hypotonia and lactic acidosis as well as infantile onset renal failure, hearing loss and multi-organ defects. More recently the genotype–phenotype spectrum of this mitochondrial disorder has been expanded [118] but polymicrogyria, which was confirmed on neuropathological examination of the foetus’ brain, has not previously been reported in *RMND1*-related disorder and this, in the context of arthrogryposis, may be an early indicator of an *RMND1* disorder (Additional file 1: Fig. S27).

#### Impact on treatment

Provision of a genetic diagnosis can have a profound impact on the treatment of patients, and in five of our patients, it was considered life-saving; a family diagnosed with the newly described arrhythmia syndrome, cardiac ryanodine receptor (RyR2) calcium release deficiency syndrome [119], was prescribed flecainide for protective effect against ventricular arrhythmia and sudden cardiac death [120].

A 32-year-old woman who had been admitted to an emergency department with presumed meningoencephalitis, and who had had several prior episodes of coma, was found to have biallelic variants in *CFI*, indicating a non-classical presentation of Complement Factor I deficiency*.* Her sister had died of fulminant haemorrhagic leukencephalopathy at the age of 16 years, demonstrating the severity of this condition if left undiagnosed and untreated. The clinical management of this patient’s condition involves optimisation of vaccination strategies and prophylactic use of antibiotics [121].

The identification of a variant in *SLC5A7* in a patient with congenital myasthenic syndrome (CMS) indicated that the patient had the very rare and severe CMS Type 20 [122], characterised by life-threatening respiratory episodes, which benefit from cholinesterase inhibitors, and potentially, salbutamol.

An adult patient with congenital neutropenia and inflammatory bowel disease, a condition which can lead to fatal infections, showed clinical remission of his G6PC3 deficiency further to haematopoietic stem cell transplantation (HSCT) [123].

Identification of compound heterozygous variants in *NPHP3* provided a diagnosis of nephronopthisis type 3 in a family with fibrotic kidney disease. Confirmation of genetic status in a clinically unaffected sibling enabled a successful kidney donation to his affected brother, who would otherwise have had to wait for a deceased donor kidney.

Variants in genes linked to perturbations in metabolism provided readily accessible treatments; one patient’s congenital erythrocytosis was found to be a consequence of hypermanganesaemia caused by a missense variant in the manganese transporter *SLC30A10.* This gene would not have been routinely screened when erythrocytosis is the primary referral condition, although erythrocytosis is a known feature of these transporter defects due to inhibition of the iron centre of the hypoxia-inducible factor prolyl hydroxylase enzymes by the retained transition metals. Early diagnosis allows patients to be treated before the accumulation of manganese deposits causes irreversible damage to the central nervous system and liver. As a result of the WGS finding, our patient was treated with manganese-chelating drugs, sparing her the need for phlebotomy and, potentially, any longer-term organ damage. A variant in *SLC4A1* giving rise to pseudohypokalaemia was the cause of another patient’s apparent potassium deficiency, resulting in cessation of supplements.

Finally, the patient diagnosed with Klippel-Trenaunay syndrome, with splenic and hepatic haemangiomas and telangiectatic lesions of the right hindquarter (discussed above), was found to have a somatic mosaic mutation in *PIK3CA.* This patient is now being recruited to EPIK, a randomised controlled study of the PI3K inhibitor, alpelisib, for treatment of *PIK3CA* overgrowth syndromes.

#### Secondary findings

We identified two patients with secondary findings in this cohort. The first was a variant in *FBN1*, a gene known to be associated with Marfan syndrome. Using the framework for secondary findings that we had established [34], the patient was re-consented for her interest in receiving secondary findings and referred to the cardiovascular genetics clinic. She was found to have mild aortic root dilatation (41 mm at Sinus of Valsalva) on transthoracic echocardiogram. Physical examination revealed dental crowding, abnormal upper segment to lower segment and arm span to height ratios, a positive thumb sign, mild chest wall asymmetry and reduced (mild) extension at the elbows. Cascade evaluation of family members was arranged and her son was found to show physical signs of Marfan syndrome and genetic testing confirmed that he had inherited the same c.4265del p.(Asn1422fs) variant in *FBN1*. Both patients are now followed up regularly in clinic.

This mother had originally been referred to a neuropathy clinic for bilateral *pes cavus* and distal muscle wasting at the age of 43. Nerve conduction studies were consistent with a symmetrical, length-dependent axonal sensorimotor neuropathy and the clinical presentation was compatible with Charcot-Marie-Tooth disease type 2 (CMT2). Interestingly, we identified a heterozygous frameshift variant p.(Asn3232fs) in *DST*. Biallelic variants in this gene are known to cause the recessive disorder, hereditary sensory and autonomic neuropathy type VI (HSAN6), which is associated with congenital insensitivity to pain and autonomic dysfunction (and relative sparing of large fibre function [124]). The *DST* variant in our patient was not considered to be pathogenic for the patient’s CMT2 given the distinct phenotype and the fact that, to date, all *DST* variants causing HSAN6 are biallelic, and the patient remains without a diagnosis for her CMT2.

A secondary finding was also found in the father of a patient with spastic paraplegia. He had a well-recognised variant in *KCNQ1*, p.(Arg192fs) (ClinVar, VCV000053072.23), a gene known to be associated with long QT syndrome that was included in the ACMG list of 56 genes recommended for screening [39]. The subsequent cardiac investigations in this competitive cyclist, and his perspectives on secondary findings, have been described elsewhere [125]. The pertinent finding which would account for his daughter’s spastic paraplegia has not yet been identified.

Both of these secondary findings led to the diagnosis of medical conditions which are potentially life-threatening, but which can be effectively managed given appropriate clinical intervention.

## Discussion

Since 2010, there have been substantial advances in the technology applied to detecting genetic determinants of RD, and genetic diagnoses for many patients and their families have been established. WES is now widely used and provides a cost-effective approach for identifying variants in the coding genome. Low coverage WGS has been described as an alternative to microarrays to identify constitutional CNVs [126] and whilst this has been applied in population genomics studies, the low read-depth means that it cannot be used to robustly identify individual genotypes for rare disease patients [127]. Most recently, standard coverage WGS has provided a platform for hypothesis-free interrogation of variants throughout the genome and new, improved sequencing technologies, bioinformatics pipelines and algorithms and disease gene annotations, combined with gene matchmaking initiatives, have enhanced the speed and efficiency of WGS testing. This has given confidence and some success in moving beyond the classical disease areas of clinical genetics, such as intellectual disability and complex developmental disorders, into undiagnosed diseases of all organ systems, including metabolic disorders, immune deficiencies, haematological and cardiac conditions, as well as neurological disorders, whenever a genetic cause is suspected.

There is good evidence that genome sequencing has increased yields compared with those obtained with standard of care testing. When considering only infantile congenital anomalies or paediatric intellectual disability, a systematic review by the ACMG reported a diagnostic yield of 38% for exome/genome sequencing, compared with 21% for standard of care testing [128]. However, it can be anticipated that achieving these diagnostic yields would be even more challenging in cohorts such as our own, where the age of presentation of patients is mixed (covering both early and later-stage onset conditions), and where the range of conditions being referred covers all medical specialties. Consistent with this, the pilot WGS study reported by Genomics England yielded a 25% success rate even though this cohort was only lightly pre-screened for variants in known genes [13]. A major reason for this is that whilst entire genomes have been sequenced, the analysis has largely been restricted to the exome, and therefore, the full value of WGS has not yet been realised. If WGS is to be used as a first-line test in clinical genetics services in any country, it must be able to achieve a reliable diagnostic yield in patients referred for a broad range of conditions and ages of presentation.

We have addressed this deficiency by intensively studying a large cohort of families referred with a broad range of RD from multiple medical specialties, avoiding disease domains where diagnostic yield has been high because of the burden of de novo variants, such as intellectual disability. In addition to careful scrutiny of the coding genome, we developed and utilised a range of tools necessary to properly evaluate other sources of relevant variation around the genome.

This included searching systematically for structural, splicing and non-coding variants with the expectation that these tools would reveal more relevant variation that mediates disease. The new applications come from other groups as well as our own and add considerably to the effort required to identify relevant variation. By applying these tools, we find that this wider set of variants contributes to considerably enhanced success rates, now reaching 35% confirmed diagnoses, or 39% when considering all cases with evidence of causality in a cohort that had been pre-screened according to standard of care testing available at the time. This demonstrates the importance of investigating all variant types to maximise diagnostic yield. Although WGS is not the only method to achieve this and, for example, some CNVs can be detected by WES or arrays, our results demonstrate that 10/43 (23%) of our diagnosed cases would not have been detected or reportable to clinical standards by WES, even when retrospectively investigating these (Tables 1 and 2 and Additional file 3: Table S7). In particular, many structural variants and variants in deeper intronic regions are missed by WES.

The identification of SVs from WGS data presents considerable challenges; high GC-rich regions lead to uneven read depth whilst short sequencing reads are difficult to map uniquely to highly repetitive regions of the genome. Combining algorithms that are based on different theoretical models helped to reduce the false discovery rate of CNVs whilst retaining sensitivity.

In our study, SVs accounted for 4/43 (9%) of our confirmed diagnoses and 7/47 (15%) of the cases we consider to be solved. Four SVs could, in principle, have been detected by arrays, but were not identified by standard of care testing due to inadequate probe coverage, or thresholds used, or because findings were of uncertain significance due to the novelty of the genes concerned. Our analysis explicitly screened for deletions that are too large to be called by small variant callers and too small to be confidently called by array CGH and this WGS analysis revealed the 3 kb deletion in *ARX* described above*.* Of the seven SVs we have described, three SVs involved specific genes, two involved flanking regions of single genes, whilst two were complex rearrangements including a case of chromoanasynthesis that provides a novel mechanism of disease for maxillary prognathism.

We required a range of techniques to validate the SVs (Table 1), demonstrating the complexity of doing this in a routine clinical setting. Long-read sequencing from providers such as Oxford Nanopore and PacBio can be important in overcoming some of the limitations of short-read sequencing [18] and, with reductions in cost and improvements in error rates, could potentially be used at scale. Genome optical mapping (Bionano) provides an important orthogonal technique for identification or confirmation of SVs, as this approach is not subject to the same limitations of sequencing such as repetitive regions of the genome, which challenge both short- and long-read sequencing technologies [129].

Splice site variants, including some distal to exons, contributed to our diagnostic yield. Algorithms such as SpliceAI, which use deep learning to predict splice sites from primary sequence, have been highly effective at identifying putative splice site variants. We have also utilised a novel algorithm, ALTSPLICE, which provides additional predicted impact annotations for splice site variants, including synonymous variants. These showed similar performance (Table 2, Additional file 1: Figs. S17 and S18 and Additional file 3: Table S9) but the additional annotation from ALTSPLICE was useful in informing experimental follow-up. Splice site and deep intronic variants contributed to 12/43 (28%) of our confirmed diagnoses and 13/47 (28%) of the cases we consider to be solved (Table 2). Three variants were canonical splice site variants in known disease genes which could have been picked up by standard of care testing. However, the seven non-canonical splice site variants in known genes and the three splice site variants in novel genes would have been missed by routine testing, as would the three candidate splice site VUS. Where possible, we validated splice site variants using RNA-Seq, RT-PCR or minigene experiments but such validation can be challenging if the gene in question is not expressed in blood or skin, or if patient material is not available (e.g. in this study, RNA from the foetus with a *WDFY3* splice site variant). Ideally, quantitative transcript expression of both wild type and variant is necessary to confirm that the splicing from the variant is not ‘leaky’. Minigene experiments are a relatively straightforward way to confirm splice site variants but conducting these at scale nonetheless requires dedicated resources and expertise.

Identifying and confirming pathogenicity of non-coding variants is a major challenge due to our limited understanding of their impact and paucity of representation in genomic databases. In our study, application of GREEN-DB led to identification of candidate variants in several cases including a deep intronic variant in *BMP4* in a patient with Kapur-Toriello syndrome and variants in *HSD3B7* and *BMP6* in a patient with neonatal haemochromatosis. The *HSD3B7* variants are located in a conserved region at the 3′-UTR end of the gene so may affect polyadenylation and stability. Further annotation with deepHaem predicted that the *BMP4* intronic variant is located in a weakly accessible open chromatin site in embryonic cells, which could affect HOX TF binding, a hypothesis which is currently being experimentally tested.

Functional validation of known and novel genes is also challenging. Several of our results could, however, be confirmed by routine clinical blood-based assays. For example, the absence of complement factor I due to a *CFI* variant was confirmed by serum electrophoresis, and manganese excess and deficiency of potassium resulting from *SLC30A10* and *SLC4A1* variants, respectively, could similarly be confirmed from plasma samples. Metabolomics has informed investigation of other genes where perturbation of a metabolite was suspected, such as *DHRS3* (discussed above). Other variants, even those in known genes, took substantial effort and resources to validate such as a p.(His457fs) in *FOXN1* which revealed a novel dominant negative disease mechanism [130, 131], and confirmation of the effect of *SEC23B* variants which required detailed electron micrographs to differentiate between diagnoses of hereditary spherocytosis or CDAII. Some functional studies refuted pathogenicity of apparently good candidate variants, one notable example being a *PIGA* variant, p.(Lys78Glu) in a patient with microcephaly which did not affect cell-surface expression of GPI-anchored proteins (Additional file 1: Fig. S28) and is classified as likely benign (SCV000891722.1) [89].

Our results demonstrate the importance of using multiple, complementary genomics algorithms and tools to interrogate the different variant types, in particular those which are based on different theoretical assumptions and therefore complement each other. Our pipeline is not unique but gives examples of the types of algorithms that can be combined to interrogate different variant types and can be applied in any clinical or research setting in any country. When these computational approaches are combined with candidate gene prioritisation based on the phenotype profiles (using tools such as GADO or Exomiser), this can enhance our ability to provide a genetic diagnosis and discover new disease genes. Our study highlights the importance of collecting detailed patient phenotypes in a standardised manner through the use of HPO terms to guide candidate variant selection especially when expanding genetic analysis to non-coding regions.

Overall, SVs and splice site variants contributed to 20/47 (43%) of our solved cases. Even when discounting the three SVs and three canonical splice site variants in known disease genes, the fact that 14/122 (11%) cases had SVs or splice site variants that would only have been detected or resolved by WGS demonstrates the importance of incorporating tools for the analysis of these variants in the bioinformatics pipeline to maximise the diagnostic yield.

This study has led to the discovery of eight disease genes which are novel (*MCM10*, *KMT2E*, *POLR2A)*, were novel at time of discovery (*DOCK7*, *SAMD9L*) or are putative novel disease genes with evidence of causality (*DHRS3*, *FOXD3*, *HDLBP).* VUS in candidate genes are being further investigated. The phenotypic spectrum of one gene, *RMND1*, was expanded to include polymicrogyria, whilst *BMP4* is being investigated as a candidate for Kapur-Toriello syndrome.

The OxClinWGS study has made a substantial contribution to the diagnosis and treatment of the patients recruited. Overall, 43 patients had ACMG confirmed pathogenic/likely pathogenic variants or clinically reported variants giving a diagnostic yield to date of 35% whilst a total of 47 patients (39%) are considered solved if cases with variants in novel disease genes, with evidence of causality, are included. We note that the ACMG classification system did not capture all variants in known genes which we considered to be pathogenic, and which were clinically actioned for the diagnosis and treatment of patients. An additional classification category, encompassing response to treatment, may enhance the ACMG criteria. The clinical diagnosis of six probands in our cohort was changed on the basis of the WGS results and two patients with secondary findings requiring clinical intervention were identified. The overlapping features between many of the clinical genetic syndromes, particularly neurodevelopmental disorders, support the view that genetics is providing a new taxonomy of disease based on genetic mechanism rather than constellation of clinical symptoms. This information is not just for classification purposes but, most importantly, guides appropriate treatment selection and avoidance of inappropriate therapy. We report several cases where this treatment selection could be life-saving; for example in cases of primary immunodeficiency where appropriate vaccination and prophylactic use of antibiotics can assist with avoidance of life threatening infections, in sudden cardiac death syndromes where pharmacological intervention can be an alternative to ICDs, in congenital myasthenic syndromes where understanding whether the defect in neuromuscular transmission requires inhibition or augmentation of the synapse is central to treatment selection and in congenital neutropenia where HSCT, even in adulthood, was effective.

It is now over a decade since we started our first WGS study of patients [23], and our experience in the OxClinWGS cohort highlights the importance of more intense interrogation of all variant types, and the challenges associated with this. It also continues to demonstrate the importance of close interaction between clinicians, scientists and bioinformaticians in clinical and research communities. This starts at the referral process ensuring that a detailed clinical description of a patient’s condition is provided (including accurate HPO terms), alongside results from any prior genetic testing undertaken. It continues during the analysis as bioinformaticians and genomics scientists analyse the WGS data and propose candidates, which may be accepted or refuted, and clinicians suggest candidate genes and pathways they suspect may be involved. This is an ongoing, iterative process which can be difficult to achieve in large-scale national programmes, and since clinical laboratories do not have the mandate or resources to explore variants outside known disease genes, this must involve the research community. For the 100KGP, Genomics England Clinical Interpretation Partnerships were established in part for this purpose, and their success requires referring clinicians and researchers to be directly involved. Virtual MDT-style meetings can provide an important mechanism for this.

Nonetheless, large-scale, national programmes have the tremendous advantage of standardising collection of samples, data and analysis and can achieve costs substantially below anything that can be achieved in localised clinical settings [30] as has been demonstrated by the 100KGP [13]. This then opens up the opportunity for patients with a wide range of undiagnosed rare disorders, which are likely to be monogenic in origin, to be referred for WGS. In summary, our OxClinWGS study shows that structural, splice site and deep intronic variants make important contributions to the diagnostic yield of genome-wide sequencing for rare disease patients and a range of different sequencing methodologies and bioinformatics pipelines may be needed to identify this range of variant types. Validating such variants at scale remains challenging from both genetic and functional perspectives and will rely on international collaboration to create and populate databases. Now that WGS is becoming a mainstream genomics test in clinical medicine, interrogation of all types of genetic variants must be included in analytical pipelines if diagnostic yield is to be increased.

## Conclusions

Genome sequencing is increasingly being adopted in the clinic as a technology platform for providing genetic diagnoses for patients with rare diseases. However, if the analysis of the genome is confined to in silico gene panels or coding regions of the genome, pathogenic structural, splice site and deep intronic variants may be missed. Comprehensive analysis of the full genome sequence should be undertaken if the diagnostic potential of genome sequencing is to be realised.

### Supplementary Information

**Additional file 1. **Supplementary methods, figures S1-S28 and references.

**Additional file 2. **Supplementary clinical case information for selected cases.

**Additional file 3. **Supplementary Tables S1-S9.

## Data Availability

Variant data have been deposited in NCBI NLM ClinVar (www.ncbi.nlm.nih.gov/clinvar) with the following accession numbers: SCV000891722 (*PIGA*) [89] SCV001548171 (*FOXN1*) [131] SCV003853383-SCV003853459 [73] for all other variants submitted as part of this study. Sequence data has been deposited at the European Genome-phenome Archive (EGA), which is hosted by the EBI and the CRG, with the following accession numbers: EGAS00001003469 (ataxia-pancytopenia syndrome and severe immune dysregulation patient with variant in *SAMD9L*) [117]. https://ega-archive.org/studies/EGAS00001003469 EGAS00001007575 (craniosynostosis cases 007Cra001 (proband) and 007FAM001 (affected mother and daughter)) [73]. https://ega-archive.org/studies/EGAS00001007575 ALTSPLICE data are available on GitHub [74].
