# Supplementary material for: Structural and non-coding variants increase the diagnostic yield of clinical whole genome sequencing for rare diseases
Source: Genome Med. 2023 Nov 9;15:94. doi: 10.1186/s13073-023-01240-0 (PMC10636885; doi:10.1186/s13073-023-01240-0)
Supplement: Supplementary file 1 — Additional file 1. Supplementary methods, figures S1-S28 and references. [file 13073_2023_1240_MOESM1_ESM.docx]

Supplementary Information

Structural and non‑coding variants increase the diagnostic yield of clinical whole genome sequencing for rare diseases

Table of Contents

[Supplementary Methods 2](#_Toc132836080)

[Sequencing and alignment 2](#_Toc132836081)

[Quality checks 2](#_Toc132836082)

[Variant identification 3](#_Toc132836083)

[Variant annotation 4](#_Toc132836084)

[Non-coding variant annotation 4](#_Toc132836085)

[Interactive analysis of pedigree data 4](#_Toc132836086)

[Cohort analysis 5](#_Toc132836087)

[Splice site analysis 5](#_Toc132836088)

[Somatic variant analysis 6](#_Toc132836089)

[HPO analysis 7](#_Toc132836090)

[Bionano analysis 7](#_Toc132836091)

[Ultra-high molecular weight DNA extraction and labelling 7](#_Toc132836092)

[Data collection 7](#_Toc132836093)

[Bionano rare variant analysis and structural variant calling  7](#_Toc132836094)

[Functional Studies 8](#_Toc132836095)

[Minigene assay for *SLC34A1* splice site variant 8](#_Toc132836096)

[RT-PCR assay for *SEC23B* splice site variant 8](#_Toc132836097)

[Minigene assay for *ABCB4* splice site variant 8](#_Toc132836098)

[Minigene assay for *BMP4* variant 9](#_Toc132836099)

[RT-PCR assay for *VDAC2* splice site variant 9](#_Toc132836100)

[RT-PCR assay for *RBPJ* variant 9](#_Toc132836101)

[Expression of *ALG13* wild type and mutant 9](#_Toc132836102)

[Supplementary Figures 11](#_Toc132836103)

[Supplementary References 36](#_Toc132836104)

# Supplementary Methods

A schematic summarising the study and our main analysis workflows is depicted in Additional file 1: Fig. S1. A full description of sequencing and bioinformatics methods is provided below.

## Sequencing and alignment

DNA was extracted from peripheral blood using the Gentra Puregene kit (Qiagen) or similar methodologies. TruSeq PCR Free libraries (Illumina) were prepared using manufacturer’s guidelines. Sequencing was performed on either the HiSeq2500 in rapid or High Throughput mode (HTv4), or the HiSeq4000 instrument using 2x100bp, 2x125bp or 2x150bp pair-end reads, respectively. If DNA was limiting, for example for certain foetal cases, a PCR-based approach was used. Our sequencing strategy aimed to generate a minimum mean coverage of 30x. The actual sequencing depth is shown in Additional file 1: Fig S2.

Sequencing data were initially uploaded and analysed using Illumina’s BaseSpace cloud computing service. For clinical cases, VCF files were first analysed using VariantStudio (Illumina) in the clinical genetics laboratory using locally-curated gene panels. Clinical cases that remained unsolved and research cases were analysed using a range of tools but in all cases this included Ingenuity Variant Analysis (Qiagen) which has now transitioned to QCI translational.  The flexibility of this commercial filtering tool facilitated the use of customised filter settings tailored to each particular case.  It also enabled us to explore different inheritance models and QC filtering thresholds.  A benefit of this flexible approach is exemplified by the *de novo* p.(Arg380His) variant in *TUBB2B* which lay in a segmental duplication and hence was initially excluded by our standard QC filters.

Sequencing and initial data analysis was undertaken over a period of 2-3 years and the analytical pipeline changed during that time. Therefore, to ensure all samples had been analysed in a unified way, at the end of the project a single pipeline was designed and was used to reanalyse all samples in parallel.

The pipeline used for read alignment, variant calling and ROH detection is described below and available as a Nextflow workflow on github [1]. Reads were aligned to GRCh38 using bwa v0.7.15 [2], and duplicated reads marked using samblaster v0.1.24 [3]. Low mappability regions were computed for GRCh38 reference genome using GenMap [4] and a 150bp kmer size, corresponding to the read-length used in WGS sequencing.

## Quality checks

First, the distribution of read length and quality metrics was inspected using *FastQC* ([www.bioinformatics.babraham.ac.uk/projects/fastqc/](https://www.bioinformatics.babraham.ac.uk/projects/fastqc/)) to ensure the quality of raw data. We then used *mosdepth* [5] to compute coverage distribution across the whole genome (Additional file 1: Fig. S2) and across genes defined in GENCODE v31 and removed any samples *with* less than 75% of bases covered at least 10X across the genome. Finally, we used somalier [6] to compute ancestry components per individual based on the 1000G phase 3 reference panel (Additional file 1: Fig. S3). The same tool was used to compute pairwise relationship values and genetically inferred gender and rate of heterozygosity (Additional file 1: Figs. S4 and S5). Any sample with information discordant with data from the clinical report, and samples with an unexpectedly high rate of heterozygosity, indicating possible sample contamination, were removed from the cohort.

## Variant identification

We processed BAM files to identify different kinds of genetic variations, namely small variants (SNVs and small INDELs), large variants (copy-number variations and structural variants), repeat expansion variants, and regions of homozygosity (ROH). An outline of the bioinformatics pipeline is shown in Additional file 1: Fig. S1.

Small variants (SNVs and INDELs) were identified from single individual BAM files using *deepvariant* v1.0.0 [7] with WGS mode and single individual gVCF were merged into a single cohort VCF using *GLnexus* v1.2.6 with deepvariantWGS optimised settings [8]. Variants were filtered retaining only variants with quality above 20 and at least 1 individual with GQ ≥ 20. All genotypes with DP = 0 or GQ = 0 were then set to missing to remove spurious homozygous reference genotypes generated by GLNexus in the merging process. Variants were then decomposed and normalised using *bcftools* norm (v1.11). The resulting ‘1/.’ half-genotypes were converted to heterozygous calls and the ‘0/.’ to homozygous reference. Filtered VCF files were then post-processed by a custom python pipeline, available on github, [9] that orchestrated *SnpEFF* v4.3t [10] and *vcfanno* v0.3.1 [11] tools to add rich sets of variant, gene and genomic location based GRCh38.36 annotations (Additional file 3: Table S1).

Large variants were detected using a combination of *Lumpy* v.0.2.13, *CNVnator* v0.4.1, *SVTyper* v0.7.1 and *svtools* v0.5.1 as described [12]. These were chosen as they are based on different theoretical models and performed well in a recent comparison [13]. Briefly, possible large events were first identified separately from each single BAM file using *Lumpy*, excluding segmental duplication, centromere, telomere and microsatellite regions. Calls were then merged and refined using *svtools* to generate a cohort-wide representation of likely distinct events. These events were then genotyped per individual using *svtools* and annotated for copy-number state using CNV states computed by *CNVnator*. The resulting genotyped VCFs were finally merged into a single cohort VCF and variants likely representing the same event were further pruned using *svtools*. The resulting calls were then processed using *‘svtools* classify’ which converts low-supported events into BNDs pairs. Following suggestions from the *svtools* publication, detected events were filtered as follows: i) Minimum length of 50 bp; ii) Small deletions shorter than 1kb to be supported by split reads in at least 1 individual; iii) Inversions must have a mean sample quality ≥ 150 and the split reads and pair-end reads must represent at least 10% of the supporting reads; iv) BND events must have a mean sample quality ≥ 250; v) Deletions and duplications must have a mean sample quality ≥ 40; vi) For small events shorter than 5kb, less than 70% can overlap with low complexity + low mappability regions.

We then used an overlap-based approach to annotate large variants. Each variant was annotated with the population AF from 1000G phase3, gnomAD v2 and variants derived from Abel *et al* [14], when it was overlapped by at least 75% by a variant of the same type, retaining the maximum AF when multiple overlapping events were observed. Each variant was also annotated with regulatory regions from GREEN-DB if it overlapped by at least 10%. We also applied *SVRare* [15] for large variant discovery. Briefly, Illumina *Manta* (v1.6.0) was used to call structural variants. Inversion events were later added as per Manta documentation. The resulting data were imported together with the *svtools* results to a sqlite3 database. Events were merged using a similarity threshold of 0.5 and were annotated using gnomAD SV (v2.0), dbVAR and Decipher [16]. Structural variants were prioritised and visualised using *SVRare-js*, which is available on github [17].

Repeat expansion variants were detected for a set of known expansions in 29 genes using *ExpansionHunter* v3.2.2 [18] and the corresponding variant catalogue. *ExpansionHunter* provides for each candidate locus an estimate of the number of repeats and the genotype. Results are returned in JSON and VCF format. The resulting VCF files for single individuals were merged by family using *bcftools* to evaluate variant segregation in each pedigree.

Regions of homozygosity were detected for each sample directly from the cohort VCF using *bcftools roh* based on genotype likelihoods. The resulting events were filtered for quality ≥ 10. To assess possible consanguinity, we computed the total fraction of genome contained within ROH regions and the fraction contained in large (> 2Mb) ROH regions per individual.

## Variant annotation

For each case, a ranked list of genes potentially relevant for the family phenotype was calculated based on the respective HPO profile using *GADO* v1.0.1 [19]. Genes above the 90th percentile in the *GADO* ranking were selected as best candidates. Pedigrees were further analysed with *Exomiser* v12.1.0 [20] using data release 2102 and a portable version of this HPO prioritisation pipeline is publicly available at [21]. Candidate genes were further annotated using pLI values from *gnomAD* v2.1.1, the GDI score (human damage index) [22] and the RVIS (Residual Variation Intolerance Score) value [23] based on ExAC v2.

### Non-coding variant annotation

We used GREEN-DB v2.5 and GREEN-VARAN v.1.0 workflow [24] to add additional annotations useful for the prioritisation of non-coding variants. The GREEN-DB is a large collection containing about 2.5M regulatory regions distributed across the human genome and including information on the region function (i.e., enhancer, promoter, silencer), their tissue of activity and controlled gene(s). A constraint metric representing tolerance to mutation for each regulatory region is also provided. Using the GREEN-VARAN workflow we annotated small and large variants for possible overlaps with GREEN-DB regulatory regions to be able to associate non-coding variants with putative target genes. Additionally, the workflow also adds annotations for overlaps with TFBS and DNAse sites from the ENCODE dataset as well as 3 non-coding variant prediction scores, namely ReMM v0.3.1 [25], FATHMM-MKL [26] and ncER [27]. All these annotations were then used in the Variant Explorer app and in the cohort analysis (see below) to prioritise non-coding variants impact and link them to genes. GREEN-DB and GREEN-VARAN are available at [28].

Potential consequences of a subset of NCVs were additionally predicted using deepHaem, a deep convolutional neuronal network for predicting chromatin features from DNA sequence trained previously [29], [30] on 4,384 ENCODE data [31] and available on GitHub [32]. Damaging scores were calculated by subtracting the predicted chromatin class scores of the variant sequence from the reference sequence (hg38) and ranking all classifiers.

## Interactive analysis of pedigree data

We developed Variant Explorer (VE), a graphical user interface to allow disease experts to interactively explore the results generated for each pedigree by the bioinformatic analysis described above using segregation filters and the rich set of variant annotations to identify new candidate variants [33]. VE was developed in R using *Shiny* and *shinydashboard* to implement the graphical user interface. First, annotated cohort-wide VCF files containing small and large (SV) variants were filtered and pre-processed by a custom python script (*cohort_varan, see below*). Variants were filtered for maximum population AF (2%) unless they were known in annotated disease databases (HGMD, ClinVar, Cosmic). Support for different inheritance models (recessive, dominant, *de novo*) per pedigree was calculated after filtering variant calls for minimum genotype quality (10/20 for SNVs and *de novo* variants respectively). Overlap with annotated genic regions (exons, introns, untranslated regions (UTR), splicing and regulatory regions) was determined and results were written to various output tables per pedigree (variants, known variants, affected genes, compound heterozygous variant pairs). These tables were then loaded into R and combined with results obtained from other analyses (ROH regions and repeat expansions), PED files of the cohort pedigrees, and the list of associated HPO terms. The complete R objects were then loaded in the R shiny app to perform sophisticated variant filtering based on variant annotations and segregation among family members. The flexible filtering system allows to apply different filtering strategies to specific variant groups defined by variant consequences and perform complex segregation filtering, such as selecting compound heterozygotes involving variants with a specific effect. Further filtering can be applied based on ROH regions, additional regions of interest provided in a BED file or gene-based annotations like GADO score. After filtering, the application presents results in a variant or gene centric view ranking results by GADO score of the affected gene so that variants affecting genes more likely related to the phenotype are prioritised. Additionally, the application includes disease-related gene panels from PanelApp [34] and the ability to provide custom gene lists to highlight genes of interest among filtered candidates. Finally, for a single result the application can retrieve detailed information on the variants involved, including full annotations and regulatory information from GREEN-DB, and the affected gene, including Gene Ontology annotations, REACTOME pathways annotation, associated diseases from ClinVar and tissue specific gene expression from GTeX.
Cohort_varan code is available at <https://github.com/popitsch/cohort_varan>, VE code is available at <https://github.com/edg1983/Variant_explorer>.

## Cohort analysis

The final cohort contains 300 individuals, including 34 singletons and 88 families (duos, trios and larger pedigrees). To conduct cohort-wide analyses, we integrated the various output files from the bioinformatics pipeline (e.g., annotated VCFs, GADO and Exomiser result tables, etc.) as well as several additional annotation resources (e.g., PED files and gene annotations) into a partitioned parquet database using custom python scripts based on apache arrow, pysam, numpy and pandas libraries. Variants were assigned unique ids and some annotation fields were summarised during import (e.g., a maximum global population allele frequency was calculated from various input sources such as gnomAD, UK10K and 1000G). Segregation patterns were derived from quality-filtered genotype calls and potential *de novo* calls were quality-filtered more stringently (filtering for segregation, parent genotype qualities, read depth, allelic depth, allelic balance and allele quality where available; SV/CNV calls were filtered for allele quality and svtyper sample quality (SQ) values). Cohort-wide statistics (including cohort-wide variant frequencies) were calculated and added to the database. Variant consequence predictions from SnpEff and GREEN-DB were mapped to a ranked list of sequence-ontology terms (e.g., to predict the most severe consequence of a variant with respect to known transcripts of a gene). Variant locations relative to gene and transcript annotations (e.g., UTR, splicing, intronic) were calculated. Associations between variants and genes/transcripts were derived from calculating overlap with gene annotations and by querying GREEN-DB for annotating regulatory variants. For efficient calling of compound heterozygous variants, a table containing inheritance patterns of heterozygous variants was compiled. The resulting parquet database was then loaded into an apache spark cluster and queried via Rstudio/sparklyr.

## Splice site analysis

In addition to the well-established splicing prediction algorithms MaxEntScan [35] and SpliceAI [36], we used a novel algorithm, ALTSPLICE, which was developed at the University of Oxford, to investigate potential splice sites. ALTSPLICE uses the underlying DNA sequence to predict the impact of mutations on exon inclusion rates in expressed gene transcripts, in two stages. First, the location and usage frequency of splice donor and acceptor sites are predicted, using a multilayer convolutional neural network with 16,384 bases as input, with the predicted frequencies and their uncertainty represented as a Beta distribution. The true usage frequencies are estimated from GTEx read junction data, and also represented as a Beta distribution. A loss function compares these distributions and accounts for model mis-specification at particular *loci* and the existence of mis-mapped reads. Second, predicted splice junctions are considered within known transcripts and exon reading frames. The resulting transcripts ("primary transcripts") and their implied frameshifts are used to predict whether Nonsense Mediated Decay is triggered, resulting in transcript expression levels relative to the full set of primary transcripts. To assess whether a mutation affects transcript expression, the same procedure is run for the computationally mutated sequence and predicted relative expression levels compared on the transcript level. ALTSPLICE classifies sites into non-splicing, alternatively spliced and constitutively spliced sites, to quantify the degree of alternative splicing. This sets it apart from existing approaches that aim for a binary classification such as SpliceAI. In addition, the ALTSPLICE model is able to handle larger sequences (>16kb) than previous approaches such as SpliceAI which are limited to 10kb. It can be used to investigate the effect of SNVs on alternative splicing.

## Somatic variant analysis

*Identification of somatic mosaic variants*

We considered somatic variants in 2 cases - 005Kli001 and 065DSA001 where germline variants were not identified, and the phenotype suggested that somatic mosaic variants should be considered (e.g., overgrowth syndromes), and undertook additional targeted sequencing or WGS.

For 005Kli001, skin samples from affected haemangioma lesions were sequenced using targeted next generation sequencing. Initial NGS was carried out in OUFHT on Ion Torrent platform and then confirmed on MiSeq platform in the St George’s University Hospital NHS Foundation Trust, where patient had subsequently been referred. Polymerase chain reaction amplification of selected regions of the genome used the Swift Biosciences Accel-Amplicon® Plus 57G Pan-Cancer Profiling Panel; library preparation was performed according to manufacturer’s recommendations. Samples were labelled using unique ‘DNA barcodes’ and sequenced using an Illumina MiSeq. Raw sequence data was de-multiplexed using automated scripts and processed on DNA Nexus using MokaAMP; sequence alignment used BWA-MEM, pre-processing following GATK best practices and BAMclipper. Variant calling was conducted using VarScan and VarDict. QIAGEN Clinical Insight (QCI) Interpret software was used to annotate variants which were described according to Human Genome Variation Society nomenclature.

For 065DSA001, we investigated whether somatic variants contributed to the bone-specific phenotype observed. We compared WGS performed on DNA extracted from whole blood (germline) with WGS performed on DNA extracted from cell cultures derived from healthy bone tissue biopsy (healthy bone) and affected bone tissue biopsy (diseased bone). Germline and healthy bone samples were sequenced at 30X mean coverage while the diseased bone sample was sequenced at 120X mean coverage. Sequencing, alignment and QC were performed as described above and then we used Mutect2 v4.2.0.0 to detect somatic variants based on 3 different tumor-normal comparisons: healthy bone vs germline (HvsG), diseased bone vs germline (DvsG), and diseased bone vs healthy bone (DvsH). The analysis was performed according to GATK best practices for tumour-normal comparison, without using a panel of normals (cf. <https://gatk.broadinstitute.org/hc/en-us/articles/360035894731-Somatic-short-variant-discovery-SNVs-Indels->). We then compared PASS variants obtained from each comparison and selected as best candidates those present in both DvsH and DvsG analyses but absent in the HvsG one. This allowed us to focus on mutational events occurring specifically in the diseased tissue. Candidate variants were then annotated for gene consequences, population allele frequencies and impact prediction scores as described above for standard germline analysis.

The Genomics England gene panels for skeletal dysplasia and adult solid tumour cancer susceptibility (green and yellow genes) were used to prioritise genes known to be involved in relevant diseases.

## HPO analysis

The distribution of HPO terms per pedigree is shown in Additional file 1: Fig. S15. The heatmap showing pairwise similarities of HPO terms between pedigrees (Additional file 1: Fig. S16) indicates overlaps between major disease categories. For example, the ultra-rare disorders group, which comprises Kapur-Toriello Syndrome (KTS) and Fine Lubinsky Syndrome (FLS), overlaps with the musculoskeletal and neurological diseases groups. Since the musculoskeletal category contains patients with different forms of craniosynostosis, which are disorders of the skull, and since KTS and FLS exhibit both skeletal and neurological anomalies, this is to be expected. Similarly, there is overlap between the haematological, vascular and immunological disorders, although less overlap within the patients with immunological disorders than might have been anticipated.

## Bionano analysis

This procedure was performed at the Bionano Services Laboratory in San Diego, USA.

#### Ultra-high molecular weight DNA extraction and labelling

For each sample, a minimum of 650µl of whole peripheral blood was used to purify ultra-high molecular weight (UHMW) DNA using the Bionano Prep SP Frozen Human Blood DNA Isolation Protocol v2, following manufacturer’s instructions (Bionano Genomics, San Diego, USA). Briefly, white blood cells were counted, pelleted (2200g for 2min), and treated with LBB lysis buffer and proteinase K to release genomic DNA (gDNA). After inactivation of proteinase K by PMSF treatment, gDNA was bound to a paramagnetic disk, washed, and eluted in an appropriate buffer. UHMW DNA was left to homogenise at room temperature overnight. The following day, DNA molecules were labelled using the Direct Label and Stain (DLS) Protocol (Bionano Genomics, San Diego, USA). Briefly, 750ng of gDNA were labelled in presence of Direct Label Enzyme (DLE‑1) and DL-green fluorophores. After clean-up of the excess of DL-Green fluorophores and rapid digestion of the remaining DLE-1 enzyme by proteinase K, DNA backbone was counterstained overnight.

#### Data collection

Labelled UHMW gDNA solution (4-12ng/µl) was loaded on a Saphyr chip and imaged by the Saphyr instrument (Bionano Genomics, San Diego, USA). Saphyr chip was run at a target of 400Gbp to aim for 100X coverage.

#### Bionano rare variant analysis and structural variant calling

The *de novo* assembly and Variant Annotation Pipeline were executed on Bionano Solve software v3.7 [37]. Reporting and direct visualisation of structural variants was done on Bionano Access v1.7. DNA molecules were first aligned against the human reference genome GRCh38, and SVs and CNVs were identified based on discrepancies in the alignment between the sample and the reference. For each SV and CNV call, confidence scores were calculated and provided by Bionano Genomics [37]. For data filtering, the following recommended confidence scores were used: Insertion/Deletion= 0; Inversion= 0.7; Duplication= -1; Intra-fusion/Inter-translocation= 0.05; Copy Number= 0.99; Aneuploidy= 0.95. Events detected by *de novo* assembly were subsequently filtered against the OGM control database. Additional filtering by pairwise comparison (dual analysis) was performed to easily distinguish inherited and non-inherited variants.

## Functional Studies

#### Minigene assay for *SLC34A1* splice site variant

*SLC34A1* minigene constructs were designed to comprise two joined segments generated by PCR amplification from individual genomic DNA samples: a 384 bp fragment spanning the exon 10-intron 11 junction (using P1 and P2 primers), and a 509 or 698 bp fragment spanning the intron 10-exon 11 boundary through to exon 12 (using P3 and P4 primers). The constructs were designed to span the region containing the 189bp deletion within intron 10, in order to assess any effect of the deletion on splicing. Three constructs were made: a paternal construct with a c.1175-3C and short intron 10; a maternal construct with a c.1175-3A and full length intron 10; and a wild-type (WT) construct with a c.1175-3C and full length intron 10. Constructs were cloned into a pBI-CMV2 expression vector. Following transfection of HEK293 cells with the three expression vectors, RNA was harvested after 24h and reverse transcription performed to generate cDNA. PCR analysis of cDNA using *SLC34A1*-specific primers was performed, and products were run in an agarose gel in order to identify splice events. Bands were excised from the gel in order to allow DNA sequencing.

The primers that were used for the PCR of the cDNA (Additional file 1: Fig. S20 panel F), primers P1 and P4, were:

P1 (5’ to 3’) GGATCCCTGGTGCTGCTGTGC

P4 (5’ to 3’) GTCGACAAACGCGTGGACAGCTTCTC

These generated a 344bp fragment when splicing was wild type.

#### RT-PCR assay for *SEC23B* splice site variant

To validate the splice site variant in *SEC23B* (ENST00000650089.1: c.1512-16A>G), Trizol treated blood samples (1ml per 5mls peripheral blood) were snap-frozen and stored at minus 70. RNA was extracted using Zymo Direct-Zol Mini prep Plus kit (Zymo Research R2070). 1ug of RNA was treated with DNAse1 (Sigma 4716728001) for 30mins at 37^o^C and the reaction quenched using 1ul 50mM EDTA. cDNA synthesis was performed using the High-Capacity Reverse Transcription Kit (*ThermoFisher Scientific 4368814*). PCR was conducted using locus specific primers and the resulting PCR products were Sanger sequenced.

#### Minigene assay for *ABCB4* splice site variant

*ABCB4* exon 3 and flanking intronic sequences encompassing the *ABCB4* variant (ENST00000649586.2:c.135+26A>G) were amplified from the genomic DNA of the patient by PCR using forward and reverse primers containing restriction sites for Sall and XbaI, respectively. The fragments with the wild type and mutant alleles were cloned into the RHCglo vector and then transformed into E. coli TOP10 competent cells (Invitrogen, USA), followed by screening by Sanger sequencing.

COS7 cells were cultured in DMEM medium containing 10% fetal bovine serum, 1% penicillin and streptomycin and 1% L-glutamine. After twenty-four hours, 1.25 μg of the minigene constructs containing the wildtype or mutant *ABCB4* fragments were transfected into COS7 cells using Lipofectamine 3000 (Invitrogen, USA) according to manufacturer`s recommendations. Cells were collected after 48 hours and total RNA was isolated. RT-PCR was performed using the downstream primer (TNIE4) additionally to increase the specificity of the reverse transcription. The PCR amplifications were performed from cDNA using the upstream and downstream primers (RSV5U and TNIE4) of the RHCglo vector. The wildtype and mutant products were then visualized using Bioanalyzer and evaluated by Sanger sequencing.

RHCglo was a gift from Thomas Cooper (Addgene plasmid #80169, http://n2t.net/addgene:80169 ; RRID; Addgene_80169)

ABCB4 Cloning primers (5’-3’):

ABCB4-V2_SalI

ACGCGTCGACGTCGGCCATAGCGGCCGCGGAAACCACTCTCCACAATCCTTCA

ABCB4-V2_XbaI

GCTCTAGAGCGCCAAACTTATACAGCACAGGT

RHCglo Vector Backbone Primers (5’-3’):

Upstream Primer:

RSV5U Primer: CATTCACCACATTGGTGTGC

Downstream Primer:

TNIE4 Primer: AGGTGCTGCCGCCGGGCGGTGGCTG

#### Minigene assay for *BMP4* variant

The region of *BMP4* flanking the variant of interest (ENST00000245451.9:c.370+441G>A) were amplified from genomic DNA using the following primers and cloned into a pSPL3 exon trapping vector, transfected into HEK293T cells.

BMP4 cloning primers (5’-3’):

hu_BMP4 Ex 3-4 XhoI_F: aattctcgagGGCTCTGAACCAACTGGAAG

hu_BMP4 Ex 3-4 BamHI_R: attggatccAGGTCAAGGTGAATGTTTAGGG

Sequencing primer (5’-3’):

BMP4 cDNA Ex 4: GCGGCACCCACATCCCTCTACTAC

#### RT-PCR assay for *VDAC2* splice site variant

RNA was obtained from fibroblasts from the patient and from two unrelated controls. RT-PCR was performed with primers VDAC2_7F (ACCTTCTCACCAAACACAGGA) and VDAC2_11R (CCCATCTACCAGAGCAGAGAG). Duplicate reactions without reverse transcriptase enzyme (RT-) were run in parallel in order to discard genomic DNA contamination. Products were run in agarose gels for visualization and Sanger sequencing was performed in both directions in order to discriminate splicing changes.

#### RT-PCR assay for *RBPJ* variant

RT-PCR was performed on blood RNA (PaxGene) using a set of exonic primers below.  RT reactions were carried out with quantiTECT (Qiagen)

RBPJ-5F     CAAGAGTCTCAACCGTGTGC

RBPJ-8R     TCGGACTGTGAATTCTTCTCCT

#### Expression of *ALG13* wild type and mutant

A plasmid containing the wild type *ALG13* tagged ORF clone was purchased from (RG215034, NM_001099922, Origene). The S875N mutation was introduced by site-directed mutagenesis and the plasmid sequenced to verify the mutation.

HEK293 T cells were seeded at 3x10^5^ per well of a 6-well plate and transfected the following day with a mixture of 3µg ALG13 wild type or mutant plasmid DNA (RG215034, NM_001099922, Origene), 1.25 µl 20% glucose, and 1.5 µl of 5.625 µg/µl PEI (Sigma-Aldrich). The mixture was incubated at RT for 10 minutes and added to 2 ml DMEM containing glutamine and pyruvate with 10% FCS and PSA. The transfection media was removed 16 hours later, and fresh media was added.

At 48 hours post-transfection, the transfected cells were lysed at 4^°^C for 1 hour in 500 µl lysis buffer (10 mM Tris-HCl, 100 mM NaCl. I mM EDTA, 1% Triton-X, pH7.5) with 1:100 protease inhibitor cocktail (Sigma_Aldrich. Cat#P8340) per well of 6-well plate. The whole cell extract was spun at 13,000 g for 5 minutes to pellet the cell debris and the supernatant containing the ALG13 was retained.

15 µl of each protein sample was loaded onto a NuPAGE 4-12% Bis-Tris gel (Invitrogen). The gel was run at 150 V for 30 minutes using Mops SDS running buffer (Invitrogen). Five microliters of Prime Strep Prestained Broad Range Protein Ladder (BioLegend), and/or 5 µl of SeeBlue Plus2 Prestained Standard (Invitrogen) was also loaded. Protein was transferred onto nitrocellulose (GE Bioscience) using an Novex Mini-cell (Invitrogen) running at 30 V for 2 hours with NuPAGE Transfer buffer (Novex) supplemented with 20% methanol. The blot was washed with 1x PBS containing 0.1% Tween (PBST), blocked with 10% Marvel in PBST for 1 hour, and washed 3 times with PBST. Anti-ALG13 primary antibody (67283-1-Ig, proteintech) was diluted 1:1000 in PBST containing 10% Marvel and incubated at 4^°^C overnight with shaking. The blot was then washed 3 times with PBST, and a polyclonal goat anti-mouse secondary antibody (Dako) was added (diluted 1:1000 in PBST containing 10% Marvel). The blot was incubated for 1 hour at room temperature, and washed 3 times with PBST. Two millilitres of ECL solution (GE Healthcare) were prepared and applied to the blot as per manufacturer’s instructions. ALG13 bands were visualised by exposing them to photographic film for 5 min.

## Supplementary Figures


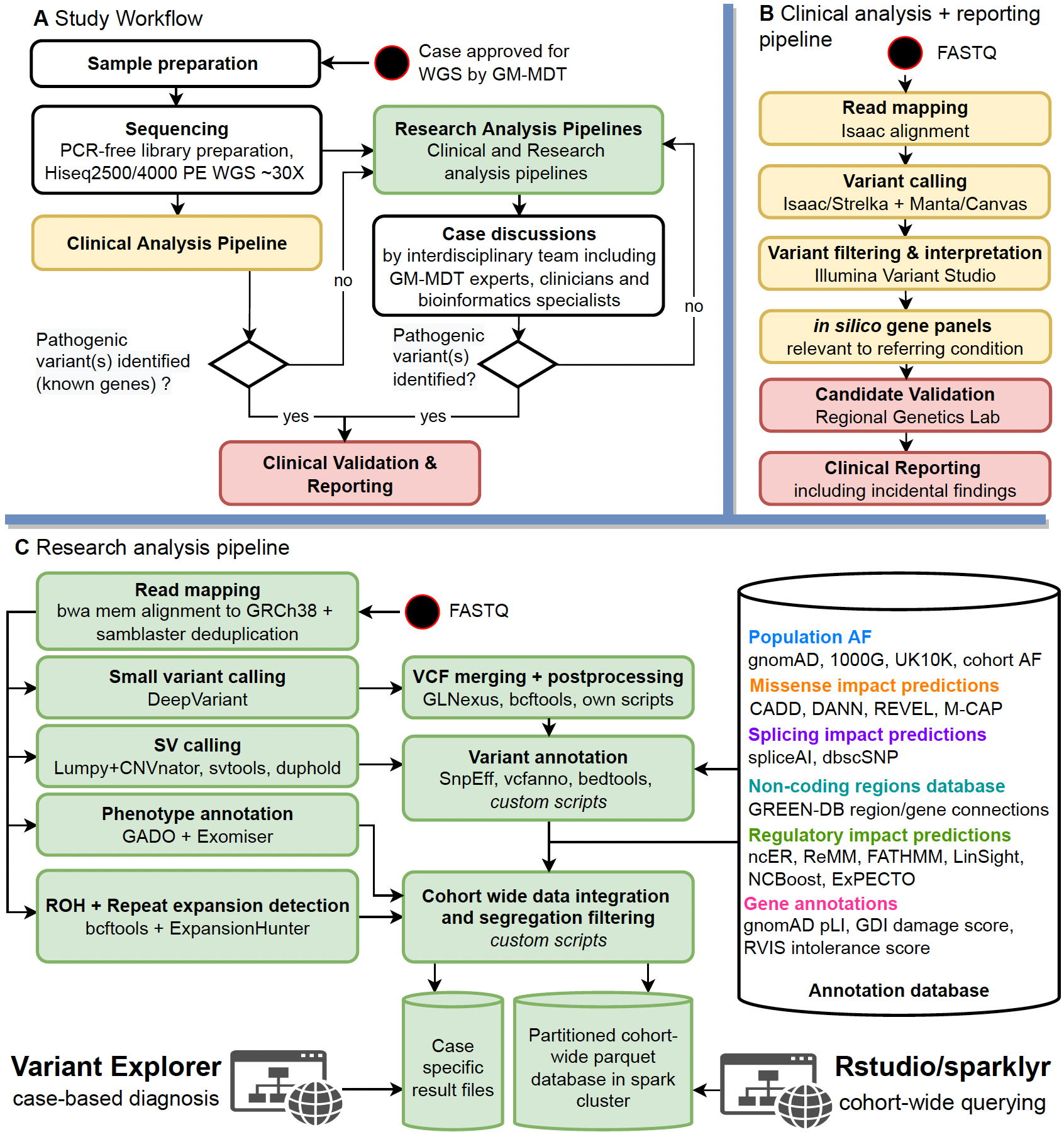
 **Fig. S1**: Study overview depicting core steps of the overall workflow (A), the clinical analysis and reporting pipeline (B) and the research analysis pipeline (C). **A** Case selection and referral was done by the Oxford Genomic Medicine Multidisciplinary Team (GM-MDT), a detailed description of this process is provided in Ormondroyd et al [38]. Selected samples were whole-genome sequenced and analysed by a clinical (yellow) as well as a research pipeline (green). Identified pathogenic candidate variants were validated and reported back to referring clinicians. Unsolved cases were iteratively investigated by a research pipeline incorporating the latest methods for *in silico* analysis of WGS data. Resulting novel disease candidate variants were regularly discussed by an interdisciplinary expert team and either rejected or forwarded to (functional) validation. **B** Our clinical analysis pipeline, based on the Illumina software stack, was operated by Oxford Molecular Diagnostic Centre (MDC) in collaboration with the Oxford Regional Genetics Laboratories (RGL). Gene panels relevant to the referring condition, as well as the ACMG 56 gene panel, were applied to the identified variants *in silico*. Emerging candidates and additional findings were validated by the RGL and finally reported back to the referring clinicians. **C** Our research pipeline evolved over the project lifetime as novel analysis methods became available. To enable a unified, cohort-wide analysis, we ultimately applied the depicted pipeline to all 300 samples. Briefly, deduplicated reads were mapped to GRCh38, small variants were called with DeepVariant and structural variants were called by a Lumpy+CNVnator based pipeline. Phenotype-related genes were identified with GADO and Exomiser based on HPO profiles curated by referring clinicians. Regions of homozygosity (ROH) and repeat expansions were called by respective tools. Genomic variants were comprehensively annotated by a large range of resources, including allele frequency databases, deleteriousness scores, regulatory regions and splicing impact prediction scores. Finally, all data were integrated into individual case result files as well as into one large parquet database hosted by a distributed Apache Spark cluster. The former was used to support case-based analyses by research analysts, the latter to conduct cohort-wide analyses.


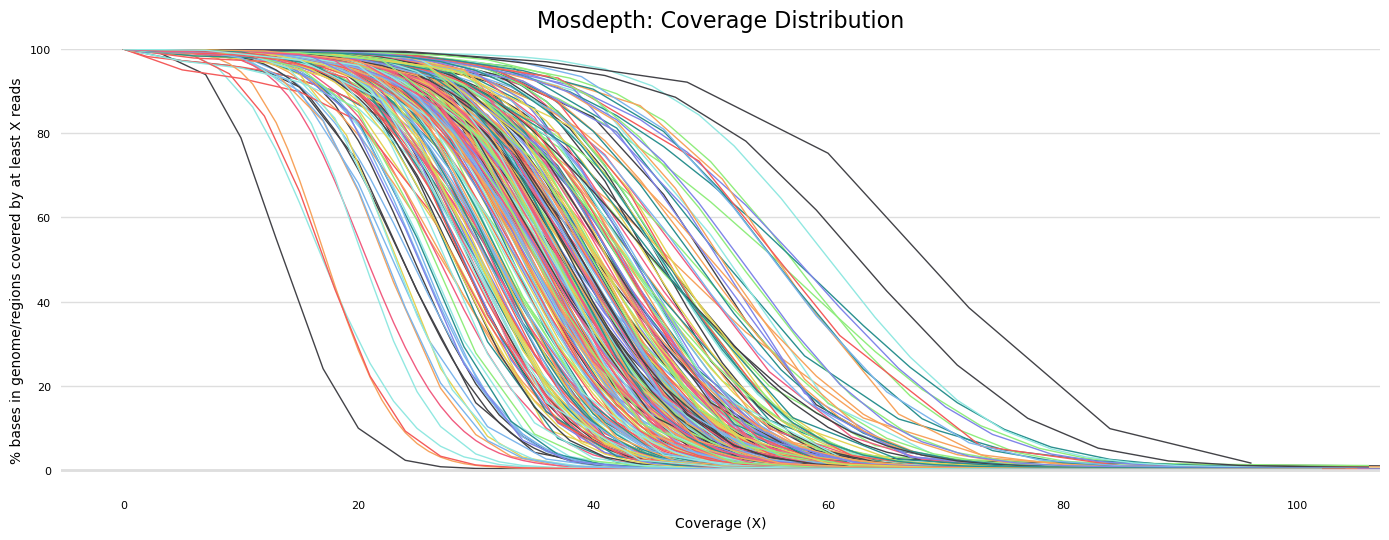


**Fig. S2**: Cumulative coverage distribution across the genome as calculated by mosdepth [5] for all samples included in the study.

**A B**

**
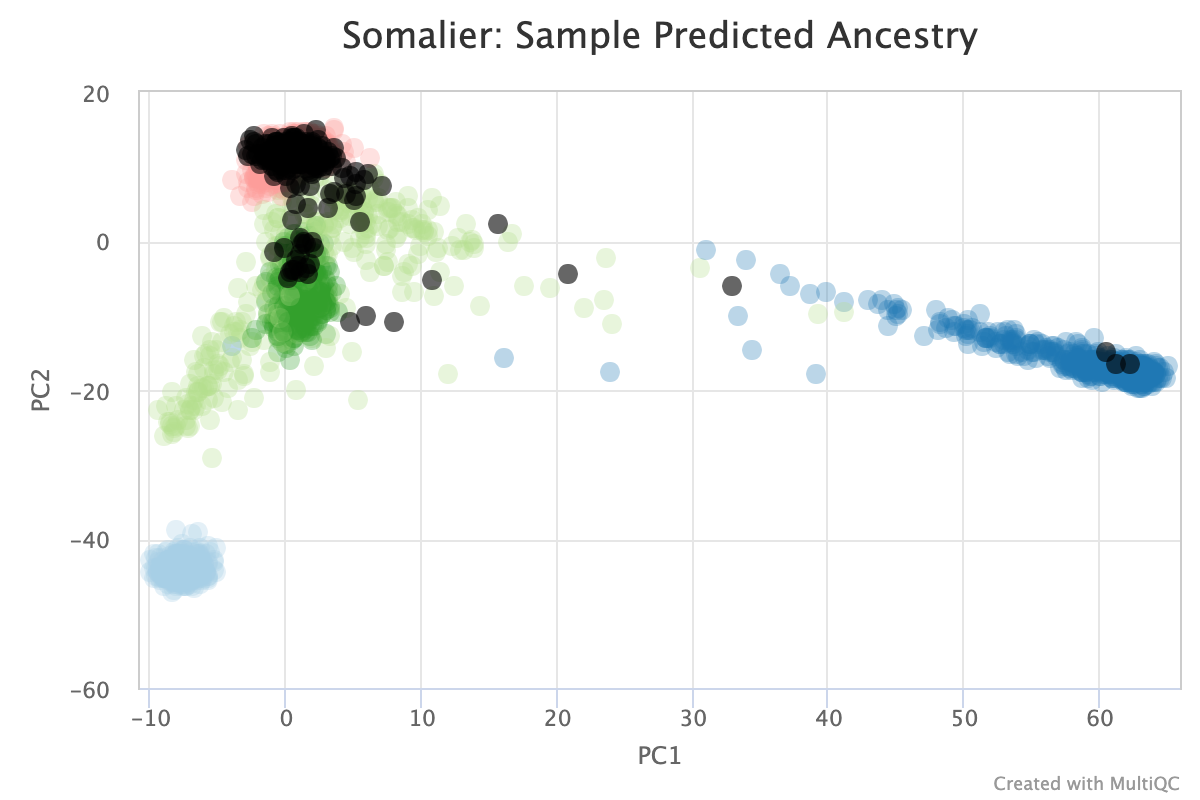

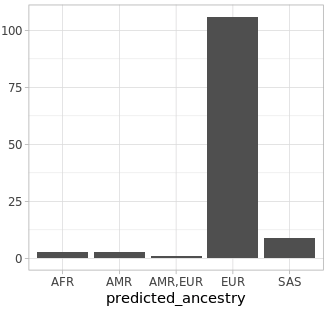
**

**Fig. S3**: Predicted ancestry of cohort samples. **A** PCA plot depicting estimated ancestry for cohort samples (black dots) against 1000 Genomes background samples. Blue: AFR, light-blue: EAS, green: SAS, light-green: AMR, red: EUR. Figure created with Somalier [6] and multiqc [39]. **B** Barplot showing number of pedigrees per ancestry. One pedigree was of mixed European/American ancestry.


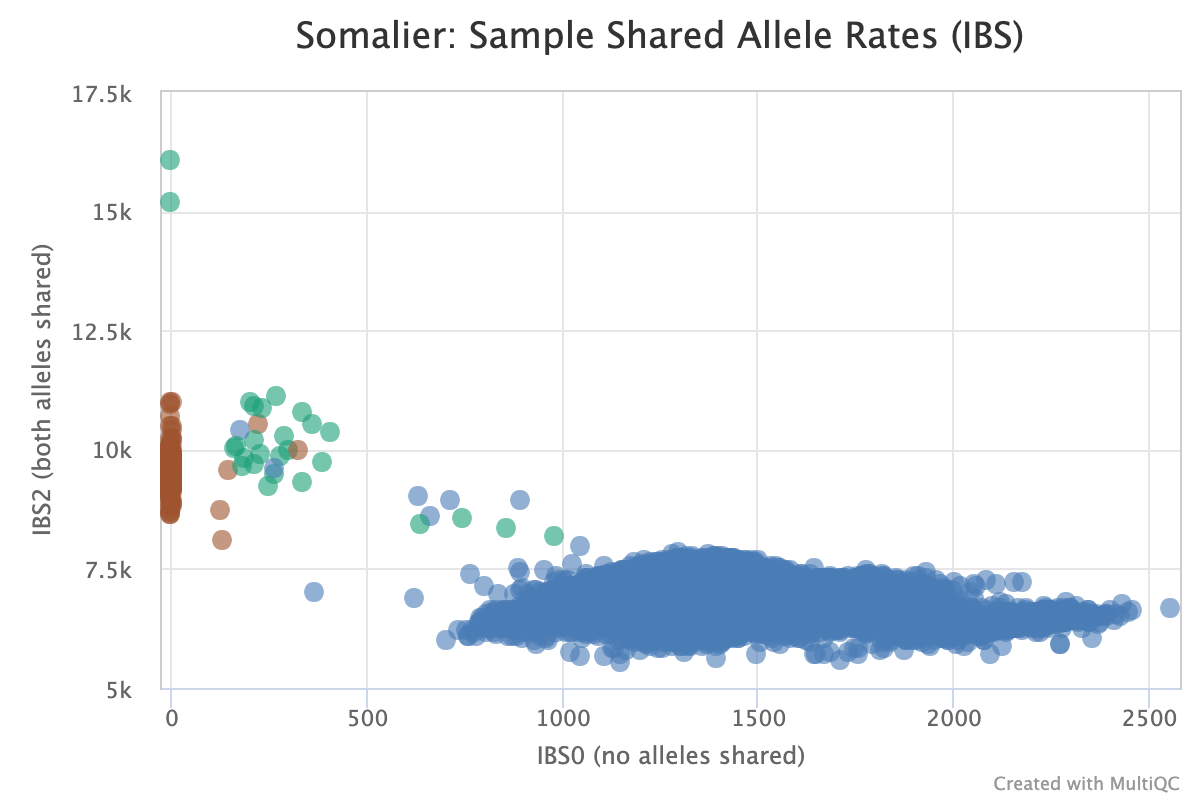

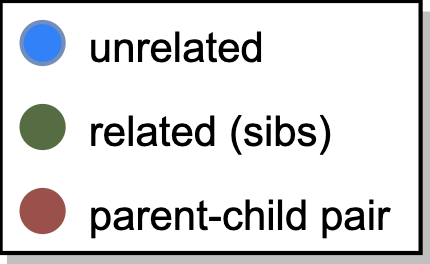


**Fig. S4**: Predicted relatedness (shared allele rates) between cohort sample pairs. Each point represents a sample pair and is coloured by degree of expected relatedness: blue: unrelated, green: related (sibs), brown: parent-child pairs. Although most dots fall into the expected area, we also observed (unexpected) cases of consanguinity as well as (expected) cases with very high relatedness requiring special attention in the analysis. The two green dots in the upper-left corner, for example, correspond to two monozygotic twin pairs. Figure created with Somalier [6] and multiqc [39].


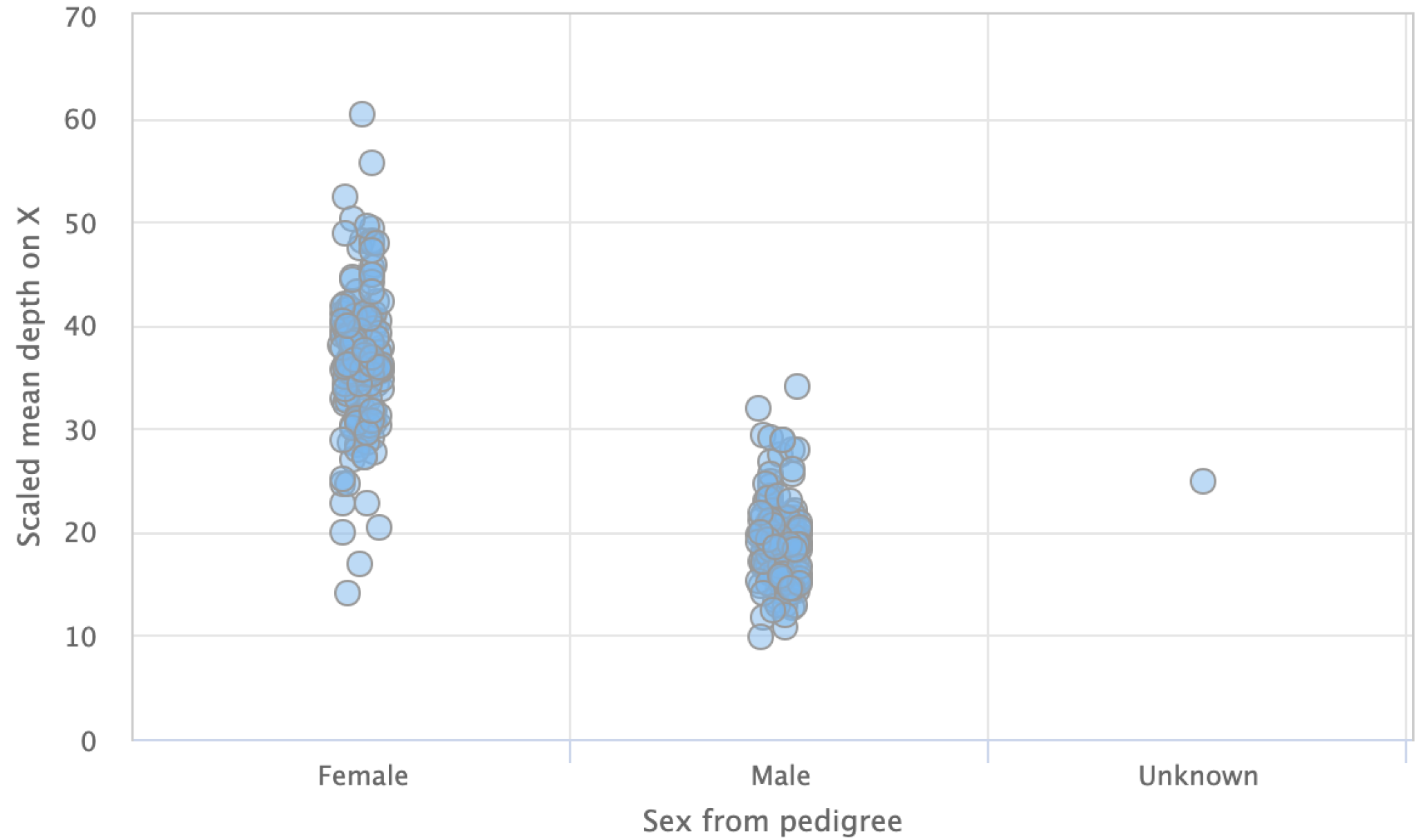


**Fig. S5**: Mean sequencing depth on X-chromosome per configured sample sex.


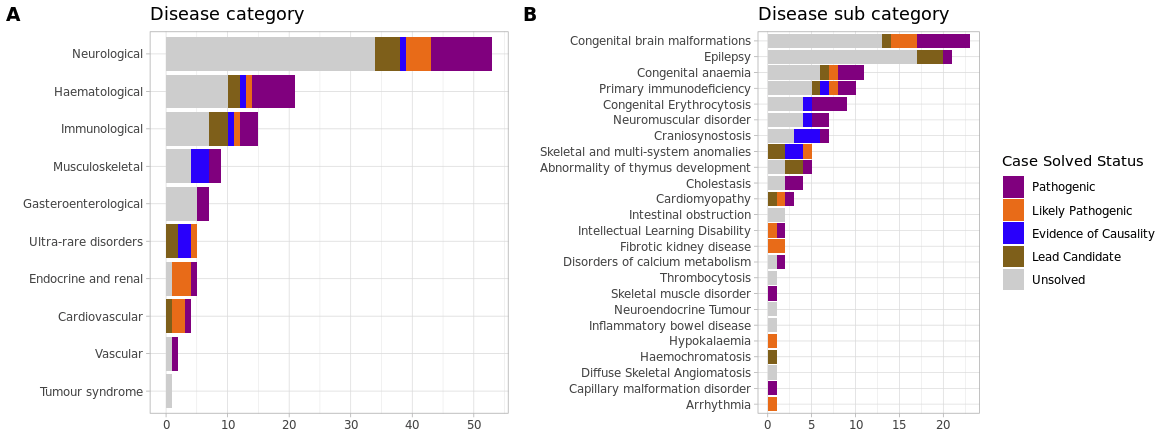


**Fig. S6**: Number of pedigrees per disease (sub) category as assigned by referring clinicians.


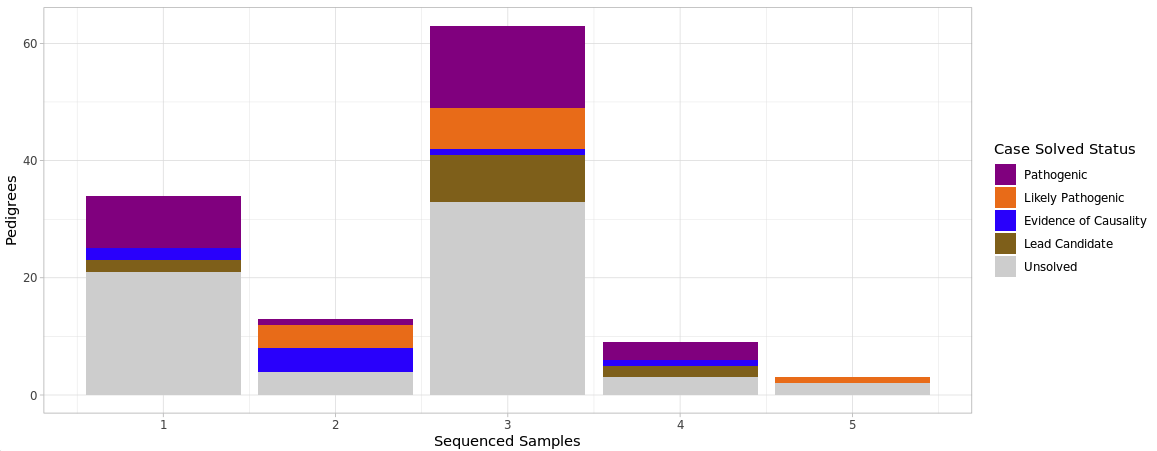


**Fig. S7**: Size of pedigrees analysed in the cohort. We stratified the solved status by size of the pedigree, indicated as the number of sequenced individuals per pedigree. Our cohort contains 300 samples in 122 pedigrees, including 63 trios. Of these, 61 were canonical trios (parent-child), while 2 included the proband, one parent and another relative.


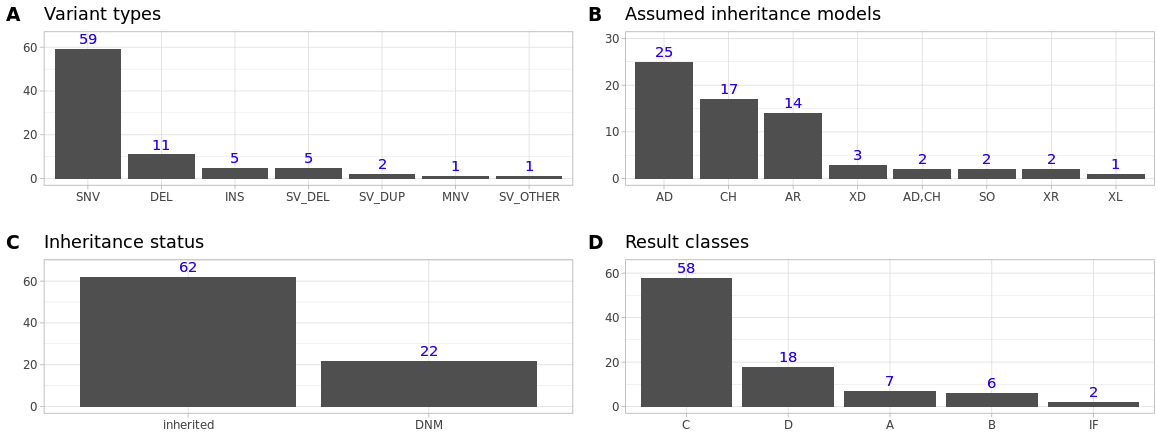


**Fig. S8**: Candidate variant statistics. **A** Variant type for all unique, putative pathogenic candidate variants (Additional file 3: Table S7). **B** Assumed inheritance model per pedigree with candidate variants. AD: autosomal dominant, CH: compound heterozygous, AR: autosomal recessive, XD: X-linked dominant, SO: somatic, XL: assumed X-linked. Multiple models possible if more than one candidate variant for a pedigree. **C** Inheritance status of all unique candidate variants. DNM: *de-novo* mutation. **D** Result class per candidate variant. A: likely novel gene, B: novel (mechanism) for phenotype, C: known gene for phenotype, D: candidate gene, IF: incidental finding. See Supplemental Table S3 for further description of the classification schema. Multiple classes possible if more than one candidate variant for a pedigree.

**
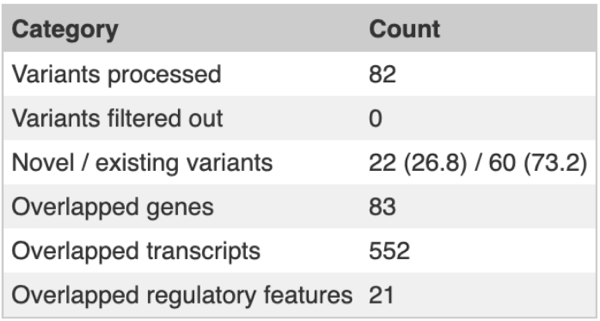

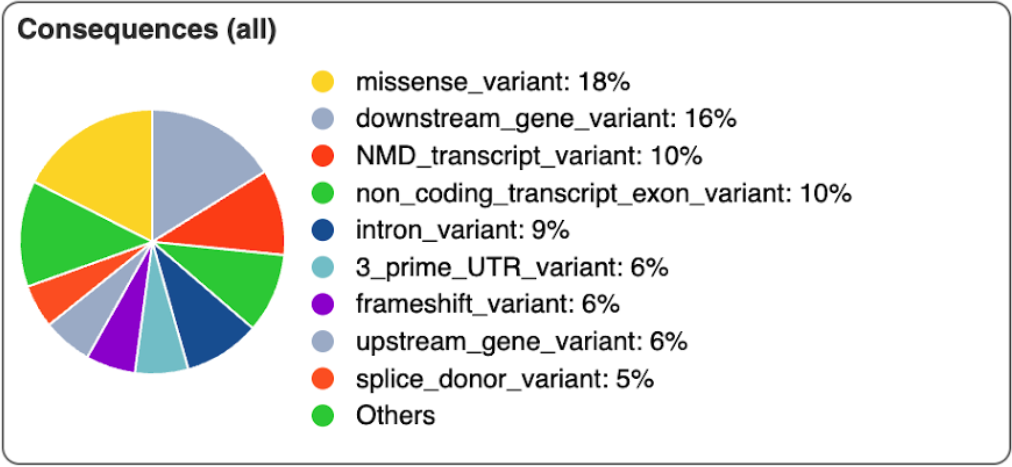
**

**
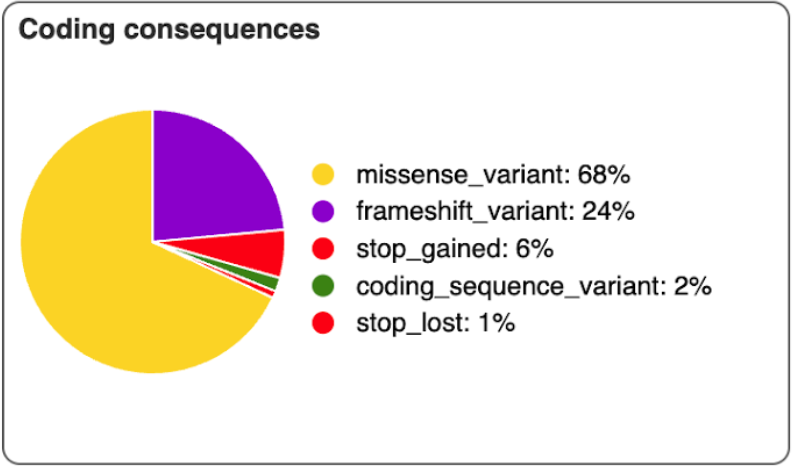
**

**Fig. S9**: VEP annotation statistics for all small candidate variants (excluding 1 MNV and 2 intergenic variants).

**
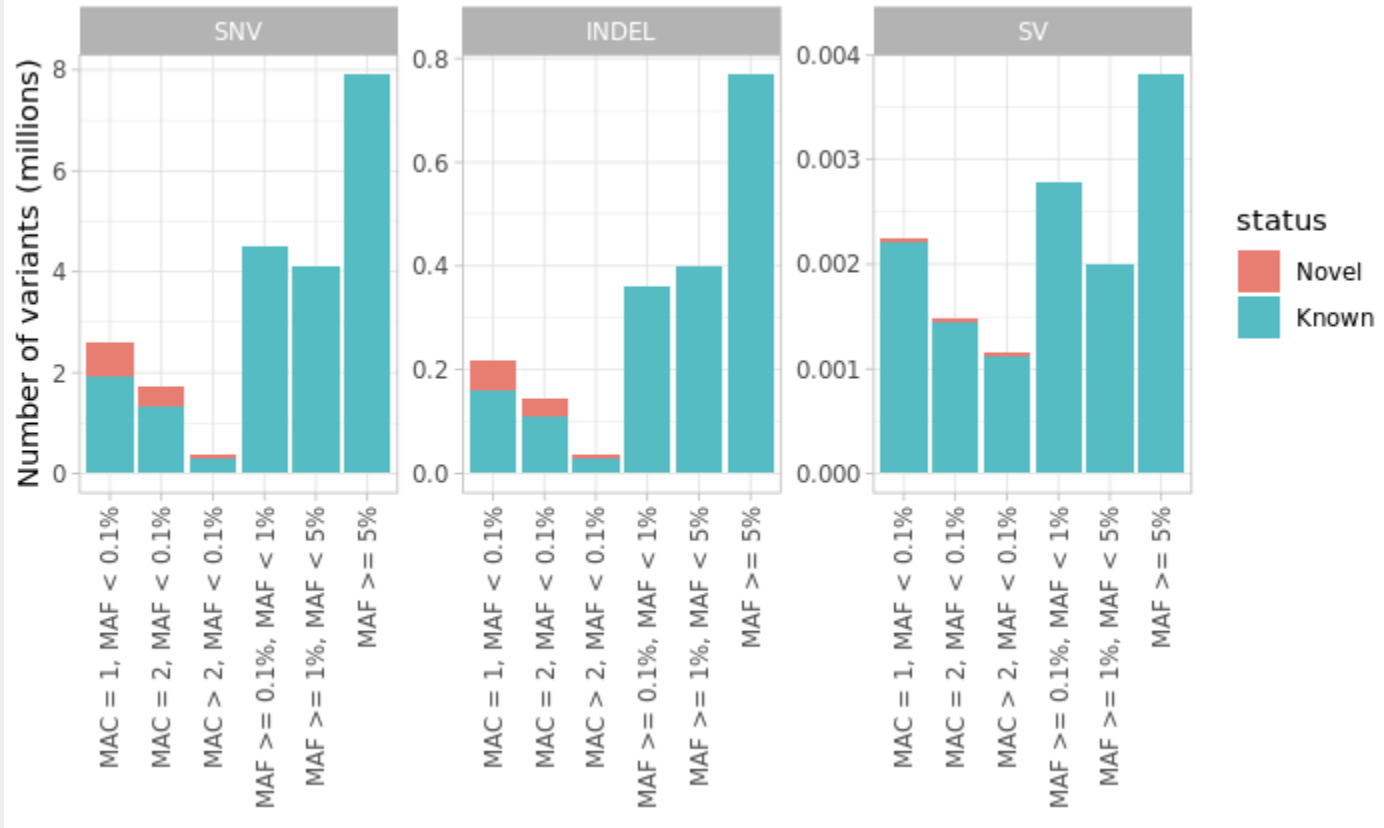
**

**Fig. S10**: Minor allele frequencies. Minor allele frequency (MAF) distributions of variants called in the cohort (n = 300), stratified by type (SNV, INDEL, SV). The SV category contains large deletions, insertions, duplications, inversions and CNV calls. Small variants are labelled novel if not contained in any of the 1000 Genomes, ExAC, UK10K, WGS500 [40], gnomAD and gnomAD_exome datasets. The *NA* category contains calls with missing population allele frequencies and MAC>2. SVs are labelled novel if not contained in the 1000 Genomes, gnomAD and IMH datasets. MAC: minor allele count.


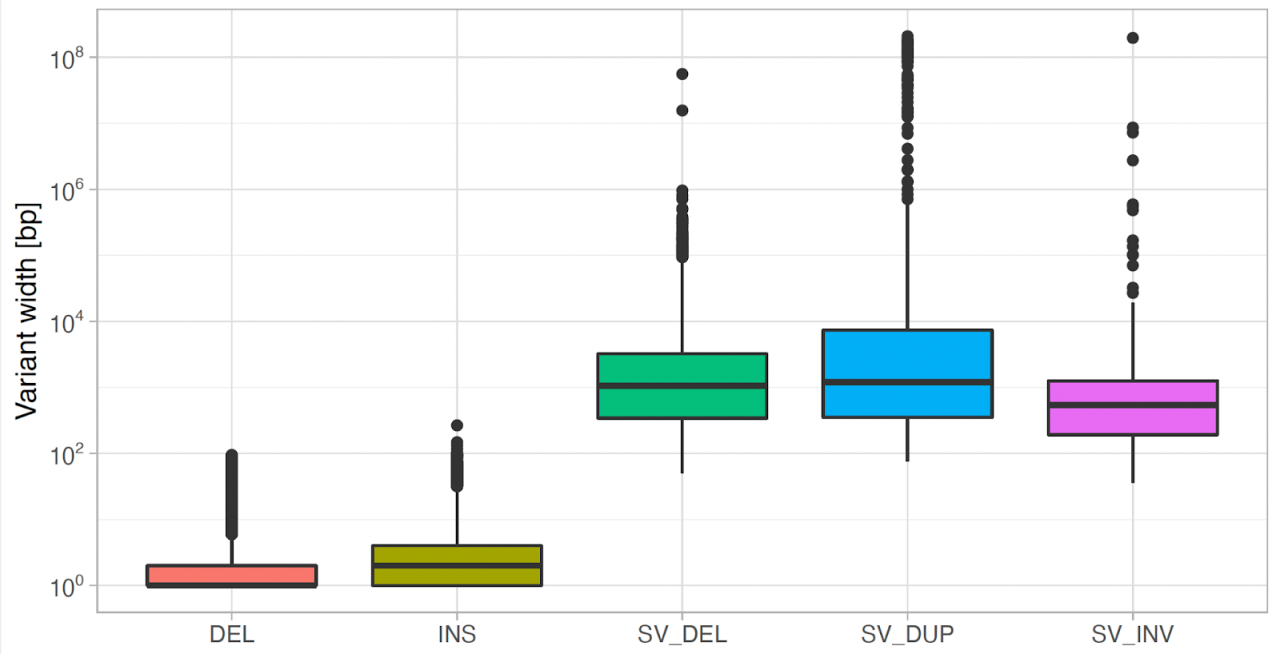


**Fig. S11**: Variant size distributions. DEL, INS: small deletions/insertions called by DeepVariant, SV_DEL, SV_DUP, SV_INV: large deletions, duplications and inversions called by our structural variation calling pipeline.


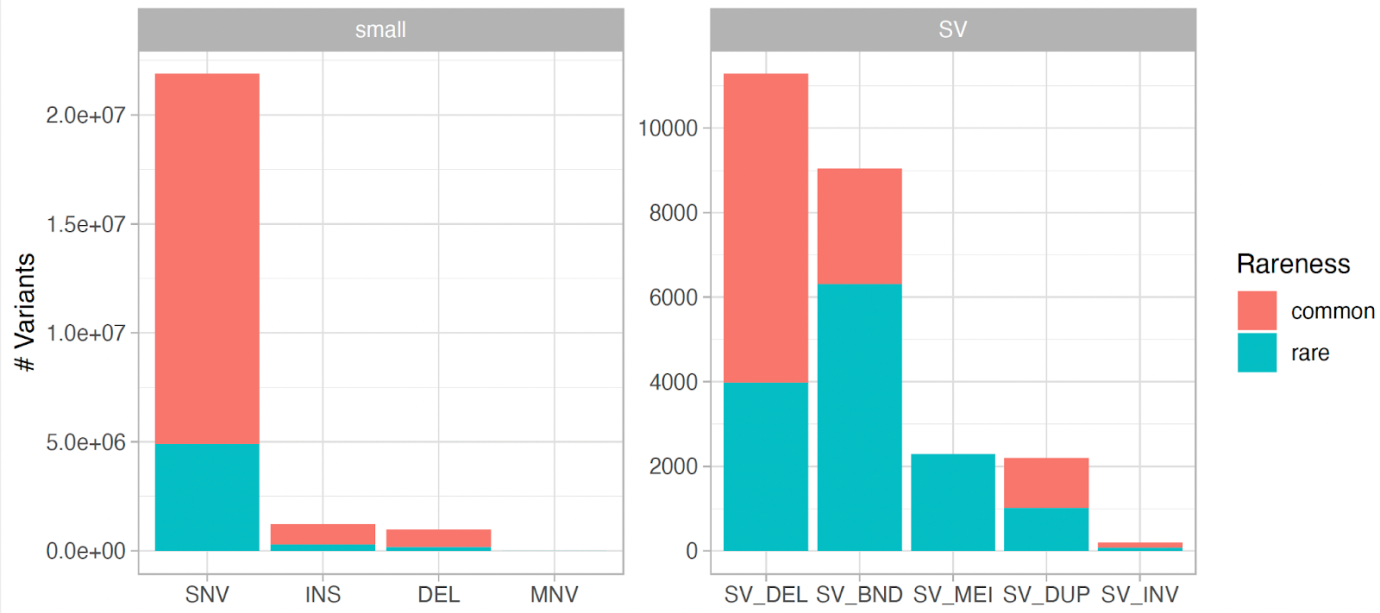


**Fig. S12**: Number of variants per type. SNV: single-nucleotide variants, INS: small insertions, DEL: small deletions, MNV: multi-nucleotide variants (n=68), SV_DEL: large deletions, SV_BND: generic breakends, SV_MEI: mobile element insertions, SV_DUP: large duplications; SV_INV: large inversions. Variants were considered to be rare if their maximum global population allele frequency (calculated over multiple databases) if any was <1%.


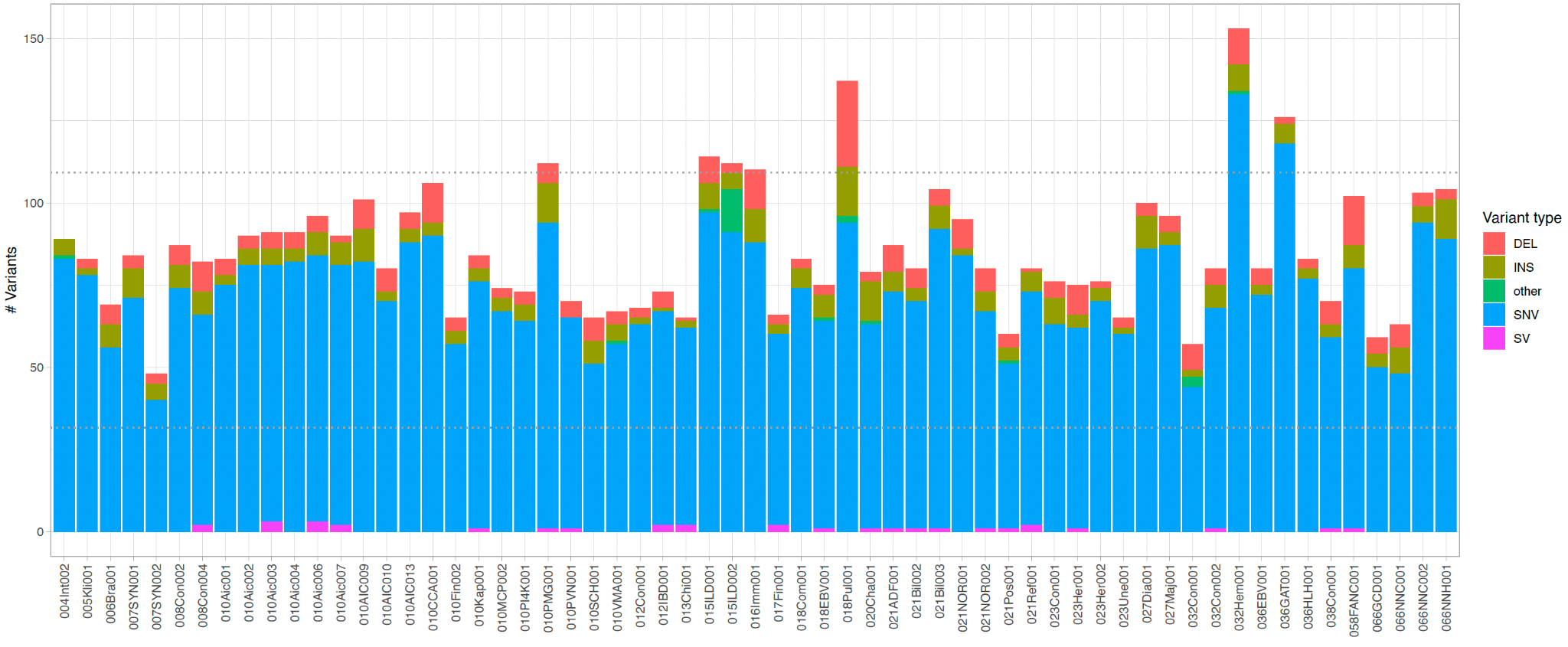


**Fig. S13**: *De novo* variants in affected samples per canonical trio pedigree (n=61) excluding sex chromosomes. Small variant candidates were filtered for segregation, parents genotype quality (GQ≥20), minimum sequencing depth (DP≥12), allelic depth of alt-allele in affected sample (AD≥4), no alternate allele in parents and allelic balance in affected samples (0.25<AB<0.75). Large deletions and duplications were filtered for segregation and variant probability (Phred-scaled probability that this site is variant, SQ≥20). Generic breakends (BND) were filtered for segregation, SQ and allele quality (AQ≥5). All variants were filtered for maximum population AF<1% and maximum cohort frequency<10% and we excluded candidates that were found in any of the unaffected samples. SNV: single nucleotide variants; DEL, INS: small deletions/insertions; SV: large deletions, insertions, duplications; other: all other SV types. Dotted grey lines indicate expected *de novo* mutation rates at $1.1-3 \times{10}^{-8}$ per nucleotide per generation.


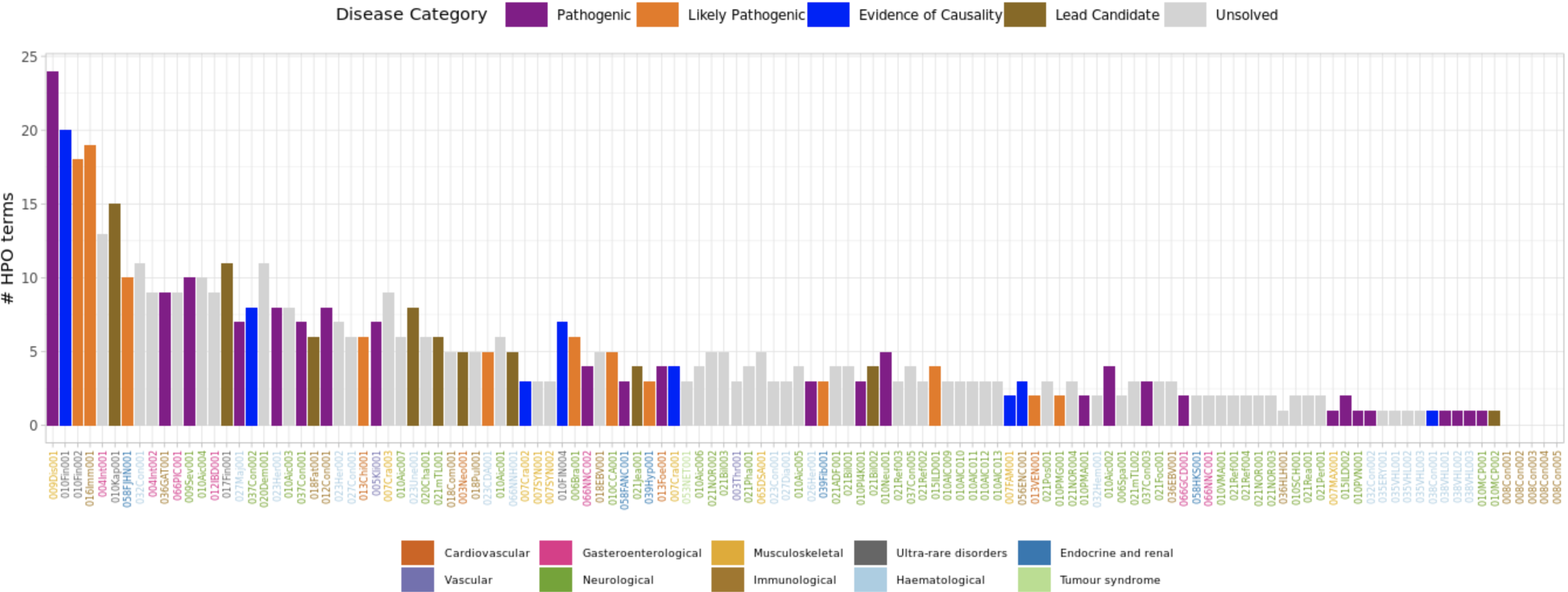


**Fig. S14:**  Number of HPO terms per pedigree. Bars are coloured by solved status and sorted by decreasing information content (IC) of the respective HPO profile as calculated with PyHPO v3.1.2 (https://github.com/Centogene/pyhpo). IC and profile size are highly correlated (Pearson R=0.97). Pedigree labels are coloured by disease category. HPO terms were manually annotated by clinicians or researchers based on phenotypic descriptions. Most pedigrees were annotated with at least one HPO term (117/122=96%) and mean number of terms per pedigree was 4.7. Many HPO terms were assigned to multiple pedigrees (107/301=36%) and the most common HPO term in our cohort, *Seizures* (HP:0001250), was assigned to 33 pedigrees.


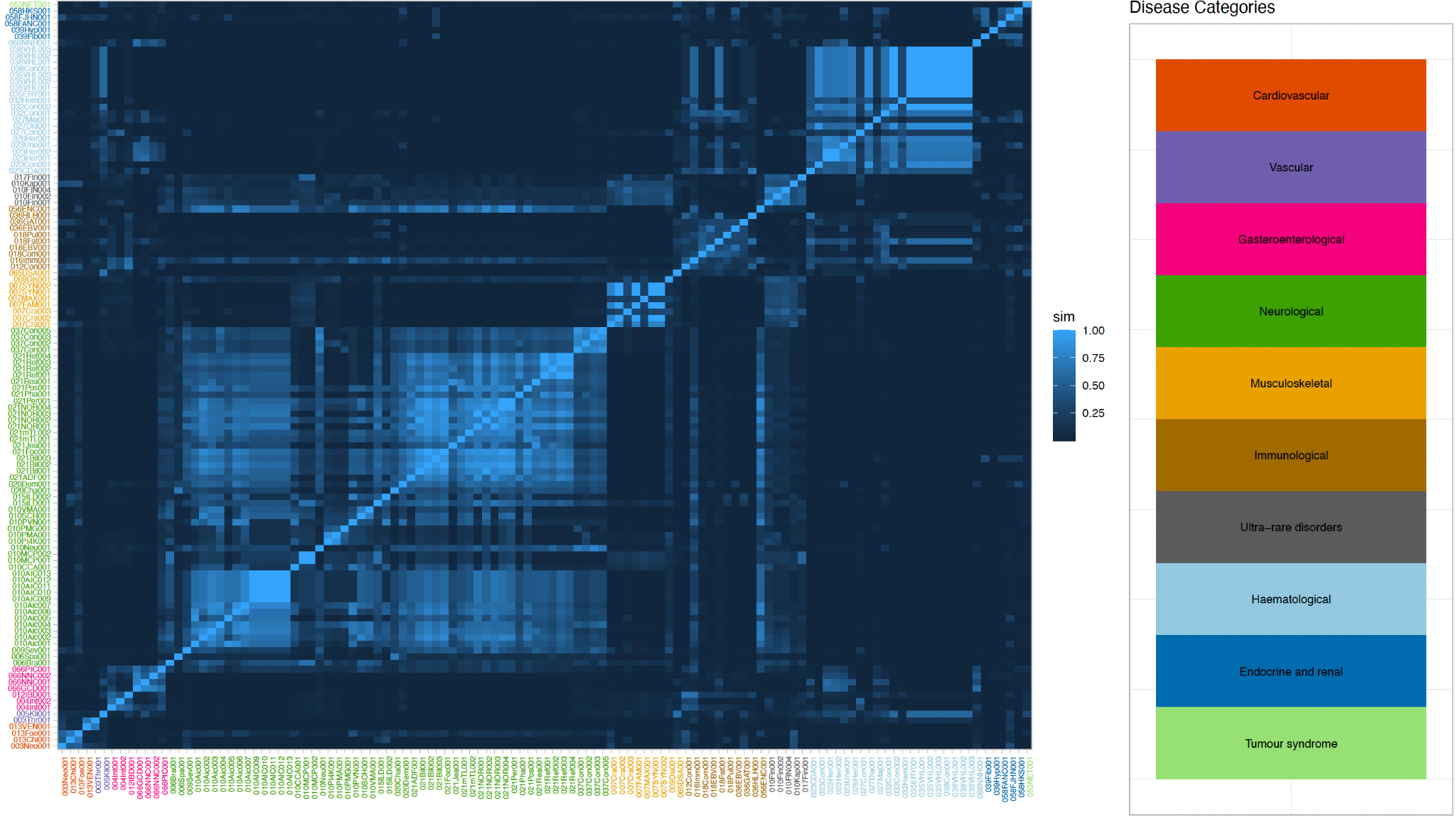


**Fig. S15:** HPO profile similarities. Heatmap showing the pairwise HPO profile similarities between pedigrees as calculated with PyHPO v3.1.2 (https://github.com/Centogene/pyhpo) according to Deng et al [41]. Higher values mean more similarity. Pedigree labels are coloured by disease category, pedigrees without associated HPO terms were omitted.


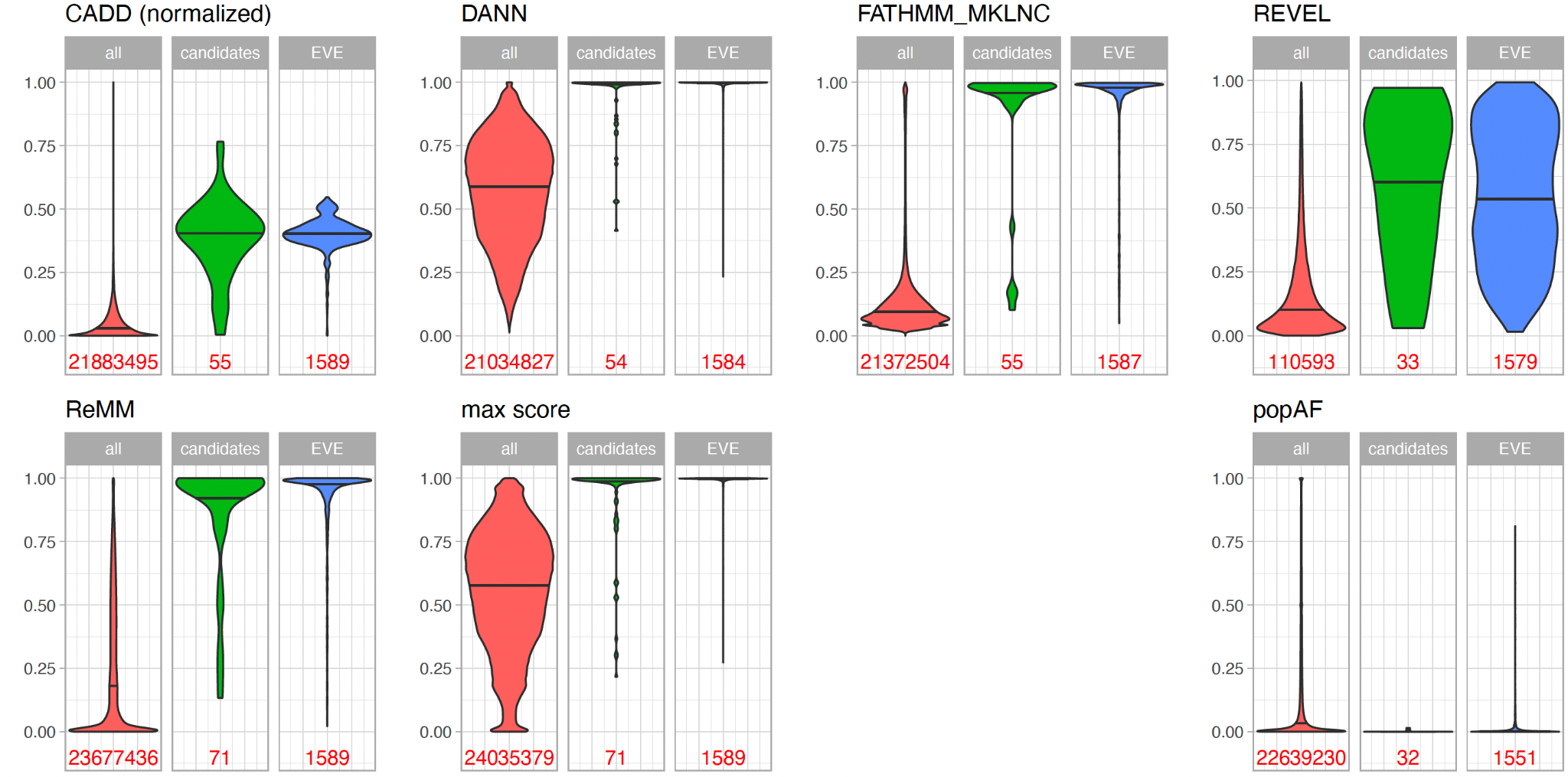
**Fig. S16**: Score distributions for all, candidate and EVE pathogenic small variants. The panels show violin plots of various deleteriousness scores and a maximum score calculated from those as well as population allele frequencies. CADD phred scores were normalised to the maximum number found in our cohort (64). Red distributions were calculated from all small variants with associated values, green distributions from all small candidate variants in Table 2, blue distributions from all small variants considered as pathogenic (EVE_classes_75_pct_retained_BPU == 'Pathogenic') by EVE (evolutionary model of variant effect) [42]. Note that 11, 4 and 1 of our candidate variants were classified as pathogenic, uncertain and benign respectively by EVE while there was no data for the remainder. Horizontal lines indicate median of distributions, number of observations are plotted below violin plots in red colour, max score: maximum of all shown, normalised deleteriousness scores; popAF: maximum global allele frequency.


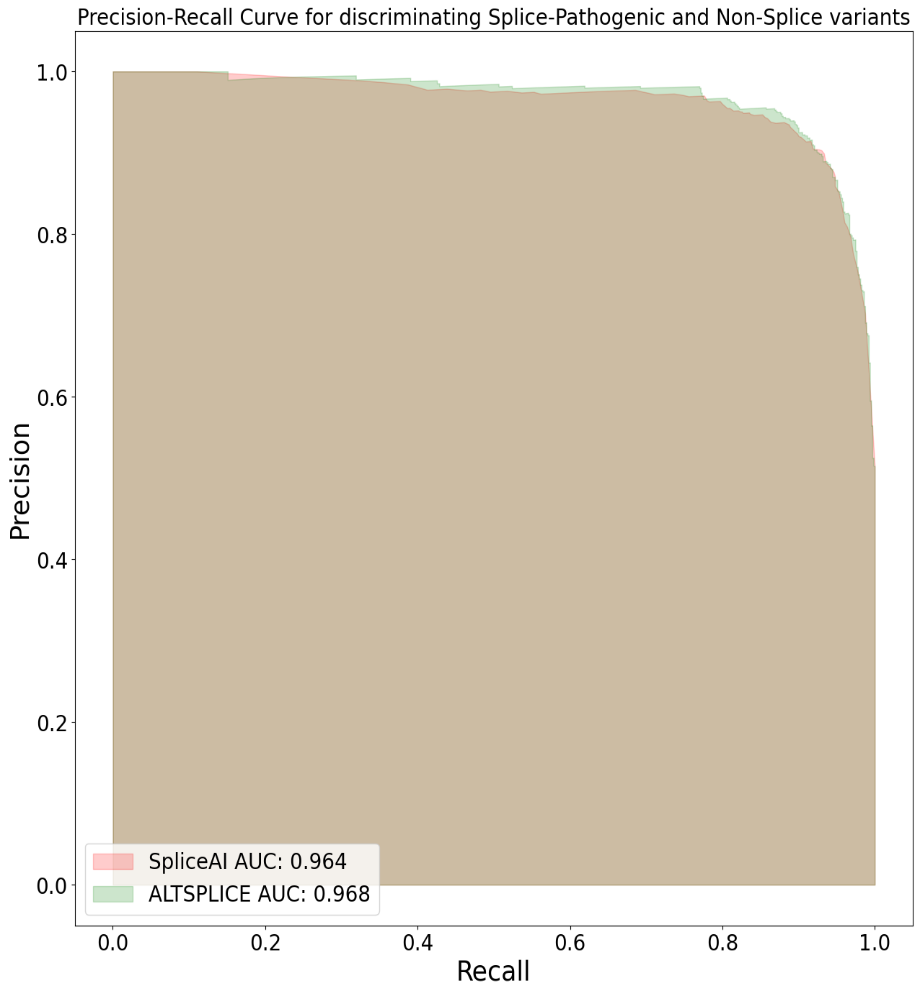

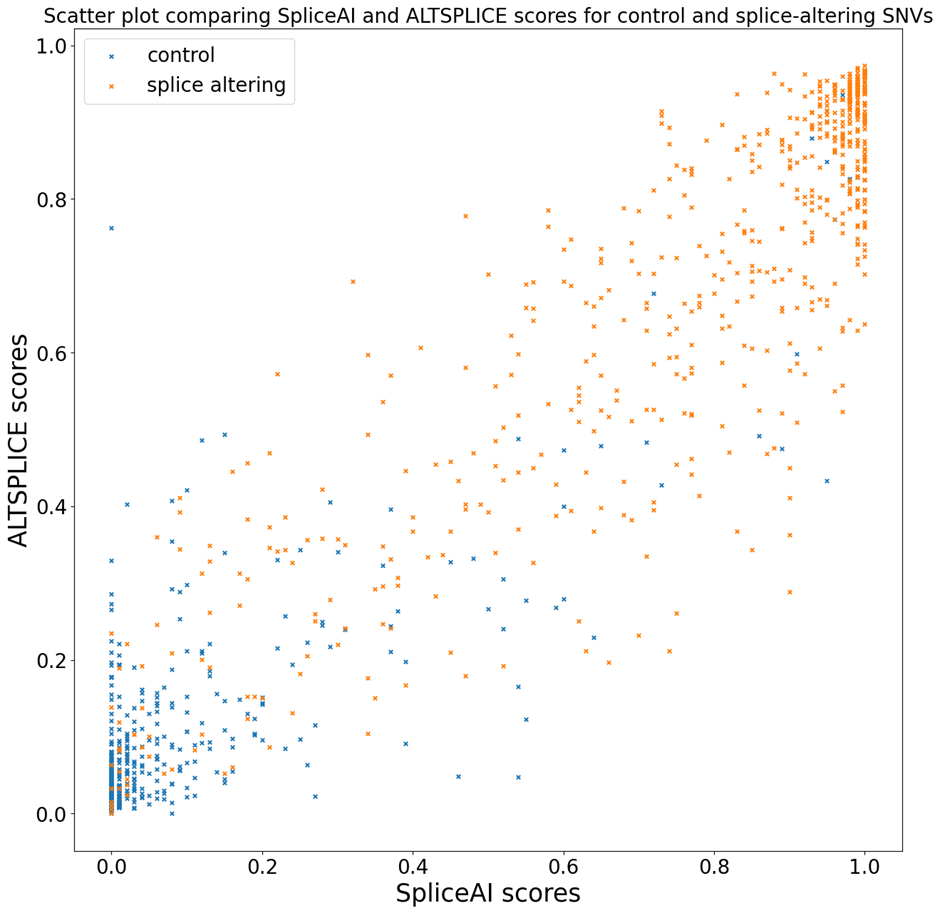


**A**

**B**

**Fig. S17:** Precision recall curve obtained for ALTSPLICE and SpliceAI from a dataset of curated target and control SNVs previously reported in Strauch et al [43]. **B**  Scatter plot comparing ALTSPLICE and *SpliceAI* scores using the same dataset of splice altering and control SNVs.

**
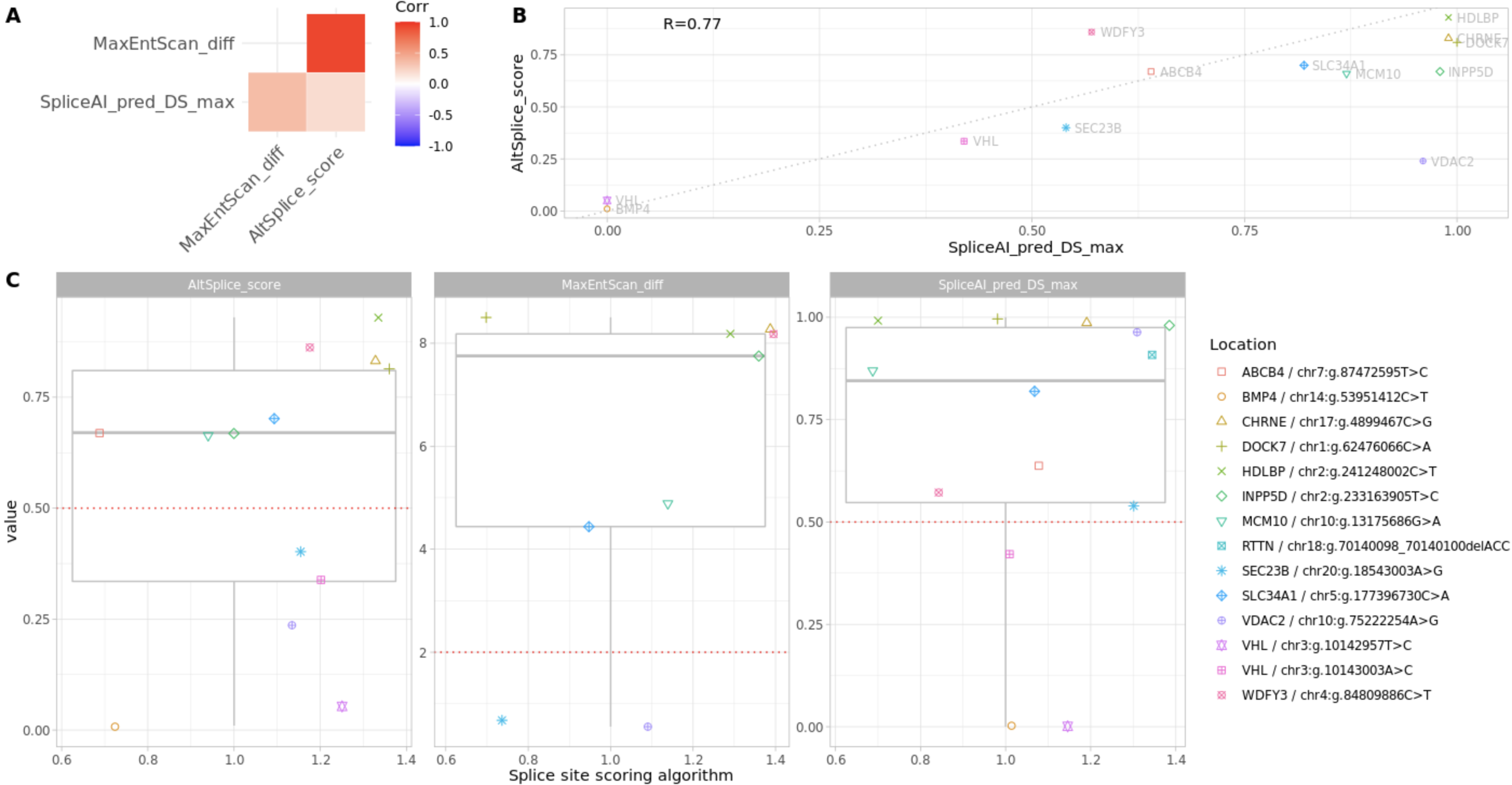
**

**Fig. S18:** Comparison of splice site impact score annotations for 14 candidate splice site/deep intronic variants from main Table 2. **A** correlation matrix plot comparing the scores of the three applied scoring systems: MaxEntScan (entropy difference), SpliceAI (maximum delta score) and ALTSPLICE (model output score, see Methods). **B** Direct comparison of SpliceAI and ALTSPLICE scores show that both algorithms are in good agreement about most considered variants (Pearson’s R=0.77; legend in panel C). **C** Individual scores per algorithm for all candidate variants. Dotted red lines indicate typical cutoffs applied to these scoring systems.


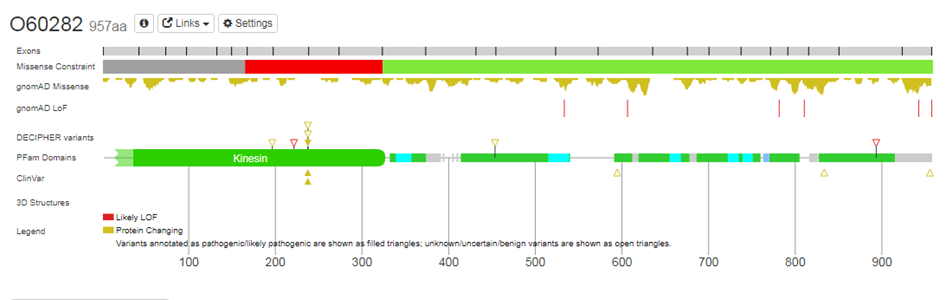


**Fig. S19:** Protein view for the *KIF5C* gene showing that the central segment of the gene (in red) has fewer missense mutations than expected. For residues 165-323, 5 missense changes were observed compared to 46 expected. In contrast, clustering of missense mutations from Decipher and ClinVar can be seen at residue 237. Taken from [www.deciphergenomics.org/gene/KIF5C/overview/protein-genomic-info](https://www.deciphergenomics.org/gene/KIF5C/overview/protein-genomic-info)

.


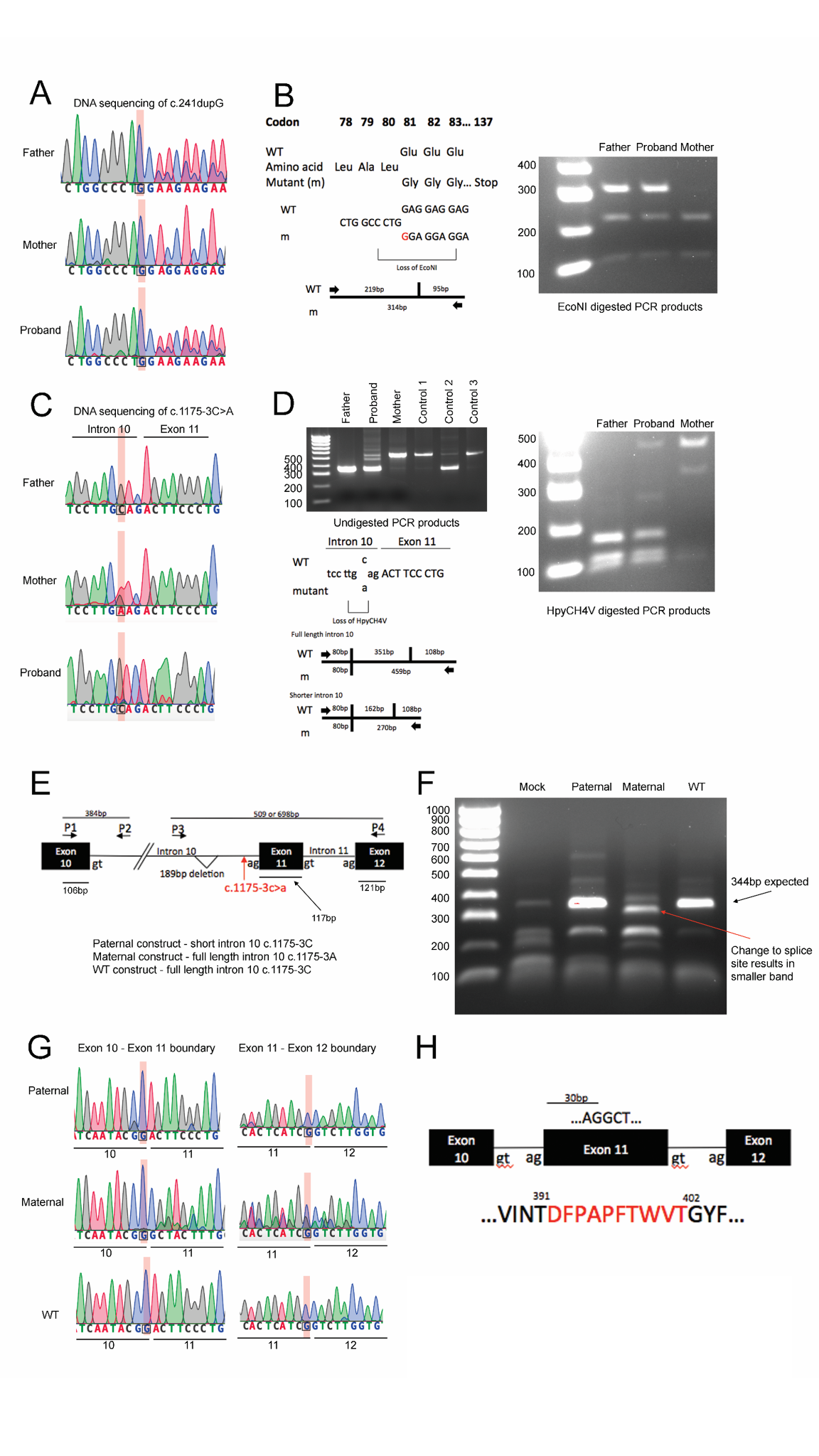


**Fig. S20:** Validation of *SLC34A1* variants in family members and minigene functional analysis of splice variant. **A** Validation of heterozygous *SLC34A1* c.241dupG variant in father and proband by Sanger sequencing. Sequence traces for the father and proband show single peaks upstream of inserted guanine nucleotide at c.241 (guanine 240 is highlighted) and double peaks downstream indicating alleles are out of frame. **B** Confirmation of *SLC34A1* c.241dupG variant in the father and proband by restriction endonuclease digestion. The insertion of a guanine residue at c.241 results in the loss of an EcoNI site. EcoNI digestion of the PCR products from the three family members results in the generation of a 314bp fragment in the father and proband, but not the mother. **C** Validation of heterozygous *SLC34A1* c.1175-3C>A variant (highlighted) in the mother and proband, but not the father, by Sanger sequencing. **D** Agarose gel electrophoresis of the PCR products revealed a shorter fragment of approximately 350bp in the father and proband compared with the mother in which a 539bp band was generated. Interrogation of the sequence trace from the father confirmed a 189bp deletion within *SLC34A1* intron 10, a deletion that is common and was observed in one of the three control individuals (Control 2). The *SLC34A1* c.1175-3C>A variant results in the loss of a HpyCH4V site. Predicted restriction endonuclease cleavage sites within the PCR products containing full length or shorter intronic sequences are shown. HpyCH4V digestion of the PCR products from the three family members results in the generation of a 459bp fragment in the mother and the proband, but not the father, thus confirming the presence of the c.1175-3C>A variant. **E** *SLC34A1* minigene constructs design **F** PCR analysis of cDNA using *SLC34A1* specific primers to identify splice events. The expected fragment size of 344bp that would be generated if the canonical splice donor (gt) and acceptor (ag) sites were used was observed from the paternal and WT constructs. This confirmed that the 189bp intron 10 deletion had no effect on splicing. In contrast, for the maternal construct the band at 344bp was no greater than that observed in mock treated cells confirming that splicing was altered. Instead, the band of greatest intensity was slightly smaller (red arrow). **G** Sanger sequencing of the 344bp bands from the paternal and WT constructs confirmed that the canonical splice donor and acceptor sites within intron 10 and 11 were used. Sanger sequencing of the <344bp band from the maternal construct revealed that the first “AG” within exon 11 was recognised as the splice acceptor and that as a result the first 30 nucleotides of exon 11 were lost from the cDNA. A fragment of 227bp was observed in all transfected cells, which following Sanger sequence analysis was identified as having exon 10 spliced directly onto exon 12, thus excising exon 11. This band was more prominent from the maternal construct compared with the paternal or WT constructs, suggesting this splicing event occurred with greater frequency. **H** The c.1175-3C>A variant results in the first AG site within exon 11 being used as a splice acceptor site resulting in the loss of 30 nucleotides from the beginning of exon 11. This is predicted to result in an in-frame deletion with the loss of 10 amino acids (codons 392-401) located within one of the cytoplasmic topological domains of the sodium-dependent phosphate transport protein 2A.


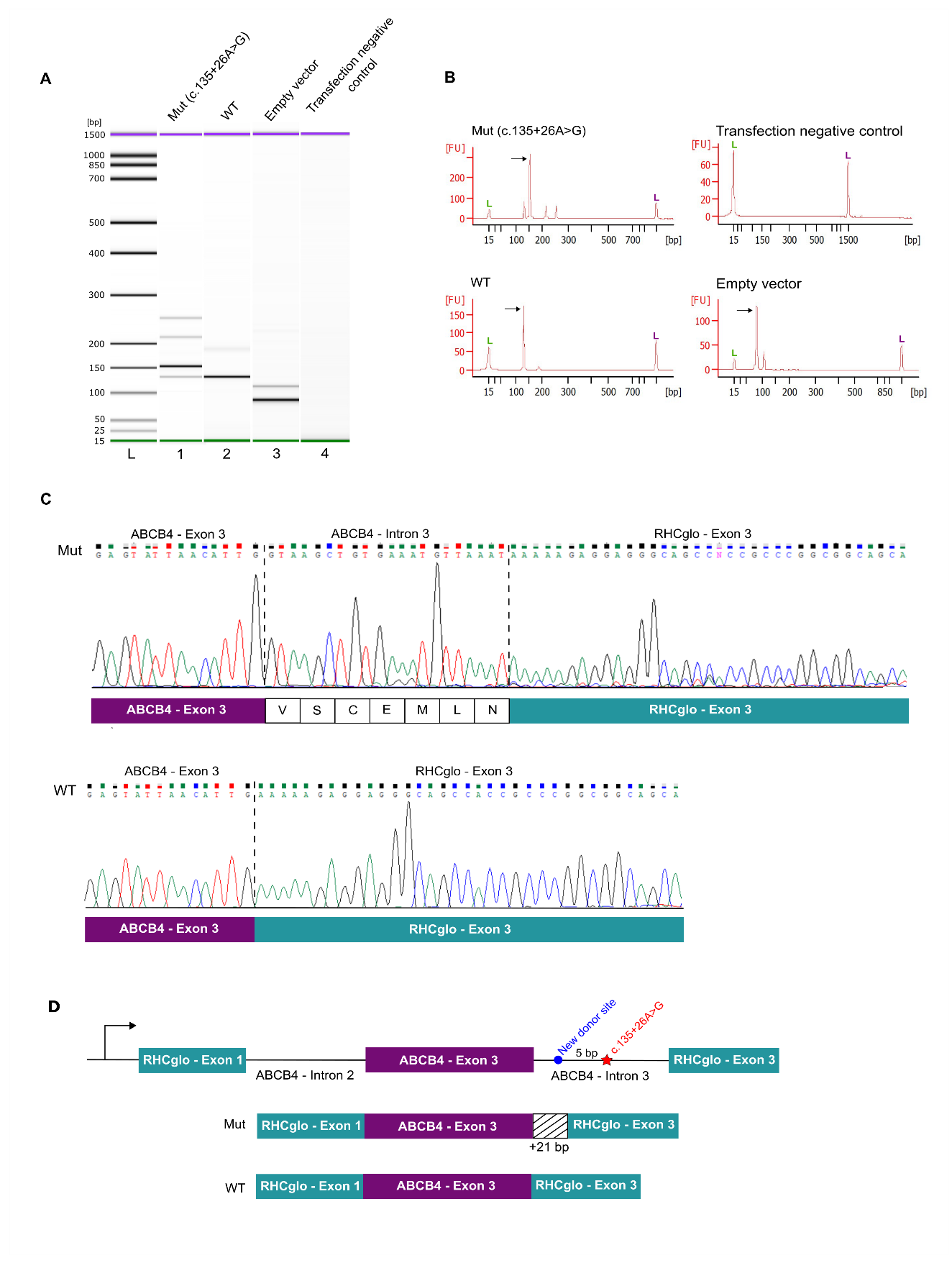
**Fig. S21:** Validation of *ABCB4* variants in family members and minigene functional analysis of the splice variant. A-B The analysis of PCR products from the wildtype (WT) and mutant (Mut) ABCB4 constructs along with empty RHCglo vector and no transfection control on virtual gel (generated with 2100 Agilent Bioanalyzer) and electropherograms demonstrating the extension predicted for the ABCB4 splice variant. C The Sanger sequencing of the mutant ABCB4 construct revealed that the splice variant introduces +21 bp (7 aa) and creates a new donor site 5 bp upstream of the variant. The sequencing result of the WT ABCB4 construct showed normal splicing as expected. D The design of the minigene constructs using RHCglo vector and the representation of the splicing results for the WT and mutant ABCB4 constructs.


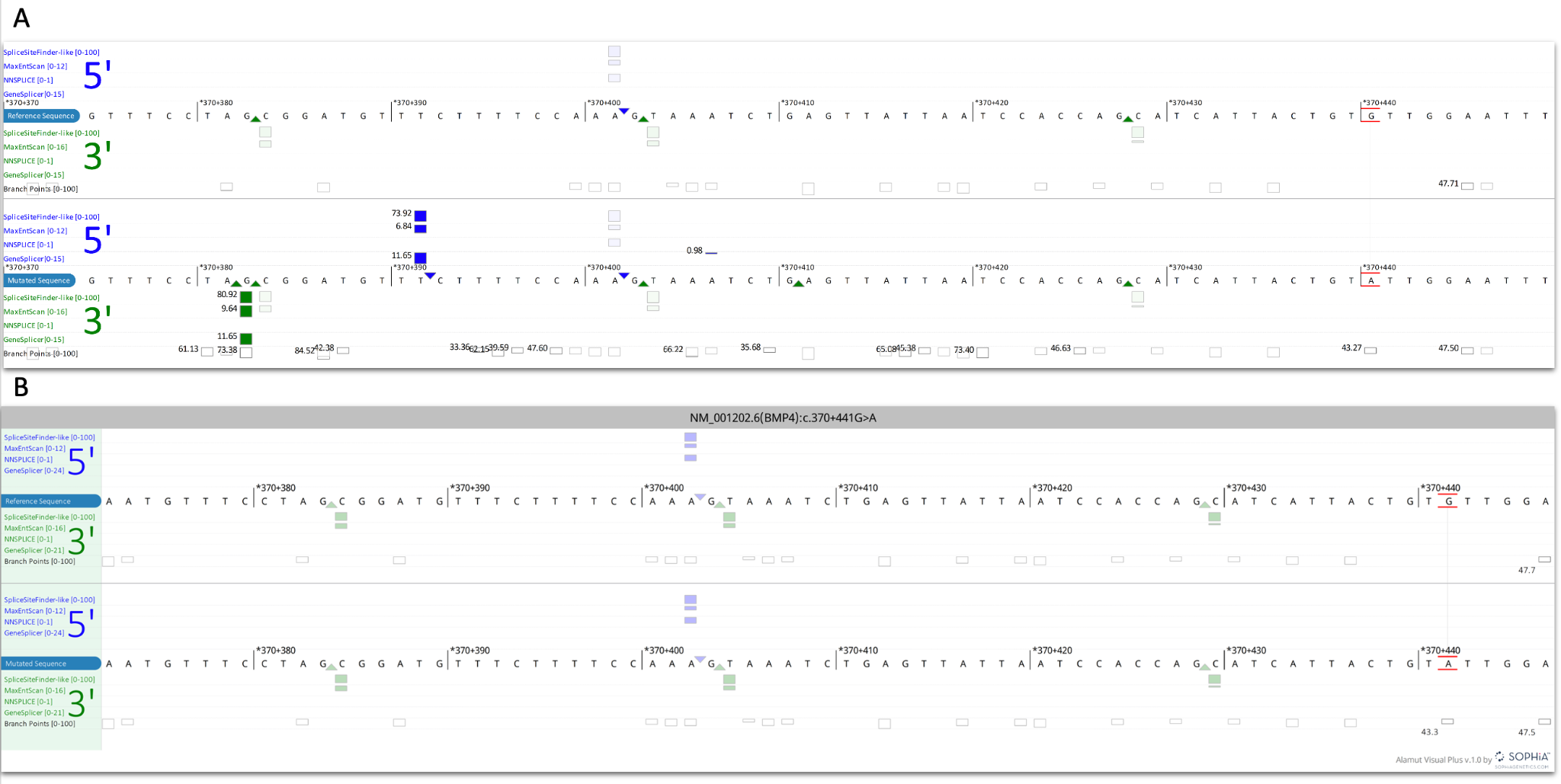

**Fig. S22**: Alamut Genova Software Predictions for the *BMP4* deep intronic variant. **A** Alamut Genova Software Prediction **B** Alamut Visual Plus Software Prediction. The ‘highlight differences’ button was selected. This time, no splice site changes were predicted, in contrast to the previous Alamut version.


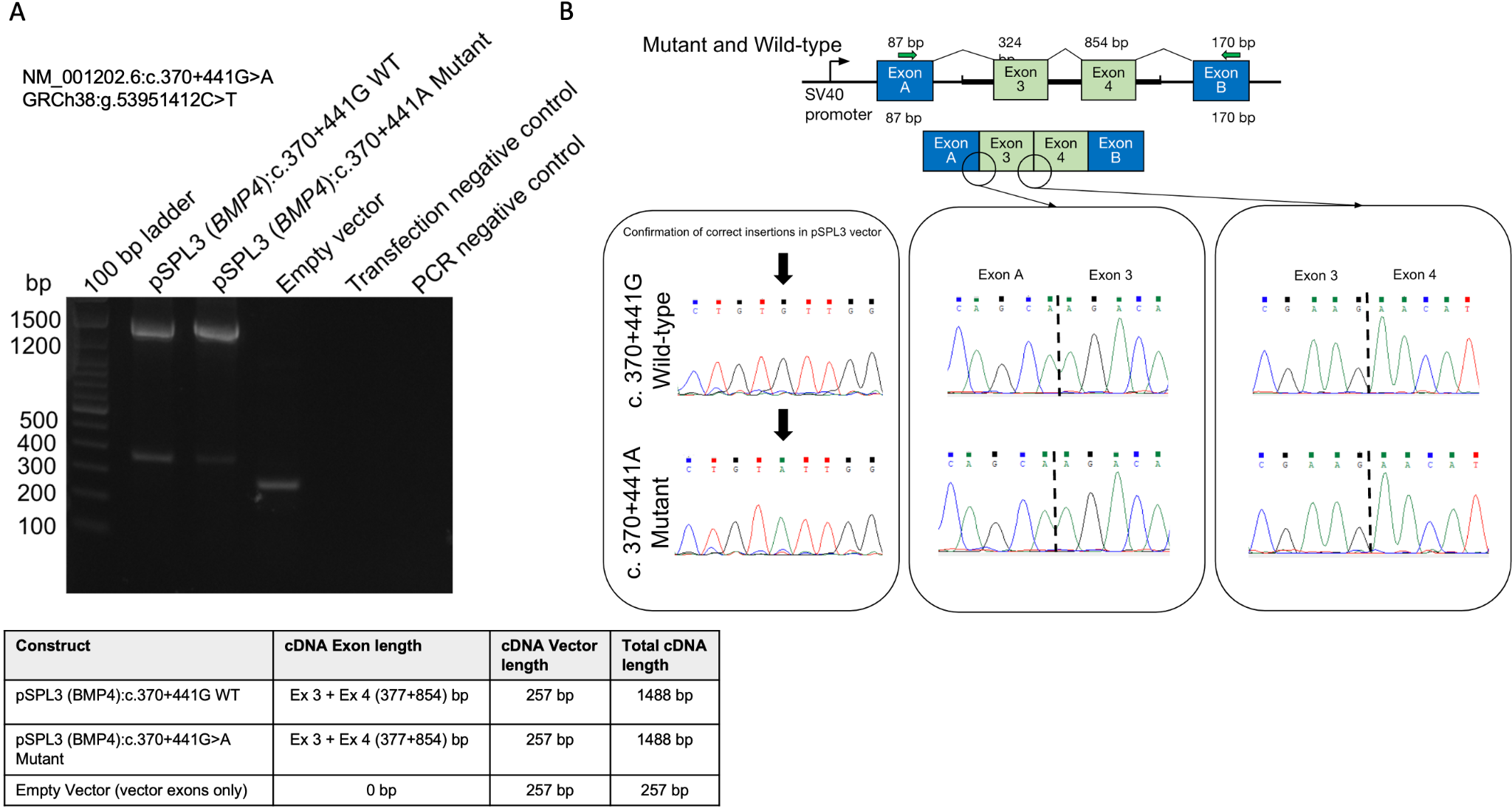


**Fig. S23**: MiniGene assessment of intronic *BMP4* mutation. **A** Gel electrophoresis showing similar sized bands between WT (+441G) and mutant (+441A) construct **B** Sanger sequencing traces confirm the correct insertion into the pSPL3 vector and that the *BMP4* deep intronic splicing variant does not appear to alter splicing in this experimental context.


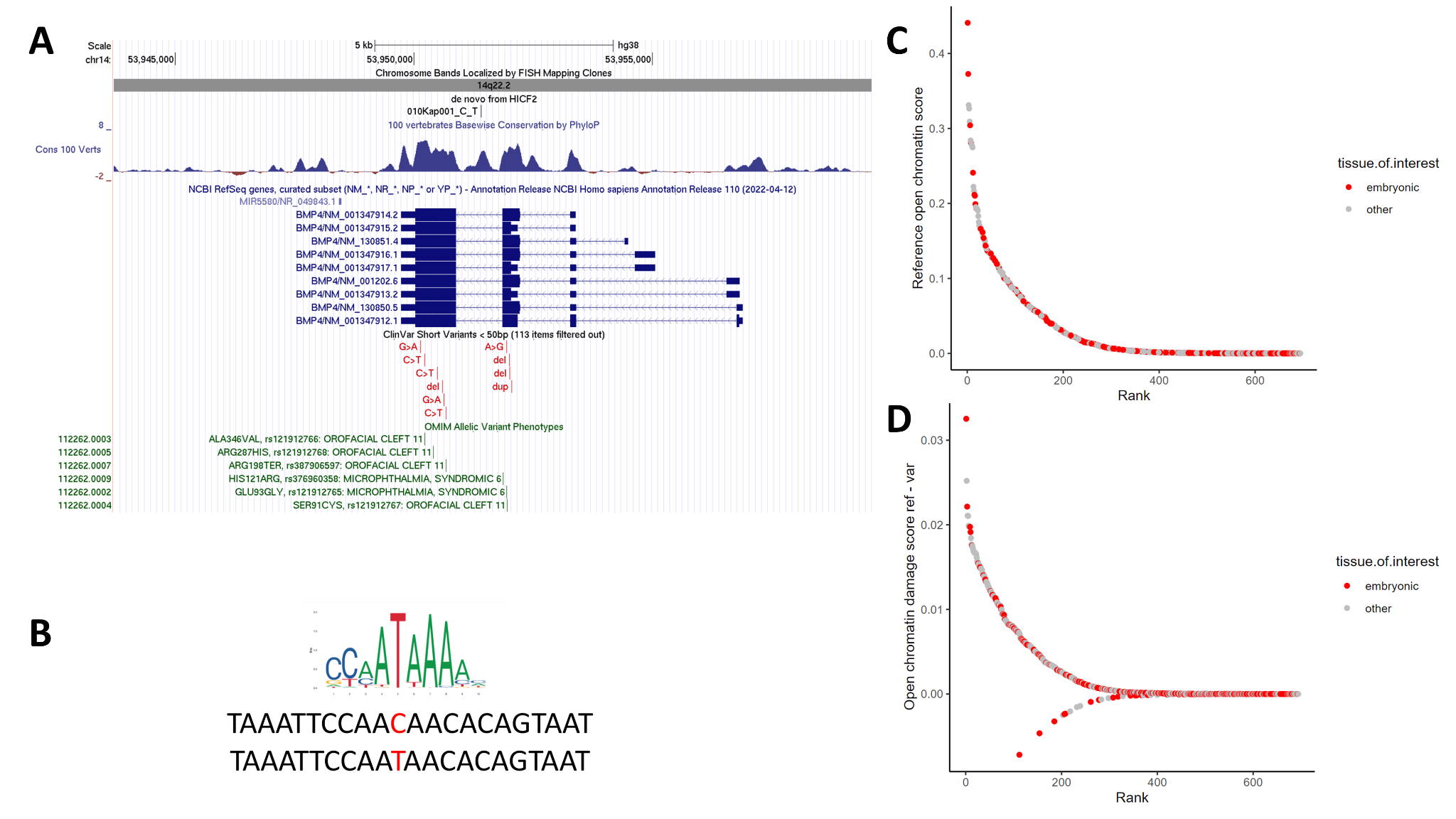


**Fig. S24:** *In silico* investigations of a non-coding *BMP4* variant. **A** UCSC screenshot showing position of deep intronic variant ENST00000245451.9:c.370+441G>A in *BMP4* that arose *de novo* in a patient with Kapur-Toriello syndrome. The variant is in the middle of an intronic region that shows extreme levels of conservation similar to that seen in coding regions. Pathogenic/likely pathogenic variants in ClinVar (red) and in the OMIM Allelic Variant database (green) are exclusively found in coding regions. Interactive version available at <https://genome.ucsc.edu/s/AlistairP/HICF_BMP4>. **B** The variant creates a consensus site for HOXA/B/D13 transcription factor binding. **C,D** DeepHaem *in silico* predictions across 694 ENCODE open chromatin classifiers predicts a weak open chromatin site over the variant (top) and the variant having a weak predicted damaging score of reference - variant of up to 0.03 (bottom). An assessment of the *BMP4* variant effect on chromatin accessibility in the Descartes set of 220 adult tissues did not reveal any changes in open chromatin, supporting this being involved in very early stages of development.


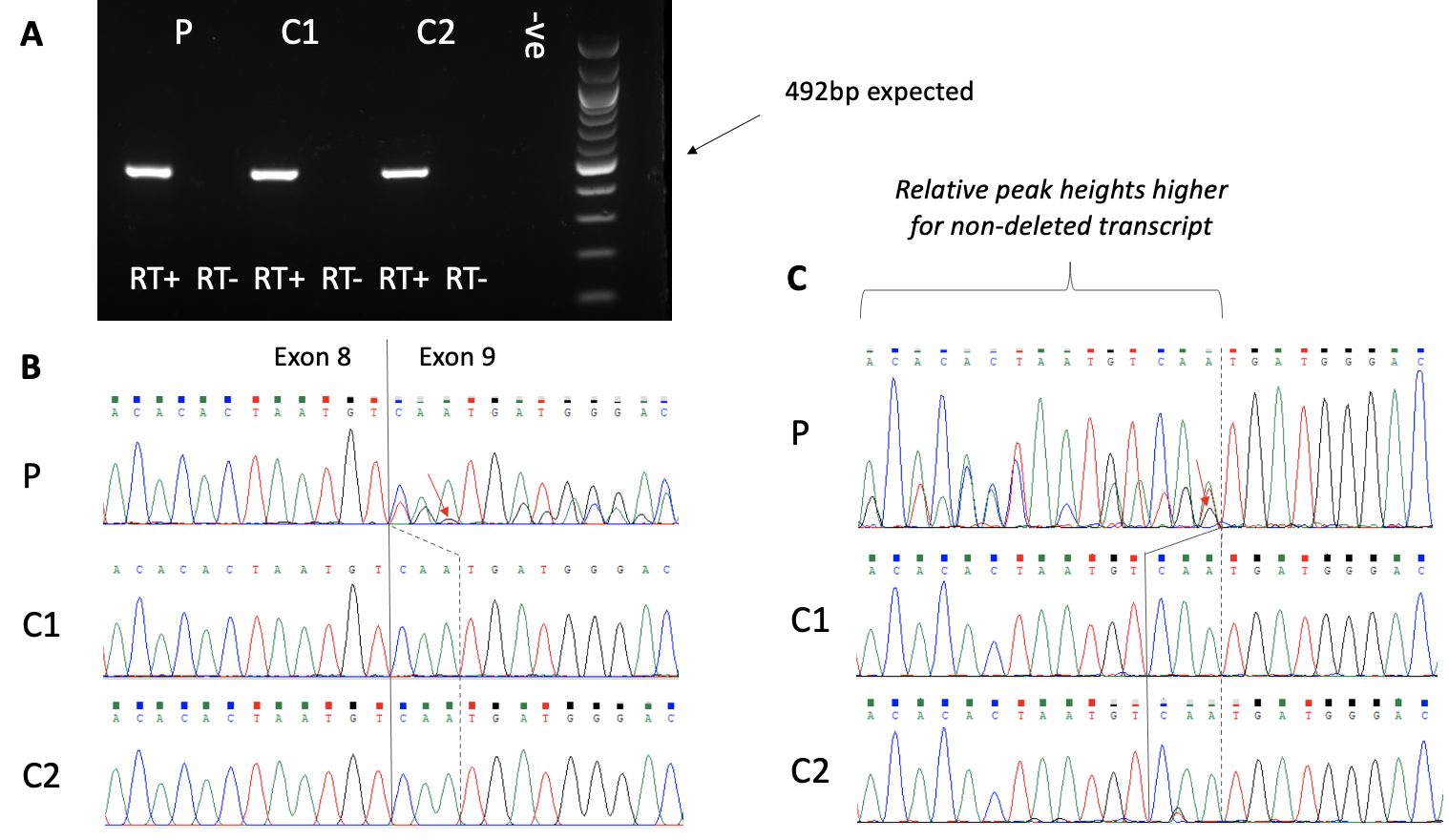


**Fig. S25:** RNA analysis confirms altered splicing for *VDAC2*. **A**) RNA was obtained from fibroblasts from the patient (P) and from two unrelated controls (C1 and C2). RT-PCR was performed with primers VDAC2_7F (ACCTTCTCACCAAACACAGGA) and VDAC2_11R (CCCATCTACCAGAGCAGAGAG). No product was seen in the absence of reverse transcriptase enzyme (RT-), confirming the product is not due to genomic DNA contamination. Although a single band was consistent with the expected 492bp amplicon, agarose gels are not able to resolve small size differences. **B**) Sanger sequencing of the RT-PCR products from primer 7F indicates a 3bp deletion at the start of exon 9. **C**) Sanger sequencing of the RT-PCR products from primer 11R confirms this 3bp deletion. Relative peak heights are consistently higher for the non-deleted transcript. This suggests that there is still some use of the canonical acceptor site, even with the G allele present (otherwise the ratio should be 50:50). The red arrows show some residual signal for the G allele at c.587A>G (p.Asn196Ser), which is further evidence that the splicing alteration is “leaky”. Exon numbering is based on ENST00000332211.10: and c.585_587delCAA predicts a single amino acid deletion p.(Asn196del)


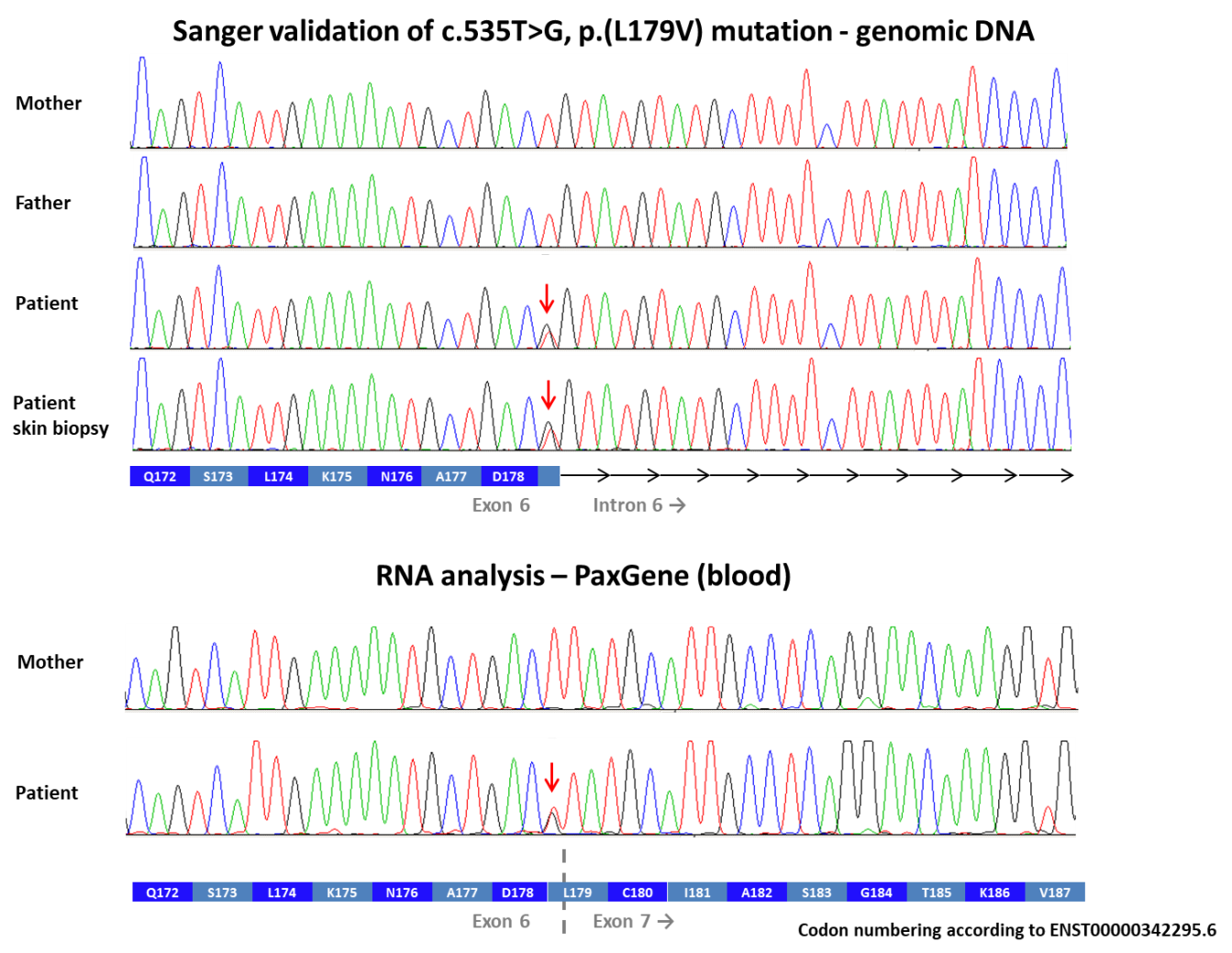


**Fig. S26:** Sanger validation of a *de novo* germline *RBPJ* variant c.535T>G (p.L179V) in a patient with Klippel Trenaunay syndrome. Although the variant involves the last base in exon 6, RNA analysis confirmed no effect on splicing. However the finding of a pathogenic *PIK3CA* somatic mosaic variant in this patient suggested that this variant in *RBPJ*, a VUS when classified by ACMG guidelines, was not pathogenic for the condition.

**
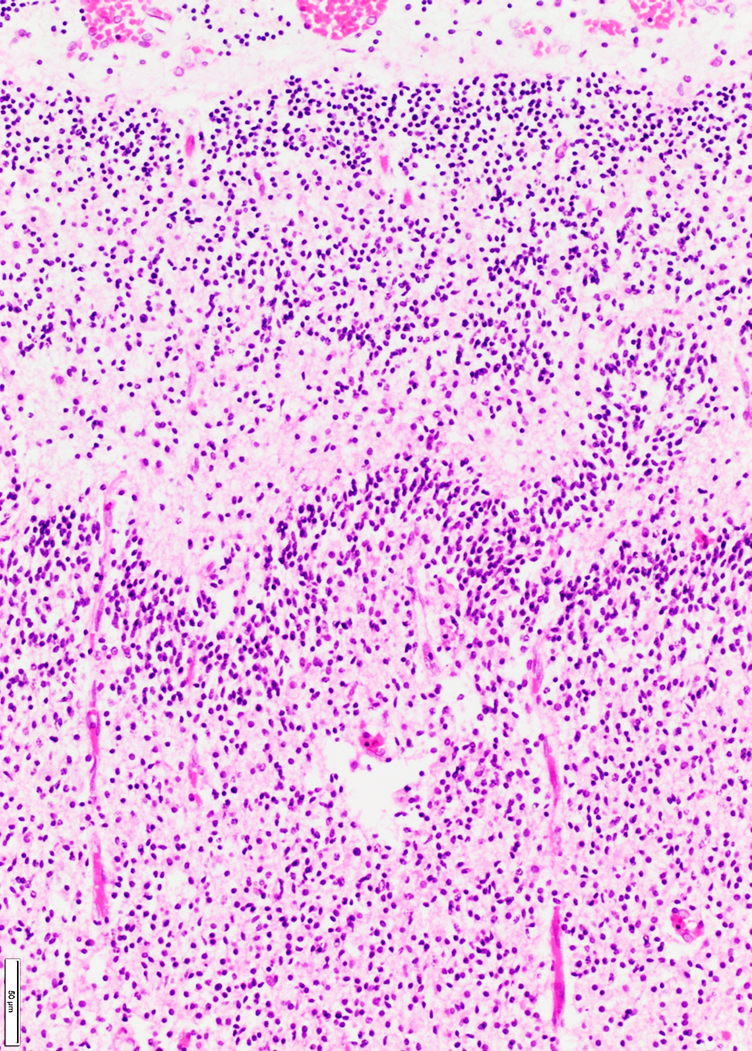
**

Band of immature neurons

**Fig. S27:** Histology of cerebral cortex from patient with polymicrogyria and arthrogryposis

The image shows polymicrogyria of the cerebral cortex in patient 010PMA001 who was found to have a frameshift variant (p.Phe36fs) in *RMND1* (gestational age 27/40 weeks, Haematoxylin and Eosin, scale bar 50 micrometres). Pia mater is at the top of the image. Note excessively folded and festooned band of immature neurons in the centre of the image (shown by arrow). There are also focal breaches of the pia with over-migration of small neurons into the leptomeninges.


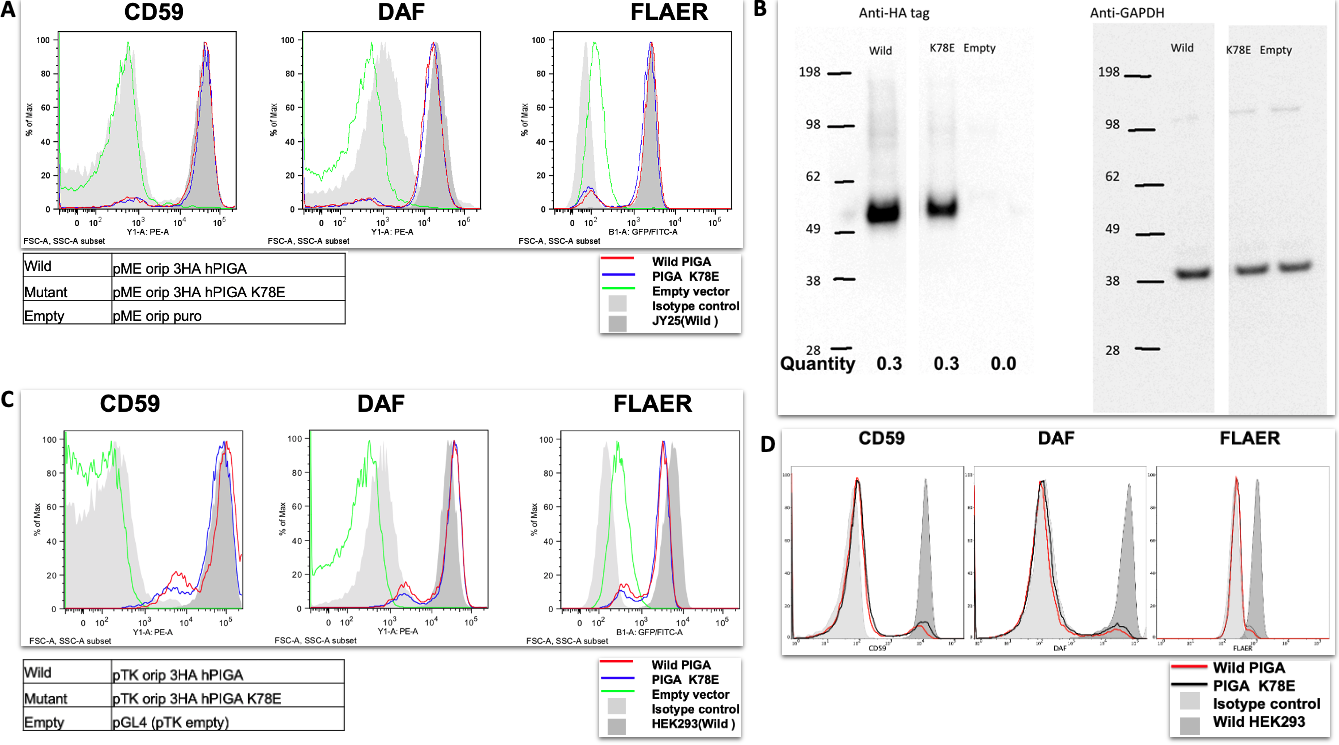


**Fig. S28:** PIGA functional analysis. **A** Mutant PIGA could rescue the surface expression of GPI-AP at the similar level as the wild PIGA (Vector backbone: pME). **B** Protein expression of wild type and mutant PIGA in the lysates of the cells in panel A were analysed by western blotting. Band intensity was normalised by GAPDH expression as a loading control and by luciferase activity as a transfection efficiency. Expression of mutant PIGA protein was similar to wild PIGA protein. **C** PIGA KO HEK293 cells were transfected with wild or mutant PIGA expressing plasmids (Vector backbone: pTK) driven by the weaker promoter and analysed the surface expression of GPI anchored proteins by FACS. Mutant PIGA could rescue the surface expression of GPI-AP at the similar level as the wild PIGA. **D** Mutant PIGA could rescue the surface expression of GPI-AP at the similar level as the wild PIGA (Vector backbone: pTA).

## Supplementary References

References

1. Giacopuzzi, E. *Bioinformatics pipeline for analysis of whole genome sequencing data* 2022; Available from: <https://github.com/edg1983/WGS_pipeline>.

2. Li, H. and R. Durbin, *Fast and accurate short read alignment with Burrows-Wheeler transform.* Bioinformatics, 2009. **25**(14): p. 1754-60.

3. Faust, G.G. and I.M. Hall, *SAMBLASTER: fast duplicate marking and structural variant read extraction.* Bioinformatics, 2014. **30**(17): p. 2503-5.

4. Pockrandt, C., et al., *GenMap: ultra-fast computation of genome mappability.* Bioinformatics, 2020. **36**(12): p. 3687-3692.

5. Pedersen, B.S. and A.R. Quinlan, *Mosdepth: quick coverage calculation for genomes and exomes.* Bioinformatics, 2018. **34**(5): p. 867-868.

6. Pedersen, B.S., et al., *Somalier: rapid relatedness estimation for cancer and germline studies using efficient genome sketches.* Genome Med, 2020. **12**(1): p. 62.

7. Poplin, R., et al., *A universal SNP and small-indel variant caller using deep neural networks.* Nat Biotechnol, 2018. **36**(10): p. 983-987.

8. Yun, T., et al., *Accurate, scalable cohort variant calls using DeepVariant and GLnexus.* Bioinformatics, 2021.

9. Popitsch, N. *Python pipelines for the comprehensive annotation of cohort-wide VCF files.* 2022; Available from: <https://github.com/popitsch/cohort_varan>.

10. Cingolani, P., et al., *A program for annotating and predicting the effects of single nucleotide polymorphisms, SnpEff: SNPs in the genome of Drosophila melanogaster strain w1118; iso-2; iso-3.* Fly (Austin), 2012. **6**(2): p. 80-92.

11. Pedersen, B.S., R.M. Layer, and A.R. Quinlan, *Vcfanno: fast, flexible annotation of genetic variants.* Genome Biol, 2016. **17**(1): p. 118.

12. Larson, D.E., et al., *svtools: population-scale analysis of structural variation.* Bioinformatics, 2019. **35**(22): p. 4782-4787.

13. Zhang, L., et al., *Comprehensively benchmarking applications for detecting copy number variation.* PLoS Comput Biol, 2019. **15**(5): p. e1007069.

14. Abel, H.J., et al., *Mapping and characterization of structural variation in 17,795 human genomes.* Nature, 2020. **583**(7814): p. 83-89.

15. Yu, J., et al., *SVRare: discovering disease-causing structural variants in the 100K Genomes Project.* medRxiv, 2022.

16. Swaminathan, G.J., et al., *DECIPHER: web-based, community resource for clinical interpretation of rare variants in developmental disorders.* Hum Mol Genet, 2012. **21**(R1): p. R37-44.

17. Yu, J. *App to visualise structural variants results from the SVRare bioinformatics algorithm*. 2022; Available from: <https://github.com/Oxford-Eye/SVRare-js>.

18. Dolzhenko, E., et al., *ExpansionHunter: a sequence-graph-based tool to analyze variation in short tandem repeat regions.* Bioinformatics, 2019. **35**(22): p. 4754-4756.

19. Deelen, P., et al., *Improving the diagnostic yield of exome- sequencing by predicting gene-phenotype associations using large-scale gene expression analysis.* Nat Commun, 2019. **10**(1): p. 2837.

20. Robinson, P.N., et al., *Improved exome prioritization of disease genes through cross-species phenotype comparison.* Genome Res, 2014. **24**(2): p. 340-8.

21. Giacopuzzi, E. *Nextflow pipeline for HPO-based prioritization (GADO and Exomiser)*. 2022; Available from: <https://github.com/edg1983/NF_HPO_prioritize>.

22. Itan, Y., et al., *The human gene damage index as a gene-level approach to prioritizing exome variants.* Proc Natl Acad Sci U S A, 2015. **112**(44): p. 13615-20.

23. Petrovski, S., et al., *The Intolerance of Regulatory Sequence to Genetic Variation Predicts Gene Dosage Sensitivity.* PLoS Genet, 2015. **11**(9): p. e1005492.

24. Giacopuzzi, E., N. Popitsch, and J.C. Taylor, *GREEN-DB: a framework for the annotation and prioritization of non-coding regulatory variants from whole-genome sequencing data.* Nucleic Acids Res, 2022. **50**(5): p. 2522-2535.

25. Smedley, D., et al., *A Whole-Genome Analysis Framework for Effective Identification of Pathogenic Regulatory Variants in Mendelian Disease.* Am J Hum Genet, 2016. **99**(3): p. 595-606.

26. Shihab, H.A., et al., *An integrative approach to predicting the functional effects of non-coding and coding sequence variation.* Bioinformatics, 2015. **31**(10): p. 1536-43.

27. Wells, A., et al., *Ranking of non-coding pathogenic variants and putative essential regions of the human genome.* Nat Commun, 2019. **10**(1): p. 5241.

28. Giacopuzzi, E. *Annotation of non-coding regulatory variants using GREEN-DB, prediction scores, conservation and population allele frequency*. 2023; Available from: <https://github.com/edg1983/GREEN-VARAN>.

29. Downes, D.J., et al., *Identification of LZTFL1 as a candidate effector gene at a COVID-19 risk locus.* Nat Genet, 2021. **53**(11): p. 1606-1615.

30. Schwessinger, R., et al., *DeepC: predicting 3D genome folding using megabase-scale transfer learning.* Nat Methods, 2020. **17**(11): p. 1118-1124.

31. Luo, Y., et al., *New developments on the Encyclopedia of DNA Elements (ENCODE) data portal.* Nucleic Acids Res, 2020. **48**(D1): p. D882-D889.

32. Schwessinger, R. *Implementation of a deep convolutional neural network for predicting chromatin features from DNA sequence*. 2022; Available from: <https://github.com/Hughes-Genome-Group/deepHaem>.

33. Giacopuzzi, E. *Shiny app to explore and filter annotated variants results* 2021; Available from: <https://github.com/edg1983/Variant_explorer>.

34. Martin, A.R., et al., *PanelApp crowdsources expert knowledge to establish consensus diagnostic gene panels.* Nat Genet, 2019. **51**(11): p. 1560-1565.

35. Yeo, G. and C.B. Burge, *Maximum entropy modeling of short sequence motifs with applications to RNA splicing signals.* J Comput Biol, 2004. **11**(2-3): p. 377-94.

36. Jaganathan, K., et al., *Predicting Splicing from Primary Sequence with Deep Learning.* Cell, 2019. **176**(3): p. 535-548 e24.

37. Bionano_Genomics, *Bionano Solve Theory of Operation: Structural Variant Calling. Document Number: 30110 (Revision K).* 2018.

38. Ormondroyd, E., et al., *Insights from early experience of a Rare Disease Genomic Medicine Multidisciplinary Team: a qualitative study.* Eur J Hum Genet, 2017. **25**(6): p. 680-686.

39. Ewels, P., et al., *MultiQC: summarize analysis results for multiple tools and samples in a single report.* Bioinformatics, 2016. **32**(19): p. 3047-8.

40. Taylor, J.C., et al., *Factors influencing success of clinical genome sequencing across a broad spectrum of disorders.* Nat Genet, 2015. **47**(7): p. 717-726.

41. Deng, Y., et al., *HPOSim: an R package for phenotypic similarity measure and enrichment analysis based on the human phenotype ontology.* PLoS One, 2015. **10**(2): p. e0115692.

42. Frazer, J., et al., *Disease variant prediction with deep generative models of evolutionary data.* Nature, 2021. **599**(7883): p. 91-95.

43. Strauch, Y., et al., *CI-SpliceAI-Improving machine learning predictions of disease causing splicing variants using curated alternative splice sites.* PLoS One, 2022. **17**(6): p. e0269159.
