# Supplementary material for: Structural and non-coding variants increase the diagnostic yield of clinical whole genome sequencing for rare diseases
Source: Genome Med. 2023 Nov 9;15:94. doi: 10.1186/s13073-023-01240-0 (PMC10636885; doi:10.1186/s13073-023-01240-0)
Supplement: Supplementary file 2 — Additional file 2. Supplementary clinical case information for selected cases. [file 13073_2023_1240_MOESM2_ESM.docx]

Supplementary CLINICAL CASE Information

Structural and non‑coding variants increase the diagnostic yield of clinical whole genome sequencing for rare diseases

Table of Contents

[005KLI001 - example of somatic mutation (*PIK3CA*) 2](#_Toc132715868)

[008CON004 – patient with congenital T-cell lymphopaenia (*VDAC2 and INPP5D*) 2](#_Toc132715869)

[010AIC002 - example of structural variant leading to changed diagnosis (*ARX*) 2](#_Toc132715870)

[010KAP001 – example of deep intronic variant (*BMP4*) 3](#_Toc132715871)

[010NEU001 – example of complex structural variant 4](#_Toc132715872)

[010PI4K001 - example of blended phenotype (*WDFY3* and *KIF5C*) 4](#_Toc132715873)

[010PMA001 – example of extending clinical range (*RMND1*) 5](#_Toc132715874)

[020CHA001 - example of secondary finding (*FBN1*) 6](#_Toc132715875)

[021BIL002 – patient with bilateral hippocampal sclerosis (*ALG13*) 7](#_Toc132715876)

[021MTL001 – patient with mesial temporal lobe epilepsy (*APP*) 7](#_Toc132715877)

[037CON002 – patient with congenital myasthenic syndromes (*SLC5A7*) 7](#_Toc132715878)

[039FIB001 – example of a genetic diagnosis that enabled a life-saving intervention (*NPHP3*) 8](#_Toc132715879)

[058FANC001 – patient with nephrocalcinosis example of compound heterozygous mutations involving a splice site (*SLC34A1*) 9](#_Toc132715880)

[066GCD001 - Genetic Cholestatic Disease (*ABCB4*) 9](#_Toc132715881)

[066NNH001 – Neonatal haemochromatosis (*HSD3B7* and *BMP6*) 10](#_Toc132715882)

[Supplementary References 11](#_Toc132715883)

## 005KLI001 - example of somatic mutation (*PIK3CA*)

This female patient’s diagnosis was of Klippel-Trenaunay-Weber-like syndrome of the right hindquarter with splenic haemangiomas, hepatic haemangiomas and telangiectatic lesions of the right upper thigh and buttock. Germline WGS did not identify any likely pathogenic variants and somatic mutation testing was therefore undertaken. DNA was extracted from skin samples from affected haemangioma lesions and high coverage targeted next generation sequencing using the Ion Torrent platform confirmed a mosaic somatic variant in *PIK3CA* (8%). On referral of the patient to St George’s Hospital, repeat sequencing of a skin biopsy from the affected leg using the Swift Biosciences Accel-Amplicon® Plus 57G Pan-Cancer Profiling Panel and Illumina MiSeq confirmed mosaic variants in *PIK3CA* (4%) and excluded variants in *BRAF, NRAS* and *KRAS.* The patient has had two courses of sclerotherapy which provided some benefit and had been on interferon which has now been discontinued. She is currently being recruited for the EPIK randomised controlled study of alpelisib, a PI3K inhibitor. Follow up ultrasound scans of her spleen and liver are planned to check that the haemangiomas are not increasing in size or number. We note that germline WGS analysis revealed a *de novo* missense variant, p.(L179V) in *RBPJ,* a known gene associated with Adams Oliver syndrome (OMIM #614814). Although the variant involved the last base in exon 6, *in silico* tools suggested no effect on splicing which was confirmed by RNA analysis (Additional file 1: Fig. S26). The variant in *RBPJ* was therefore not considered pathogenic for this patient.

## 008CON004 – patient with congenital T-cell lymphopaenia (*VDAC2 and INPP5D*)

The proband, born to non-consanguineous parents, presented with severe congenital isolated T-cell lymphopaenia during his first year of life. As he presented with life threatening infections, including severe varicella and mycobacterial infections, he was treated with haematopoietic stem cell transplantation. However, despite 100% donor engraftment there was no T-cell recovery possibly suggesting a thymic stromal cell defect. Other clinical features included malformation of the lower limbs. Initial genetic studies including FISH and primary immune deficiency gene panel did not reveal a molecular cause for his immune deficiency. Therefore WGS was undertaken in the context of this study. Two *de novo* splice site variants of uncertain significance in *VDAC2* and *INPP5D* were identified (Table 2), with that in *VDAC2* being followed up by RNA studies (Additional file 1: Fig. S25). The c.1437+2T>C variant in intron 11 of *INPP5D* is predicted to weaken the canonical splice donor site efficiency and result in usage of an alternative acceptor site leading to a premature stop codon. INPP5D (also called SHIP1*)* is a protein phosphatase which regulates the PI3K signalling pathway and plays a key role in both T cell biology [1] and mammalian skeletal development [2], and is therefore a putative candidate for one or both elements of the patient’s phenotype. Two isoforms of *INPP5D* have been described, a long one and a shorter isoform, s-SHIP, which is specific to embryonic stem cells and has been shown to arise from use of an alternative promoter in intron 5 in mice [3]. Since the patient had been treated with haematopoietic stem cell transplantation for genetically undefined T-B+NK+ SCID during infancy, we could not confirm the splice site variant in blood, and the gene is not expressed in primary skin fibroblasts. Expression in additional cell types is now being investigated.

## 010AIC002 - example of structural variant leading to changed diagnosis (*ARX*)

This patient experienced onset of spasms at 20 days and exhibited mild psychomotor delay, hypoplastic corpus callosum and bilateral hippocampal malrotation which was more marked on the left side. Ophthalmological consulting, performed at approximately 1 year of age, revealed a normal eye fundus and no chorioretinal lacunae were reported. Array CGH testing was negative. The patient was originally diagnosed with Aicardi syndrome, a rare congenital disorder characterized by a classic triad of chorioretinal lacunae, infantile spasms, and agenesis of the corpus callosum. Although no chorioretinal lacunae, the expansion of diagnostic criteria to include 2 major features from the triad plus at least two other major or supporting features ([www.ncbi.nlm.nih.gov/books/NBK1381](https://www.ncbi.nlm.nih.gov/books/NBK1381/)) led to this diagnosis.

**Major features:** malformations (mostly polymicrogyria); Periventricular and subcortical heterotopia; Cysts around third cerebral ventricle and/or choroid plexus; Optic disc/nerve coloboma or hypoplasia

**Supporting features:** Vertebral and rib abnormalities; Microphthalmia; "Split-brain" EEG; Gross cerebral hemispheric asymmetry; Vascular malformations or vascular malignancy

The identification of a structural variant in *ARX* (Table 1), a known gene for developmental and epileptic encephalopathy Type 1 (DEE1) led to a change of clinical diagnosis to DEE1 for this patient and was missed by the initial array CGH testing due to the size of the deletion (only one probe in region) and the high GC rich content of the region.

## 010KAP001 – example of deep intronic variant (*BMP4*)

A 14 year old boy was referred to the cleft genetics clinic due to multiple congenital anomalies including severe bilateral cleft lip and palate, midline nasal dysplasia, microphthalmos and cardiac abnormalities including an atrio-ventricular septal defect, hyperplastic right ventricle and truncus arteriosus. He is the only child of a non-consanguineous Caucasian couple. There was no significant family history of note. He was born following a normal pregnancy and delivery, with a low birth weight. The bilateral cleft lip and palate and right sided microphthalmos were identified soon after birth. He has had multiple surgical procedures for repair of the cleft and has a prosthesis for his right eye. His cardiac defects were identified when he was 6 weeks old and he has had multiple procedures, including Fontan procedure. He also had a gastrostomy inserted due to persistent feeding difficulties.

Musculoskeletal abnormalities have included difficulty with opposing the left thumb and scoliosis with hemivertebrae. He had repositioning at 5 years of age on his hand and surgery for scoliosis at 12 years of age. He attended a special needs school until 5 years of age but subsequently joined a mainstream school where he made slow but steady progress. His height has been below the 0.4^th^ centile and he was treated with growth hormone. An MRI brain scan identified a small shallow pituitary fossa and hypoplasia of the corpus callosum. On examination, he was noted to have facial asymmetry, prosthetic right eye, repaired bilateral cleft lip with columella extending below the nares, low posterior hairline, short neck and abnormal thumbs bilaterally. A clinical diagnosis of Kapur-Toriello Syndrome (KTS) was made based on the combination of the multiple congenital anomalies and the distinctive nasal dysmorphology [4]. Although a genetic aetiology is suspected for KTS, no causal gene has been found so far.

Whole genome sequencing through the OxClinWGS study identified a *de novo* intronic variant in the *BMP4* gene. *BMP4* variants are known to cause Syndromic microphthalmia 6 (OMIM #607932) and Nonsyndromic cleft lip and palate 11 (OMIM #600625). They can also cause congenital healed cleft lip with a collapsed nostril. Syndromic microphthalmia 6 patients have also been reported to have additional congenital anomalies such as cleft lip and palate, digital anomalies, spine abnormalities, pituitary abnormalities and kidney and genital anomalies. It therefore appears likely that Kapur-Toriello syndrome is part of the spectrum of anomalies seen with *BMP4* variants. Experiments to confirm the pathogenicity of this intronic variant are underway. Although an original version of Alamut software indicated this was a splice site variant, later versions did not support that (Additional file 1: Fig. S22), nor did Splice AI or ALTSPLICE, and this was confirmed by a minigene experiment (Additional file 1: Fig. S23). DeepHaem predicts that the variant may create a HOX TF binding site in a weakly accessible chromatin region (Additional file 1: Fig. S24), a hypothesis which is being explored further experimentally.

## 010NEU001 – example of complex structural variant

Two brothers, born to a non-consanguineous couple, were referred to the Clinical Genetics department due to global development delay. The first child was referred at the age of 14 months due to severe hypotonia and developmental delay. He was born following a pregnancy complicated by gestational diabetes, for which the mother took metformin during the pregnancy. He had an average birth weight of 6lbs and 11oz and developed hypoglycemia, which was treated with glucose and nasogastric feeds. He developed jerky movements from 3 days of age. His developmental milestones were severely delayed, and he was unable to hold up his neck at the age of 14 months; he had no words in his speech with limited understanding of language. At the age of 3 years, he was still not walking with a personalised wheelchair, was non-verbal and had increased tone in limbs with jerky, abnormal movements. The younger brother was seen at the genetics clinic at the age of 8 months with similar but slightly milder features. He was floppy with dystonic movements. There was no significant improvement in his motor milestones and he remained non-verbal. The parents were both asymptomatic but there is a family history of a neuromuscular disorder in mother’s maternal male cousins and in mother’s maternal uncles.

A microarray CGH identified a 590kb duplication of Xp22.1 (Table 1). Further molecular cytogenetic investigations identified a derivative X chromosome where one copy of Xp22.1 was inserted into the long arm of the X chromosome with a possible breakpoint at Xq27. Both brothers had the same X chromosome rearrangement, which had been inherited from their mother. Further cascade testing in the family showed that the maternal grandmother, her sister and the maternal great grandmother carried the X chromosome anomaly, explaining the cause of the neuromuscular disorder in their affected sons.

## 010PI4K001 - example of blended phenotype (*WDFY3* and *KIF5C*)

A non-consanguineous couple terminated a pregnancy for congenital abnormalities thought to be similar to those described in our 2015 study “Germline recessive mutations in *PI4KA* are associated with perisylvian polymicrogyria, cerebellar hypoplasia and arthrogryposis” [5]. *In utero* scan findings had included a small cerebellum, small chin, clenched hands, fixed and extended legs, talipes, left sided liver, abdominal cyst and polyhydramnios. On post-mortem examination, additional findings comprised cleft palate and micrognathia, fixed flexion of the fingers bilaterally and marked internal rotation of the right hip. The hips were flexed and knees fixed in extension, with severe bilateral talipes. There was a prominent sacrum and scoliosis. Facial features were similar to the published family and included a large nose. Post mortem X-rays showed bilateral hip dislocation, dorsi-flexed feet, extended knees, angulated sacrum, scoliosis and abnormal finger, wrist and elbow positions. There was no evidence of an underlying bone dysplasia. Microarray results were normal and a cord sample was sent for DNA storage.

The family was recruited to the ‘Structural Brain Abnormalities and Learning Disabilities’ study in February 2016. Although the submitter requested *PI4KA* testing based on the clinical features, trio whole genome sequencing was considered to be a more efficient approach for 2 reasons: i) the clinical features linked to this condition had at that stage only been described in a single family and ii) *PI4KA* (ENST00000255882.11) is also a large gene comprising 55 coding exons and lies on 22q11.2 with a total of 16 exons (33-36 and 44-55) overlapping segmental duplications. Recent studies now indicate that the clinical range associated with biallelic *PI4KA* variants is more variable, encompassing hypomyelinating leukodystrophy and intestinal anomalies such as multiple intestinal atresia and combined immunodeficiency [6, 7].

This family was then recruited to the OxClinWGS study (ID: 010PI4K001) for trio WGS. The WGS data shows that the only rare coding region variant detected in *PI4KA* was a synonymous (p.S605S) heterozygous alteration detected in the father only and not transmitted. A genome-wide search for *de novo* variants identified two candidate de novo variants, the first of which was a high confidence c.710A>G; p.(Glu237Gly) in *KIF5C* (ENST00000435030.5), a gene which has been implicated in distinctive malformations of cortical development [8-10]. This variant lies in the kinesin domain, within a region that appears to be constrained against missense mutations (Additional file 1: Fig. S19). More importantly, the variant also involves the same Glu237 codon as other published *de novo* mutations c.710A>T (p.Glu237Val) and c.709G>A (p.Glu237Lys). The c.709G>A (p.Glu237Lys) variant has come up in multiple studies and appears to be a mutational hotspot [8, 9, 11]. Even though the CADD score is slightly lower for p.Glu237Gly (29.3) than for the other published mutations (31/34), it is high enough still to invoke *in silico* support for the variant (ACMG criteria PP3). Seeing a novel missense change at an amino acid residue where a different pathogenic missense change has been seen before is also moderately strong evidence for pathogenicity (PM5). Based on the ACMG guidelines, we therefore consider this variant to be pathogenic (PS2, PM1, PM2, PM5, PP3).

Review of the relevant literature indicates there are some phenotype similarities such the prominent nose and cerebellar abnormalities (see Fig 1a/b in Michels *et al* [9]). The p.(Glu237Val) family described by Poirier *et al* also had arthrogryposis [10]. We also note 3 additional cases with p.(Glu237Lys) in Decipher (IDs 267125, 272993, 284323).

The second high confidence *de novo* mutation ENST00000295888.9:c.2345+1G>A involves another gene *WDFY3* (also called *ALFY*) (Table 2). Data from a large population database (gnomAD v2.1.1) suggest that this gene is intolerant to loss of function alleles with pLI=1.00. A study by Kadir *et al* [12] also suggests this gene is involved with determining brain size and they identified a large pedigree with a p.(Arg2637Trp) variant that co-segregated with microcephaly [12]. Another study of 3 large autism cohorts identified *de novo* mutations in *WDFY3* in 9/4,998 subjects, and in at least one case this was linked to macrocephaly [13]. This gene is listed in OMIM under “?Microcephaly 18, primary, autosomal dominant” (#617520). We are aware of unpublished data from the 100K Genomes Project ([www.genomicsengland.co.uk/initiatives/100000-genomes-project](http://www.genomicsengland.co.uk/initiatives/100000-genomes-project)) that supports this disease-gene association and so our overall interpretation is that the severe clinical presentation in our patient represents a blended phenotype. It would be important to evaluate the quantitative expression of wild type and variant transcripts, to confirm that the splice site variant in *WDFY3* was not leaky, and that any normal transcript was absent, but this was not possible given the termination of pregnancy.

## 010PMA001 – example of extending clinical range (*RMND1*)

A Caucasian, distantly related, consanguineous couple were seen at the genetics clinic following a loss of 3 male pregnancies due to intrauterine growth retardation, arthrogryposis and brain abnormalities of polymicrogyria and cerebellar hypoplasia. They have 2 healthy living children.

Foetal DNA samples were available for 2 affected pregnancies and post-mortem reports were available for the same. In both these pregnancies, there were no concerns until the 16 week scan identified limb and brain abnormalities. The second affected pregnancy was terminated at 28 weeks. The post-mortem findings were: birth weight was 546 g and limb anomalies including talipes, flexion at the knees, clenched hands and overlapping thumbs. Neuropathology examination showed polymicrogyria. The third affected pregnancy was terminated at 29 weeks with a birth weight of 644g. The foetal abnormalities included microcephaly, micrognathia, scoliosis, arthrogryposis, clenched fingers, bilateral talipes and ventricular septal defect. The neuropathology findings (Additional file 1: Fig. S27) were suggestive of a widespread cortical malformation as seen with the previous foetus. Foetal MRI was not carried out in any of the pregnancies.

WGS, through the OxClinWGS programme, identified homozygous variants in *RMND1* (c.108delT, p.Phe36LeufsTer9), which segregated with affected foetus.

## 020CHA001 - example of secondary finding (*FBN1*)

The participant was referred to a neuropathy clinic at the age of 43. She had a history of pes cavus since childhood and found it difficult to find shoes to fit her. She was poor at sports at school and would frequently sprain her ankles. She had required surgical repair of a ligamentous injury to the left ankle the year prior to review. She had noticed very gradually progressive weakness of the intrinsic muscles of the feet and more recently the hands. She also complained of numbness of the feet and intermittent stabbing pain. There was a family history in that her youngest (of three) sons also had high arches and a tendency to trip. Examination findings were consistent with a symmetrical sensorimotor neuropathy. There was bilateral pes cavus and distal muscle wasting and weakness affecting the intrinsic muscles of the hands (grade 4 MRC first dorsal interosseous) and of the ankles and feet (grade 4 MRC ankle dorsiflexion). The ankle reflexes were absent. On sensory examination pin prick, light touch, vibration were reduced to the ankle and proprioception was present but impaired at the metatarso-phalangeal joint. Nerve conduction studies were consistent with a symmetrical, length-dependent, axonal sensorimotor neuropathy. Overall, the clinical presentation was compatible with Charcot-Marie-Tooth type 2.

Prior to having WGS, the patient had the Bristol hereditary neuropathy screen (which at that time had 57 genes linked to CMT) which had identified no pathogenic variants for CMT2. A pathogenic variant, p.(Asn3232fs) in *DST*, a gene known to cause Hereditary Sensory and Autonomic Neuropathy type 6 (HSAN6) was identified by WGS. However, this *DST* variant was not considered to be pathogenic for the patient’s Charcot Marie Tooth type 2 given the distinct phenotype and the fact that, to date, all *DST* variants causing HSAN6 are bi-allelic and we had only identified a pathogenic *DST* variant on one allele; a pertinent genetic finding for this patient has not been identified to date.

A secondary finding, a p.(Asn1422fs) variant in *FBN1*, was however identified in this patient. She had consented to return of secondary findings and was therefore referred to the cardiovascular genetics clinic. She was found to have mild aortic root dilatation (41mm at Sinus of Valsalva) on transthoracic echocardiogram. Physical examination revealed, dental crowding, abnormal upper segment:lower segment and arm span:height ratios, a positive thumb sign, mild chest wall asymmetry, reduced (mild) extension at the elbows, joint hypermobility and scars from her previous foot and ankle surgeries. There was no ectopia lentis. Cascade evaluation of family members was arranged and her son was found to show physical signs of Marfan’s syndrome and genetic testing confirmed that he had inherited the same p.(Asn1422fs) variant in *FBN1*. Both patients are now followed regularly in clinic.

## 021BIL002 – patient with bilateral hippocampal sclerosis (*ALG13*)

This 42-Year-old male developed active epilepsy at the age of 15 years. An MRI scan demonstrated bilateral hippocampal atrophy, more marked on the left and video telemetry suggested seizures likely of right temporal lobe onset. His epilepsy has been pharmacoresistant and he has been prescribed 5 different seizure medications. He went on to have a vagal nerve stimulator implanted in 2016 to control seizures although he continues to have 3-4 focal impaired awareness seizures/month (lasting around 2 mins in duration). He does not have other significant co-morbidities.

WGS identified a variant in *ALG13*, a gene known to be associated with epilepsy. Variants in the glycosylation domain have been associated with epilepsy and the pathogenicity of these can be confirmed using isoelectric focusing of serum transferrin. This test was, however, normal in our patient, a result which can be explained by the fact that our variant is in the ubiquitination (not glycosylation) domain. Expression of the recombinant mutant protein showed no differences from wild type. We still consider this a good candidate and propose to investigate ubiquitination assays to further investigate the potential pathogenicity of this variant.

## 021MTL001 – patient with mesial temporal lobe epilepsy (*APP*)

The female proband presented with intractable localisation-related epilepsy at the age of 11 years old. Bilateral hippocampal sclerosis was confirmed by MRI in 2008 and on repeat imaging in 2016. An interictal EEG confirmed a left posterior temporal lobe focus. The patient suffers from depression and poor memory and perimenstrual migraine. She experiences complex partial seizures – comprising monthly clusters of attacks, up to 10 attacks per day; convulsive seizures and flurries of convulsions, the latter of which usually occur every 1-2 years. There is no family history of epilepsy. The patient has been treated with three anti-epileptic medications. She is considered a possible candidate for resective epilepsy surgery or vagus nerve stimulation (VNS).

We identified a deletion in the *APP* gene, using the SVRare algorithm. This gene is known to be associated with dementia (OMIM #104760) and early-onset familial Alzheimer’s disease, and is implicated in late-onset epilepsy [14]. Recent studies indicate that seizures may contribute to symptoms in the early stage of Alzheimer’s disease [15] and originate in the mesial temporal lobe area of the brain [16]. Since the aetiology of Alzheimer’s arises from aggregates of amyloid-beta (Aβ, encoded by *APP* gene) in the brain, the mechanism by which a deletion could give rise to Aβ aggregation would need further exploration. In addition, the gene has hitherto been associated with late-onset epilepsy, whereas our patient’s epilepsy commenced at a young age. Therefore further investigation of this gene is needed.

## 037CON002 – patient with congenital myasthenic syndromes (*SLC5A7*)

This Kuwaiti patient from a consanguineous family was referred to Oxford from Great Ormond Street Hospital with classic features for a pre-synaptic form of congenital myasthenic syndromes (CMS) including i) ptosis ii) mild restriction to eye movement (ophthalmoparesis) with an intermittent squint iii) hypotonia and marked delay in motor development iv) severe episodic apnoea during intercurrent infections (leading to 3 PICU admissions with ventilation during 1^st^ year) v) decrement on repetitive nerve stimulation shown on electromyography (which is considered diagnostic for a myasthenic disorder at age of the patient).

On the basis of the clinical characteristics, the patient was started on pyridostigmine medication, to which there was an excellent response (again characteristic for many myasthenic disorders), but, as is typical for these patients, not sufficient to completely eliminate the apnoeic episodes.

Based on these clinical features it was suggested that there were likely underlying mutation(s) in *CHAT* or *RAPSN*, which were then screened in Oxford but no abnormal variants were detected. Subsequently other CMS genes were screened (such as *CHRNA1, CHRNB1, CHRND, CHRNE*), but no variants that might cause CMS were uncovered.

Given the clear clinical diagnosis of CMS, but lack of a genetic diagnosis, the patient was put forward for WGS under OxClinWGS programme and the p.(Arg107His) variant in *SLC5A7* was identified. No other variants in CMS genes were identified and the phenotype of this patient exactly matched the phenotypes that were just being published for CMS due to mutations in *SLC5A7* described by Bauche *et al* [17]. The p.(Arg107His) segregated with disease in the family with parents and one sister and normal expression levels of p.(Arg107His) were seen on Western blots, like patients in the Bauche paper with mutations in the vicinity at p.(Pro105Ser), and p.(Tyr111His), although no functional choline uptake assays were undertaken.

The patient's family was given the genetic diagnosis resulting from the WGS, which also gave confidence that the correct treatment regime was applied. The pyridostigmine and salbutamol had the precise clinical effects that would be expected.

## 039FIB001 – example of a genetic diagnosis that enabled a life-saving intervention (*NPHP3*)

Three of 5 siblings from unaffected non-consanguineous parents developed progressive chronic kidney disease in young adulthood, with minimal proteinuria and no haematuria. Renal biopsy in the proband showed non-specific tubulointerstitial disease, with some globally sclerosed glomeruli, but remaining glomeruli histologically unremarkable except for some tuft collapse, and tubular atrophy without cysts. Immunofluorescence stains were negative. Electron microscopy showed normal basement membranes. The clinical differential included autosomal dominant medullary cystic kidney disease / nephronopthisis. At the time of recruitment, the family were ineligible for recruitment to Genomics England 100K Genomes Project due to lack of proteinuria or haematuria (though would have fitted subsequent ‘early onset kidney disease’ eligibility criteria). Clinical testing would have been firstly *UMOD* and *REN* as single gene tests, followed by a renal ciliopathy panel via London North East Regional Genetics laboratory which covered *NPHP3*. Because there was an anticipated need for transplantation, and the clinical phenotype did not point to a specific gene, WGS via OxClinWGS programme was chosen as first-line investigation. Following the confirmation of the *NPHP3* pathogenic compound heterozygous variants, a clinically unaffected brother was confirmed not to carry either the p.(Gly533Asp) or the p.Leu1141Pro variant, and was therefore able to proceed as a successful kidney donor for the proband before he required any dialysis. The proband would otherwise have needed to wait for a kidney on the deceased donor list, with average wait times of 2-3 years, likely necessitating a period on dialysis. 1 year and 5 year survival rates on dialysis are 86% and 63% respectively, compared to 97% / 87% with a deceased donor and 99% / 95% with a living donor (UK Organ Donation and Transplant annual report 2021-22, [www.odt.nhs.uk/statistics-and-reports/annual-activity-report](http://www.odt.nhs.uk/statistics-and-reports/annual-activity-report), [18]).

## 058FANC001 – patient with nephrocalcinosis example of compound heterozygous mutations involving a splice site (*SLC34A1*)

The female patient, born to non-consanguineous parents, presented with neonatal polyuria, polydipsia and failure to thrive. Her blood biochemistry at 17 months of age showed: mild hypercalcaemia (ionized calcium = 1.38 - 1.44 (normal range (NR) = 1.08 - 1.34) mM), total calcium = 10.9 (NR = 8.8-10.8) mg/dL); normal phosphorus = 4.0 – 5.1 (NR = 4.0 – 7.0) mg/dL; low PTH = 12 (NR = 15-55) pg/mL; normal 25-hydroxy-vitamin D = 35.8 (NR = 30-50) ng/mL; elevated 1,25-dihydroxy vitamin D = 80.2 -134.8 (NR 15-80) pg/mL; high alkaline phosphatase = 373 (NR = 95-350) iU/L); high bone specific alkaline phosphatase = 299.8 (NR = 50-150) U/L); low CO2 = 21.4 (NR 23-30) mEq/L; low creatinine = 0.21 (NR = 0.25-0.64) mg/dL; and mild hypoglycaemia = 65 (NR = 70-110) mg/dL. There was trace occult blood and leukocytes in urine. An ultrasound of the kidneys and bladder at 18 months of age revealed generalised increased echogenicity of the renal pyramids bilaterally consistent with advanced stage nephrocalcinosis. Mild left and right pelviectasis was observed. A survey for rickets at 18 months of age showed that osseous structures in the left hand and knee were unremarkable and bony mineralization was normal. She subsequently developed mild hypokalaemia (plasma potassium = 3.8 (NR 3.9-5.7) mEq/L), continued to have a high alkaline phosphatase (342 (NR 64-339) IU/L), and her most recent kidney ultrasound at 10 years of age showed nephrocalcinosis and stable mild pelviectasis. She was identified to have compound heterozygous mutations of the solute carrier family 34 member 1 (*SLC34A1*) gene which encodes the sodium/phosphate cotransporter 2A. Combined analysis with DNA from her parents (Additional file 1: Fig. S20) revealed her to have a paternally inherited c.241dupG variant (p.Glu81fs) and a maternally inherited c.1175-3C>A variant that altered the acceptor splice site sequence from *g****c****ag* to *g****a****ag*, and resulted in utilisation of an alternative splice site within exon 11 and led to an in-frame deletion of 10 amino acids (codons 392-401) located within one of the cytoplasmic topological domains of the sodium-dependent phosphate transport protein 2A. The parents, who are heterozygous for these mutations, do not have nephrocalcinosis or renal disease.

## 066GCD001 - Genetic Cholestatic Disease (*ABCB4*)

This is a female patient that presented with pruritus at the age of 9. Additional exploration revealed hepatomegaly over the left lobe. Splenomegaly was not manifest clinically but was detected radiologically. A liver biopsy showed severe biliary pattern fibrosis, suggesting a form of sclerosing cholangitis or unknown biliary cirrhosis as diagnosis. Seven years later, the patient continued suffering pruritus and showed persistent splenomegaly and jaundice, although there were no signs of chronic liver disease. A new liver biopsy showed biliary fibrosis with small bile duct loss. This led to a diagnosis including primary or secondary sclerosing cholangitis, syndromic or non-syndromic bile duct paucity and progressive familial intrahepatic cholestasis.

Gene panel analysis in the proband identified a stop gain heterozygous variant in *ABCB4* (p.Glu734*). No other pathogenic variants were reported. The variant in *ABCB4* was inherited from her mother, who also has the variant in a heterozygous state. This possibly explains the fact that the mother was cholestatic in pregnancy and is also intermittently itchy. However, the phenotype of the proband is much more severe than the one observed in the mother and it could only be explained by a second pathogenic variant in *ABCB4* (inherited paternally) or another genetic defect elsewhere. The father has no known health conditions.

Our WGS analysis of this trio identified a second variant in *ABCB4* in the patient. The variant is found in a heterozygous state in both patient and father. It is located in the third intron of *ABCB4* (ENST00000649586.2:c.135+26A>G) and predicted to increase splicing at donor position chr7:87472599 in conjunction with loss of splicing at known donor chr7:87472620 (Table 2). No NMD is predicted. This would result in an in-frame insertion of 7 residues from the residue p.L45 of the ABCB4 protein. A minigene experiment has confirmed the presence of the extended transcript created by the splice site variant Additional file 1: Fig. S21).

## 066NNH001 – Neonatal haemochromatosis (*HSD3B7* and *BMP6*)

This female, born prematurely in 2011 at 33 weeks of gestation, presented with neonatal haemochromatosis. She received an orthotopic liver transplant at 37 weeks gestation. She also presented with chronic lung disease and bilateral congenital dislocation of hips, but both were resolved before the patient was referred to the liver unit. The last annual post-transplant review before this referral showed normal liver and renal function, with minimal fibrosis.

The patient was the second child of the couple. Their first child was born healthy and unaffected, which discarded alloimmunity as the cause of the condition observed. The mother of the patient had hereditary haemochromatosis, caused by a known pathogenic mutation in homozygous status in *HFE* gene (p.C282Y), whereas the father had no known health issues. The *HFE* variant was identified in a heterozygous state in the patient as expected, although it does not explain the phenotype observed. In fact, there is no known relationship between Neonatal haemochromatosis and Hereditary haemochromatosis. No other variants were identified in the patient via gene panel test.

Our WGS analysis of this trio could not detect pathogenic variants in coding regions. However, we identified two pairs of compound heterozygous variants in regulatory regions of genes *HSD3B7* and *BMP6* through GREEN-DB annotations. The *HSD3B7* variants are located in a conserved region at the 3’-UTR end of the gene so may affect polyadenylation and stability, hypotheses currently being tested.

## Supplementary References

References

1. Srivastava, N., R. Sudan, and W.G. Kerr, *Role of inositol poly-phosphatases and their targets in T cell biology.* Front Immunol, 2013. **4**: p. 288.

2. So, E.Y., et al., *Lipid phosphatase SHIP-1 regulates chondrocyte hypertrophy and skeletal development.* J Cell Physiol, 2020. **235**(2): p. 1425-1437.

3. Rohrschneider, L.R., et al., *The intron 5/6 promoter region of the ship1 gene regulates expression in stem/progenitor cells of the mouse embryo.* Dev Biol, 2005. **283**(2): p. 503-21.

4. Lefroy, H., T. Goodacre, and U. Kini, *Kapur-Toriello syndrome: a further case report and expansion of the phenotype.* Clin Dysmorphol, 2015. **24**(4): p. 170-2.

5. Pagnamenta, A.T., et al., *Germline recessive mutations in PI4KA are associated with perisylvian polymicrogyria, cerebellar hypoplasia and arthrogryposis.* Hum Mol Genet, 2015. **24**(13): p. 3732-41.

6. Salter, C.G., et al., *Biallelic PI4KA variants cause neurological, intestinal and immunological disease.* Brain, 2021. **144**(12): p. 3597-3610.

7. Verdura, E., et al., *Biallelic PI4KA variants cause a novel neurodevelopmental syndrome with hypomyelinating leukodystrophy.* Brain, 2021. **144**(9): p. 2659-2669.

8. Cavallin, M., et al., *Recurrent KIF5C mutation leading to frontal pachygyria without microcephaly.* Neurogenetics, 2016. **17**(1): p. 79-82.

9. Michels, S., et al., *Mutations of KIF5C cause a neurodevelopmental disorder of infantile-onset epilepsy, absent language, and distinctive malformations of cortical development.* Am J Med Genet A, 2017. **173**(12): p. 3127-3131.

10. Poirier, K., et al., *Mutations in TUBG1, DYNC1H1, KIF5C and KIF2A cause malformations of cortical development and microcephaly.* Nat Genet, 2013. **45**(6): p. 639-47.

11. de Ligt, J., et al., *Diagnostic exome sequencing in persons with severe intellectual disability.* N Engl J Med, 2012. **367**(20): p. 1921-9.

12. Kadir, R., et al., *ALFY-Controlled DVL3 Autophagy Regulates Wnt Signaling, Determining Human Brain Size.* PLoS Genet, 2016. **12**(3): p. e1005919.

13. Wang, T., et al., *De novo genic mutations among a Chinese autism spectrum disorder cohort.* Nat Commun, 2016. **7**: p. 13316.

14. Romoli, M., et al., *Amyloid-beta: a potential link between epilepsy and cognitive decline.* Nat Rev Neurol, 2021. **17**(8): p. 469-485.

15. Cortini, F., C. Cantoni, and C. Villa, *Epileptic seizures in autosomal dominant forms of Alzheimer's disease.* Seizure, 2018. **61**: p. 4-7.

16. Lam, A.D., A.J. Cole, and S.S. Cash, *New Approaches to Studying Silent Mesial Temporal Lobe Seizures in Alzheimer's Disease.* Front Neurol, 2019. **10**: p. 959.

17. Bauche, S., et al., *Impaired Presynaptic High-Affinity Choline Transporter Causes a Congenital Myasthenic Syndrome with Episodic Apnea.* Am J Hum Genet, 2016. **99**(3): p. 753-761.

18. Steenkamp, R., R. Pyart, and S. Fraser, *Chapter 5 Survival and Cause of Death in UK Adult Patients on Renal Replacement Therapy in 2016.* Nephron, 2018. **139 Suppl 1**: p. 117-150.
